# Latent membrane protein 1 and macrophage‐derived TNFα synergistically activate and mobilize invadopodia to drive invasion of nasopharyngeal carcinoma

**DOI:** 10.1002/path.6036

**Published:** 2023-01-06

**Authors:** Wing Chung Tang, Sai Wah Tsao, Gareth E Jones, Xiong Liu, Ming Han Tsai, Henri‐Jacques Delecluse, Wei Dai, Chanping You, Jun Zhang, Shaina Chor Mei Huang, Manton Man‐hon Leung, Tengfei Liu, Yick Pang Ching, Honglin Chen, Kwok Wai Lo, Xin Li, Chi Man Tsang

**Affiliations:** ^1^ Department of Anatomical and Cellular Pathology and State Key Laboratory of Translational Oncology The Chinese University of Hong Kong Hong Kong SAR PR China; ^2^ School of Biomedical Sciences, Li Ka Shing Faculty of Medicine The University of Hong Kong Hong Kong SAR PR China; ^3^ Randall Centre for Cell and Molecular Biophysics King's College London London UK; ^4^ Department of Otolaryngology – Head and Neck Surgery Nanfang Hospital, Southern Medical University Guangzhou PR China; ^5^ Institute of Microbiology and Immunology National Yang‐Ming University Taipei Taiwan; ^6^ Unit F100, DKFZ Heidelberg Germany; ^7^ Inserm Unit U1074, DKFZ Heidelberg Germany; ^8^ Department of Clinical Oncology, Li Ka Shing Faculty of Medicine The University of Hong Kong Hong Kong SAR PR China; ^9^ Guangdong Key Laboratory of Genome Instability and Human Disease Prevention, Department of Biochemistry and Molecular Biology Shenzhen University, School of Medicine Shenzhen PR China; ^10^ Department of Microbiology, Li Ka Shing Faculty of Medicine The University of Hong Kong Hong Kong SAR PR China; ^11^ Shenzhen Key Laboratory of Viral Oncology, The Clinical Innovation & Research Center (CIRC) Shenzhen Hospital, Southern Medical University Shenzhen PR China

**Keywords:** Epstein–Barr virus infection, nasopharyngeal carcinoma, invadopodia, tumor‐associated macrophage, latent membrane protein 1, invasion, live‐cell imaging

## Abstract

Invadopodia are actin‐rich membrane protrusions that digest the matrix barrier during cancer metastasis. Since the discovery of invadopodia, they have been visualized as localized and dot‐like structures in different types of cancer cells on top of a 2D matrix. In this investigation of Epstein–Barr virus (EBV)‐associated nasopharyngeal carcinoma (NPC), a highly invasive cancer frequently accompanied by neck lymph node and distal organ metastases, we revealed a new form of invadopodium with mobilizing features. Integration of live‐cell imaging and molecular assays revealed the interaction of macrophage‐released TNFα and EBV‐encoded latent membrane protein 1 (LMP1) in co‐activating the EGFR/Src/ERK/cortactin and Cdc42/N‐WASP signaling axes for mobilizing the invadopodia with lateral movements. This phenomenon endows the invadopodia with massive degradative power, visualized as a shift of focal dot‐like digestion patterns on a 2D gelatin to a dendrite‐like digestion pattern. Notably, single stimulation of either LMP1 or TNFα could only enhance the number of ordinary dot‐like invadopodia, suggesting that the EBV infection sensitizes the NPC cells to form mobilizing invadopodia when encountering a TNFα‐rich tumor microenvironment. This study unveils the interplay of EBV and stromal components in driving the invasive potential of NPC via unleashing the propulsion of invadopodia in overcoming matrix hurdles. © 2022 The Authors. *The Journal of Pathology* published by John Wiley & Sons Ltd on behalf of The Pathological Society of Great Britain and Ireland.

## Introduction

Cancer metastasis is a major cause of cancer morbidity and mortality, accounting for ~90% of cancer‐related deaths [1]. Cancer cells near the basement membrane can develop invasive properties to realize distal metastasis. These cells can digest the extracellular matrix (ECM) and penetrate the basement membrane, allowing them to intravasate into the bloodstream, travel to distal niches, and form metastatic tumors [2, 3]. One strategy adopted by invasive cells to escape from the primary tumor is the formation of invadopodia, which can protrude into, and degrade, the surrounding matrix [2, 3]. These actin‐rich protrusions are regulated by cytoskeletal modulators and scaffolding proteins and are associated with the local release of matrix metalloproteinases (MMPs), allowing them to degrade the ECM and facilitate cellular migration through the basement membrane [4, 5].

Nasopharyngeal carcinoma (NPC) is a highly invasive and metastatic head‐and‐neck cancer with a high prevalence in Southern China, including Hong Kong [6, 7, 8]. It is an infection‐related cancer driven by the Epstein–Barr virus (EBV) [9, 10, 11]. A defining clinical characteristic of NPC that distinguishes it from other head‐and‐neck cancers is more frequent metastasis to cervical lymph nodes and distal organs [12, 13]. An EBV‐encoded protein, latent membrane protein 1 (LMP1), is a well‐known mediator of the invasive properties of NPC cells and can promote NPC metastasis by several different mechanisms [14, 15]. LMP1 can induce the expression of Snail to promote epithelial‐to‐mesenchymal transition (EMT) in NPC cells [16]. It can also upregulate the expression of matrix metalloproteinase 9 (MMP9), which correlates with lymph node metastases in NPC [17]. Furthermore, LMP1 enhances the formation of focal adhesion (FA) complexes and tumor necrosis factor α‐induced protein 2 (TNFAIP2)‐associated membrane protrusions to promote epithelial cell migration [18, 19].

The tumor microenvironment (TME) plays a significant role in tumor progression. Heavy infiltration of lymphocytes and tumor‐associated macrophages (TAMs) is a common histopathological feature of undifferentiated NPC [20]. TAMs are significantly involved in cancer‐related inflammation [21]. These macrophages form a phenotypic continuum from M1‐like or classically activated macrophages, which are pro‐inflammatory and anti‐tumor, to M2‐like or alternatively activated macrophages, which are anti‐inflammatory, immunosuppressive, pro‐angiogenic, and pro‐tumor. During the pathogenesis of NPC, continuous interactions between the EBV‐infected premalignant/malignant nasopharyngeal cells and TAMs may contribute to the development of invasive properties.

In NPC, the interplay between EBV‐infected tumor cells and the infiltrating macrophages, and how this modulates the formation of invadopodia, has not yet been studied. Since TAMs were found to positively correlate with the prognosis of NPC [22], it has been suggested that TAMs play a crucial role in facilitating NPC metastasis. In this study, we co‐cultured M1‐ or M2‐polarized macrophages with EBV‐positive NPC cells and observed a robust upregulation of invadopodia formation in the NPC cells. TNFα was found to mediate this macrophage‐induced invadopodia formation. Moreover, conditioned media from M1‐ or M2‐polarized macrophages could induce the expression of EBV‐encoded LMP1, potentiating the formation of invadopodia. Live‐cell imaging was used to reveal invadopodia dynamics in the NPC cells. Interestingly, the invadopodia formed in the cells co‐activated by viral and stromal factors produced a dendrite‐like gelatin degradation pattern, whereas a dot‐like degradation pattern was observed in cells activated by LMP1 or TNFα alone. Potential pathways and proteins involved in promoting the formation, degradative power, and migratory properties of invadopodia in NPC were also explored.

## Materials and methods

### Cell cultures

EBV‐positive and ‐negative NPC43 and NP460hTert cell lines established by our laboratory were used in this study [23, 24]. The M81 strain of EBV (EBV‐M81) was provided by Dr HJ Delecluse [25]. The culturing conditions of those and other common cell lines including HEK293T/Phoenix cells and THP‐1 cells are included in Supplementary materials and methods.

#### Differentiation and polarization of M1‐/M2‐like macrophages from the THP‐1 cell line and human primary monocytes

THP‐1 cells and primary monocytes were differentiated into macrophages in a Transwell (0.4 μm pore size, 30 mm diameter; Millipore, Billerica, MA, USA). The differentiation and polarization of M1‐/M2‐like macrophages from THP‐1 and primary monocytes were performed as described previously in [26] and [27], respectively. Details of the procedures are provided in Supplementary materials and methods.

#### Live‐cell imaging microscopy

Confocal images were acquired using confocal microscopes LSM800, LSM880, or LSM900 (Carl Zeiss, Thornwood, NY, USA). GE IN Cell Analyzer 6500HS (GE Healthcare, Waukesha, WI, USA) was used for real‐time high‐content imaging. SYTO 41 (5 μm; Thermo Fisher Scientific, Inc., Waltham, MA, USA) was used for nucleus visualization. The area of FITC‐gelatin digested per cell was measured every 30 min using GE IN Cell Analyzer v7.3. The invadopodia tracks were analyzed using the Imaris tracking program (Bitplane, South Windsor, CT, USA). Time‐coded videos of invadopodia‐digested areas of gelatin were rendered in Fiji [28]. Additional details are provided in Supplementary materials and methods.

#### Identifying the cytokines released from M1‐like macrophages

The M1‐like macrophage conditioned medium was collected and applied to a cytokine antibody array (RayBiotech, Peachtree Corners, GA, USA) following the manufacturer's instructions. The cytokines detected using the antibody array were purchased and used to treat the NPC43^EBV+ve^ cells individually for invadopodia assays at physiological concentrations (details provided in supplementary material, Table S1).

#### Transient transfection, plasmids, cloning of DNA constructs, and mutagenesis

To overexpress plasmids with fluorescent tags, transient transfection was accomplished using X‐tremeGENE HP DNA transfection reagent (Roche, Indianapolis, IN, USA) according to the manufacturer's instructions. Plasmids containing sequencing of LMP1 and LifeAct‐mCherry (a kind gift from Professor Michael Way [29]) were used for transduction. Q5® Polymerase [New England BioLabs (NEB), Ipswich, MA, USA] and T4 ligase (NEB) were used for DNA cloning. The sequence of the *SRC* gene CDS region was cloned from HEK293T cell cDNA. Src mutants (Y419F, Y530F) were created using a Site‐Directed Mutagenesis Kit (NEB) with desired sequences (supplementary material, Table S1) following the manufacturer's instructions. The PLPCX 2117LMP1 was generated in our laboratory previously [30]. The plasmids and sequences used for cloning are listed in supplementary material, Table S1. Further details are provided in Supplementary materials and methods.

#### Immunoprecipitation assay

The cell lysate containing Cdc42‐GTP was incubated with 20 μl of glutathione‐*S*‐transferase (GST)‐Cdc42‐binding domain (CBD) of N‐WASP binding beads (a kind gift provided by Professor Gareth Jones) as described by Liu *et al* [31]. Further details are provided in Supplementary materials and methods.

#### Western blotting

Cell lysates were extracted using RIPA buffer. The samples were electrophoresed in SDS‐PAGE gels and transferred to a polyvinylidene difluoride membrane. Details of the protocol and antibodies used are provided in Supplementary materials and methods.

#### RNAscope (RNA *in situ* hybridization to detect CD68 and TNF)

RNA transcripts of *CD68* and *TNF* in NPC tissue were detected using an RNAscope 2.5 HD Duplex Reagent Kit (ACDBio, Newark, CA, USA) following the manufacturer's instructions. Probes to detect *CD68* (560591‐c2; ACDBio) and *TNF* (310421; ACDBio) were used. A mixture of the probes (*TNF* probes:*CD68* probes, 50:1) was added to the slide for 2 h at 40 °C. A series of AMP buffers were used to amplify the signal, and the corresponding substrates were used to visualize the *CD68* and *TNF* RNA transcripts. Finally, the slide was counterstained with hematoxylin and mounted.

#### Bulk RNA‐Seq dataset analysis of M1‐/M2‐like macrophages in NPC tissue

An RNA‐Seq dataset for bulk NPC tumors was obtained from the Gene Expression Omnibus (GEO) (Accession No. GSE68799). The clean reads were aligned to the human reference genome GRCh38 from GENCODE (Version 27) [32]. The transcripts per million (TPM) were calculated by RSEM (v1.3.0) [33]. RSeQC (2.6.4) and Picard (2.17.4) were used for quality control [34, 35]. The prevalence of M1‐ and M2‐like macrophages in the bulk tumors was estimated by CIBERSORT using the default parameters [36]. A total of three healthy samples and 41 NPC samples were included in this analysis. The differences in cell composition between healthy and NPC samples were evaluated using the Mann–Whitney *U*‐test. The significance level was set at *p* < 0.05.

#### Image analysis and statistics

Image handling, quantification, and analysis of fluorescence images were performed using ZEN 2.3 (Blue edition, Carl Zeiss), IN Cell Analyzer v7.3 (GE Healthcare), Imaris 9.5.1 (Oxford Instruments, Concord, MA, USA), or ImageJ (Fiji v. 2.0.0‐rc‐65/1.51 w) software [37]. The figure legends describe the exact number of independent replicates that were analyzed in each experiment. The data presented include all measured data points for all experiments, including means ± standard error of the mean (SEM), or bar charts. Statistical analysis was performed using the means of at least three individual experiments, and statistical significance was determined by unpaired *t*‐tests using Prism 8 (GraphPad Software Inc., San Diego, CA, USA). All *P* values less than 0.05 were considered significant.

Detailed procedures for performing gelatin coating, flow cytometry analysis, immunofluorescence microscopy, immunohistochemistry, inhibition of cell signaling pathways, and bioinformatic analysis of RNA‐Seq are provided in Supplementary materials and methods.

## Results

### Both M1‐ and M2‐polarized macrophages enhance invadopodia formation in NPC

**Figure 1:**
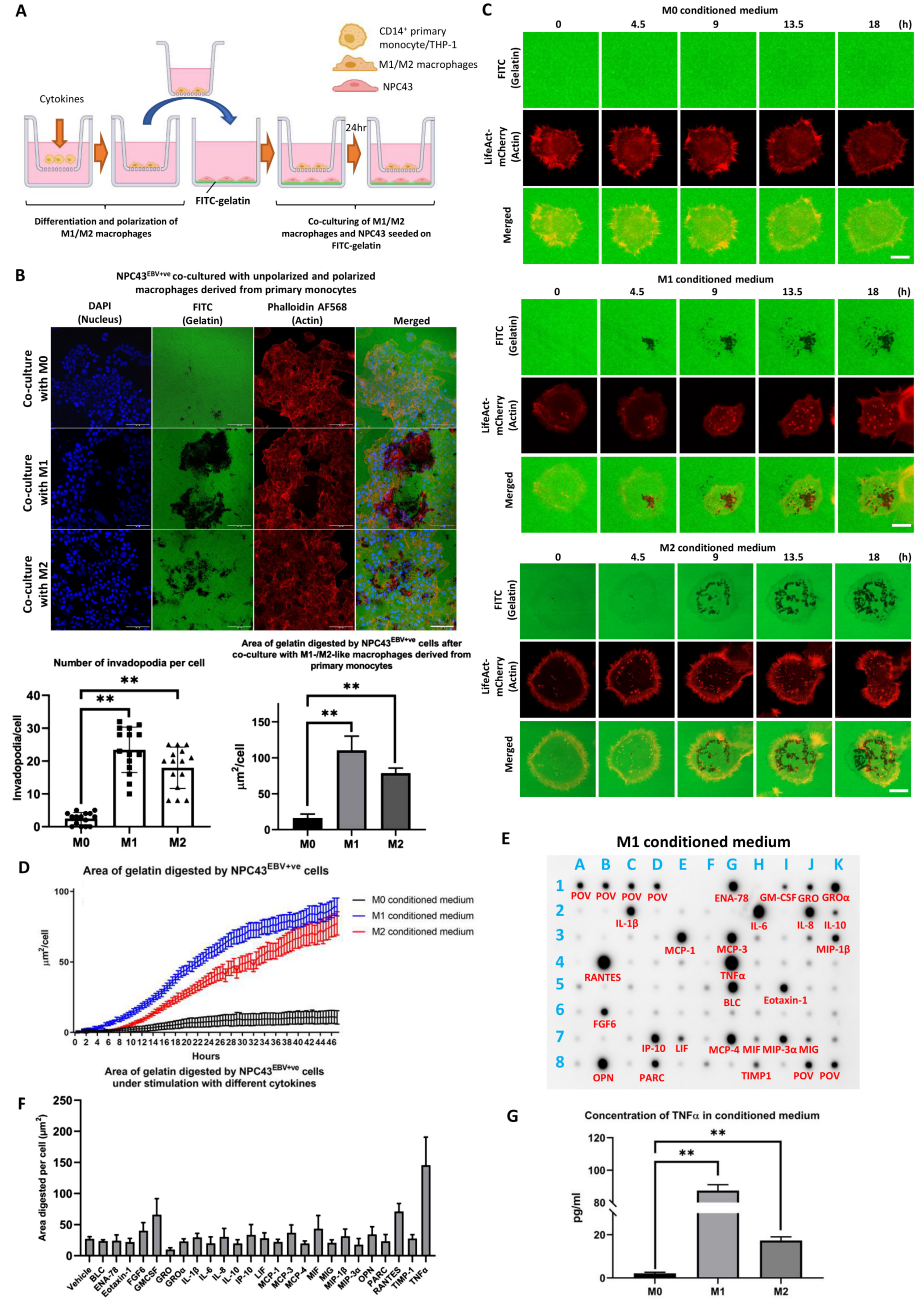
Both primary monocyte‐ and THP‐1‐derived TAMs enhance invadopodia formation and gelatin degradation in NPC43^EBV+ve^ cells. (A) Experimental setup for monocyte polarization and co‐culturing of NPC43 cells with polarized macrophages. Monocytes were seeded in a Transwell insert and incubated with cytokines to promote polarization. The Transwell containing the polarized macrophages was then transferred to another well containing FITC‐gelatin pre‐seeded with NPC43^EBV+ve^ cells. (B) Top: confocal images of FITC‐gelatin digested by NPC43^EBV+ve^ cells after co‐culture with polarized M1‐/M2‐like macrophages derived from primary monocytes. Scale bar: 100 μm. Bottom: the number of invadopodia per cell and the area of FITC‐gelatin digested were calculated. (C) Real‐time confocal images of NPC43^EBV+ve^ cells forming invadopodia induced by M1‐/M2‐like macrophage conditioned media derived from THP‐1 cells for 18 h. Both the number of invadopodia and the area of FITC‐gelatin digested increased in a time‐dependent manner. Scale bar: 5 μm. (D) Real‐time statistical analysis of the area of FITC‐gelatin digested per NPC43^EBV+ve^ cell from 15 random positions (each containing ~30 cells) under different conditioned medium treatments using high‐content microscopy over 48 h. The digested area per NPC43^EBV+ve^ cell induced by M1‐ or M2‐like macrophage conditioned medium was 89.0 ± 6.3 and 77.4 ± 8.2 μm^2^/cell, respectively, after 48 h of digestion. (E) Detection of cytokines secreted into the culture medium of M1‐like macrophages using an antibody array. Each dot represents the presence of a single cytokine. (F) The area of digested FITC‐gelatin per NPC43^EBV+ve^ cell under the stimulation of each of the 25 upregulated cytokines individually. TNFα induced the largest area of digested FITC‐gelatin. (G) The concentration of TNFα in M1‐/M2‐like macrophage conditioned media was detected using an ELISA. The concentrations of TNFα secreted by M0‐, M1‐, and M2‐like macrophages were 2.1 ± 0.5, 87.4 ± 3.6, and 17.3 ± 1.7 pg/ml, respectively. Means ± SEM. Student's *t*‐test *P* value indicated the significant difference among the compared groups (**p < 0.01). Each of the above experiments was repeated three times (*N* = 3).

To demonstrate the clinical relevance of M1‐/M2‐like macrophages in modulating the TME of NPC, we assessed the proportion of cells with M1 or M2 macrophage markers using data derived from RNA sequencing of clinical NPC samples. Comparable percentages of M1 and M2 macrophages were seen in NPC stroma (supplementary material, Figure S1A), suggesting that both types of macrophages could interact with NPC *in vivo*. To investigate the role of TAMs in tumor invasion, both primary human monocytes and a monocytic leukemia cell line (THP‐1) were used to generate differentiated M1‐ and M2‐like macrophages using standard protocols [26, 27]. The differentiated M1‐ and M2‐like macrophages expressed their corresponding markers of CD80 and CD163, respectively (supplementary material, Figure S1B). The differentiated macrophages derived from primary or THP‐1 monocytes were co‐cultured inside a Transwell insert with NPC43^EBV+ve^ cells pre‐seeded on FITC‐gelatin (Figure 1A). After 24 h of co‐culture with the macrophages, the number of invadopodia and the area of FITC‐gelatin digested per cell were assessed using confocal imaging (Figure 1B and supplementary material, Figure S1C). A significant increase in the number of invadopodia and digested area per cell was observed after co‐culture with M1‐/M2‐like macrophages. Given that there was no direct contact between the M1‐/M2‐like macrophages and the NPC43^EBV+ve^ cells, we hypothesized that the gelatin‐degrading activity of the NPC cells was induced by macrophage‐derived cytokines. Thus, the experiment was repeated using M0, M1‐/M2‐like macrophage conditioned media, which showed a similar result (Figure 1C). Importantly, the live‐cell imaging videos illustrate the formation of invadopodia as punctate dots of actin (supplementary material, Video S1). Real‐time quantification of the FITC‐gelatin digested per cell using high‐content screening was carried out by randomly capturing 15 images (each containing ~30 cells) of each condition in a 96‐well plate (Figure 1D). The results indicated that conditioned media from M1‐/M2‐like macrophages could induce FITC‐gelatin degradation by the NPC cells.

### M1‐/M2‐like macrophages induce invadopodia formation in NPC43^EBV^ ^+ve^ cells via TNFα secretion

**Figure 2:**
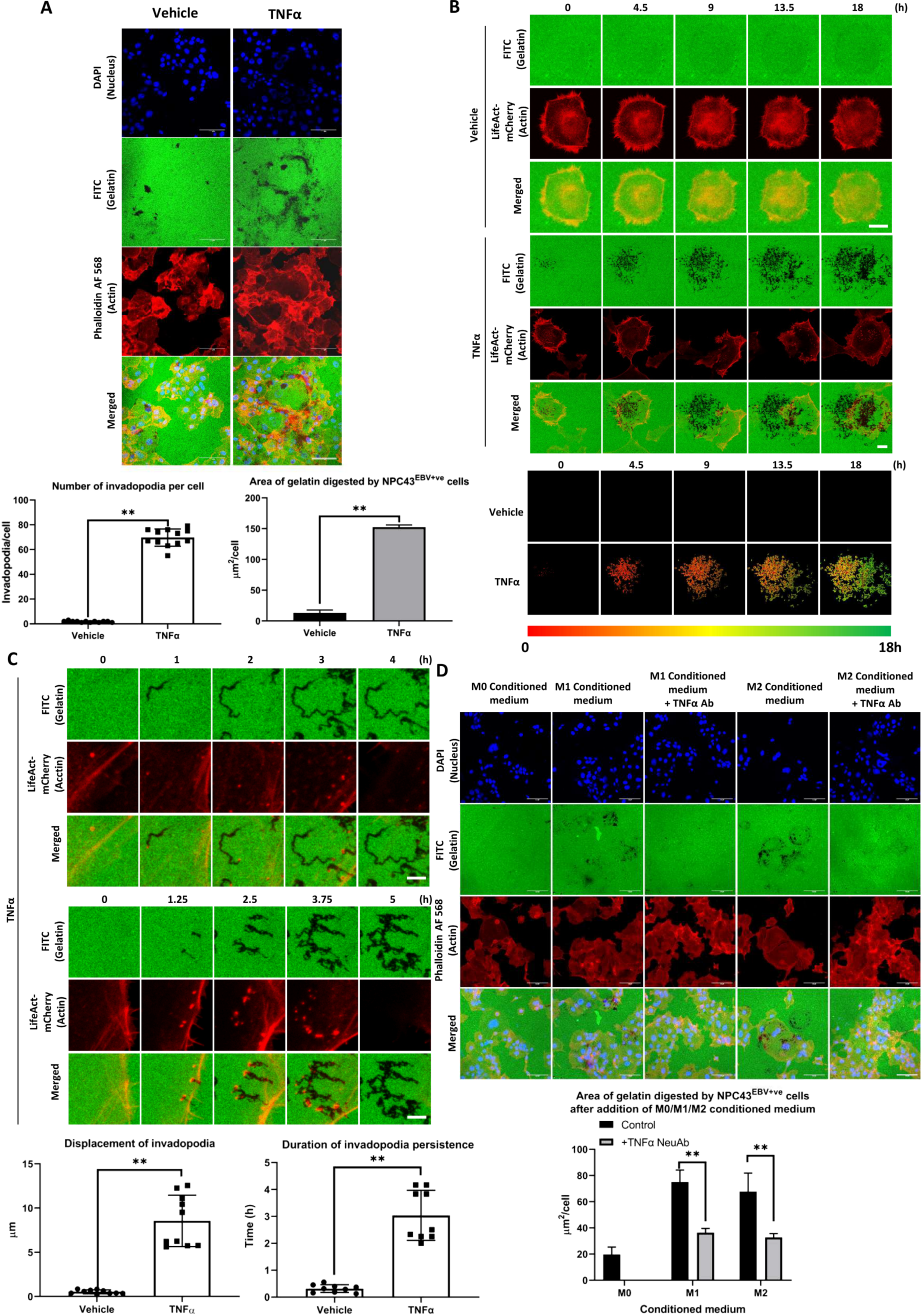
M1‐/M2‐like macrophages induce invadopodia formation in NPC43^EBV+ve^ cells via secretion of TNFα. (A) Top: confocal images of NPC43^EBV+ve^ cells digesting the FITC‐gelatin under the stimulation of TNFα (10 ng/ml). TNFα could potently induce the digestion of FITC‐gelatin by NPC43^EBV+ve^ cells. Scale bar: 100 μm. Bottom: statistical analysis of the number of invadopodia per cell and the area of gelatin degradation induced by TNFα treatment. TNFα could induce an average of 69.6 ± 6.9 invadopodia in NPC43^EBV+ve^ cells. (B) Top: real‐time confocal images of NPC43^EBV+ve^ cells forming TNFα‐induced invadopodia. Scale bar: 5 μm. Bottom: accumulated areas digested by the invadopodia of vehicle or TNFα‐treated NPC43^EBV+ve^ cells in a time‐dependent manner are shown. The cumulative digested areas were temporally color‐coded across different time frames and stacked to form one image. Red = 0 h, yellow = 9 h, and green = 18 h. (C) Top: confocal time‐lapse microscopy of a selected invadopodium from B showing lateral movement accompanied by the digestion of FITC‐gelatin. Middle: confocal microscopy of the branched formation of invadopodia from B. Scale bar: 1 μm. Bottom: statistical analyses of the total displacement and the lifespans of invadopodia with or without treatment with TNFα are shown. Invadopodia induced by TNFα travelled 8.5 ± 2.9 μm, while the control invadopodia only travelled 0.54 ± 0.223 μm. The invadopodia induced by TNFα persisted for 3.0 ± 0.9 h, while those in control cells persisted only for 0.3 ± 0.1 h. (D) Top: conditioned media from M1‐/M2‐like macrophages were incubated with TNFα neutralizing antibodies for 30 min prior to being added to the NPC43^EBV+ve^ cells and assaying invadopodia formation. The TNFα‐neutralizing antibody‐containing conditioned media induced less FITC‐gelatin degradation by NPC43^EBV+ve^ cells compared with the conditioned media alone. Scale bar: 100 μm. Bottom: statistical analysis of the area of FITC‐gelatin digested per cell. Means ± SEM. Student's *t*‐test *P* value indicated the significant difference among the compared groups (**p < 0.01). Each of the above experiments was repeated three times (*N* = 3).

Given that our data indicated that invadopodia formation in NPC43^EBV+ve^ cells was stimulated through cytokine(s) secreted into the culture medium, M1‐like macrophage culture medium was collected and examined using a cytokine antibody array. A total of 25 cytokines were detected (Figure 1E). The 25 cytokines were added individually to NPC43^EBV+ve^ cells and invadopodia formation was assayed. TNFα induced the greatest area of FITC‐gelatin digestion per cell (145.4 ± 45.2 μm^2^/cell) (Figure 1F). Both M1‐ and M2‐like macrophages produced TNFα, although the expression level of TNFα was lower for M2‐like macrophages (Figure 1G). The significant ability of TNFα to induce actin‐rich invadopodia formation is illustrated in Figure 2A,B and supplementary material, Video S2. The invadopodia formed under the TNFα treatment were identified by invadopodia markers including cortactin, TKS5, and N‐WASP (supplementary material, Figure S2A). The invadopodia generated a dendrite‐like pattern of gelatin degradation (Figure 2B and supplementary material, Video S3). Our data strongly suggest that TNFα is a potent inducer of invadopodia with high gelatin degradative activity. Most TNFα‐induced invadopodia persisted for several hours with high digestive ability (Figure 2C and supplementary material, Figure S2B). Intriguingly, the invadopodia of TNFα‐treated cells had a higher displacement along the horizontal plane of the FITC‐gelatin than those of untreated cells (Figure 2C). Examining individual invadopodia in the TNFα‐treated cells revealed that some traveled long distances along the horizontal plane, leaving a long worm‐like track of degraded gelatin (Figure 2C and supplementary material, Video S4). In fact, some invadopodia could even split into daughter invadopodia, resulting in multiple digestive tracks (Figure 2C and supplementary material, Video S5). These unique digestive patterns were firstly reported in human cancer cells. TNFα‐neutralizing antibodies were added to M1/M2 conditioned media for 30 min at 37 °C prior to their addition to the NPC43^EBV+ve^ cell culture (Figure 2D). Significantly downregulated FITC‐gelatin digestion was observed, indicating that TNFα likely mediates M1‐/M2‐induced invadopodia formation in NPC43^EBV+ve^ cells (Figure 2D). To examine whether human macrophages express TNFα *in vivo* in NPC tissue, tumor biopsies collected from NPC patients were sectioned and stained with a TNFα antibody. Strong and diffuse staining was observed (supplementary material, Figure S2C), indicating that TNFα was secreted and dispersed around the tumor islets and stromal areas. RNAscope was used to trace the source of TNFα (supplementary material, Figure S2D). NPC tissues were co‐stained with probes to detect their corresponding RNA transcripts: *CD68* (macrophage marker) and *TNF*. The results showed that CD68^+^ cells expressed *TNF* in NPC tissues (supplementary material, Figure S2D), indicating that macrophages are a main source of TNFα.

### TNFα activates EGFR–Src–ERK–cortactin signaling pathways

**Figure 3:**
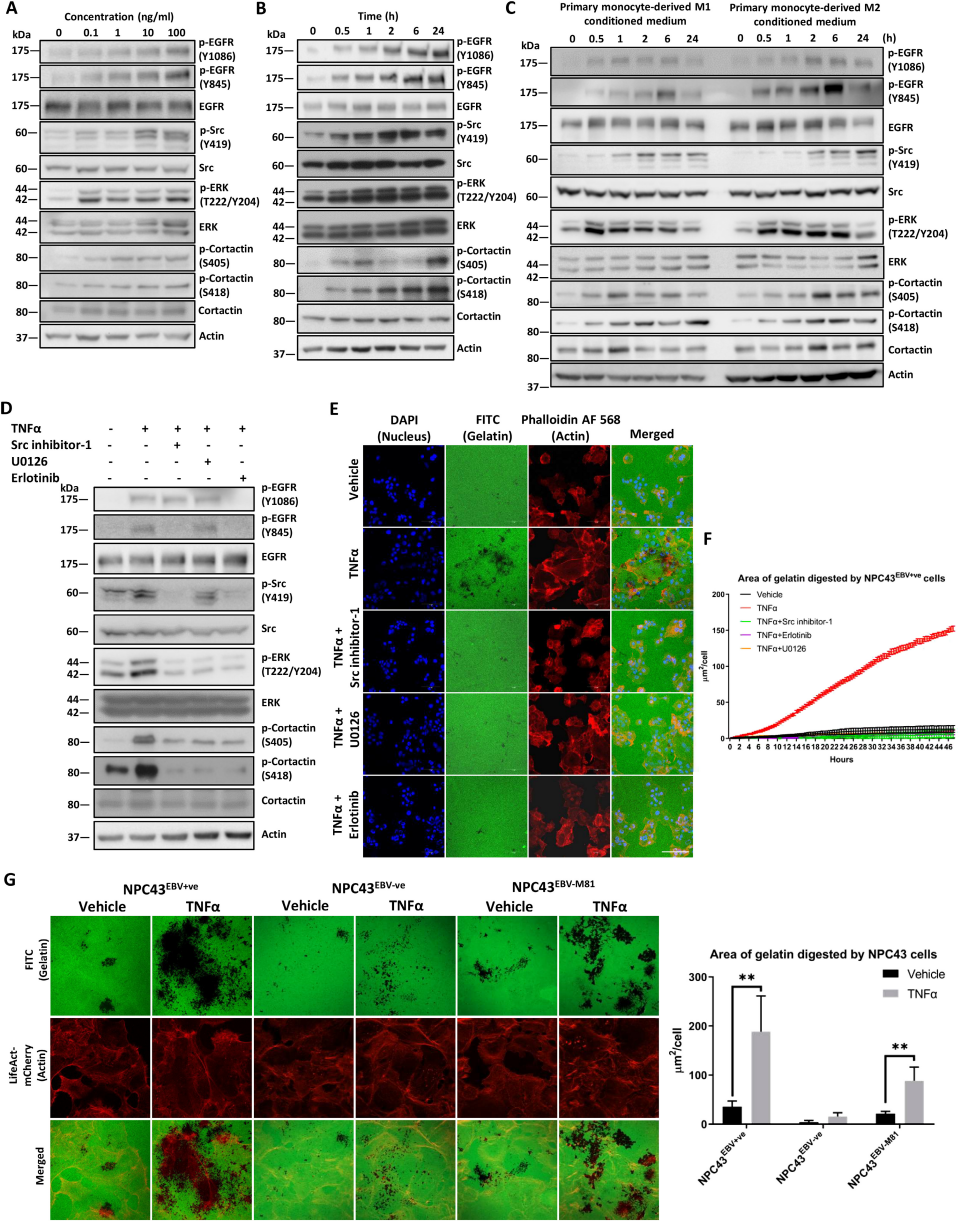
TNFα‐induced phosphorylation of invadopodia‐associated signaling proteins. (A) Western blot analysis of EGFR, Src, ERK, and cortactin phosphorylation in NPC43^EBV+ve^ cells treated with different concentrations of TNFα (0.1–100 ng/ml) for 24 h. Phosphorylation of EGFR, Src, ERK, and cortactin increased in a dose‐dependent manner. (B) Western blot analysis of EGFR, Src, ERK, and cortactin phosphorylation in NPC43^EBV+ve^ cells treated with TNFα (10 ng/ml) at different time points. Phosphorylation of EGFR, Src, ERK, and cortactin increased in a time‐dependent manner. (C) Western blot analysis of EGFR, Src, ERK, and cortactin phosphorylation in NPC43^EBV+ve^ cells treated with primary monocyte‐derived macrophage conditioned media at different time points. The pattern of phosphorylation of EGFR, Src, ERK, and cortactin resembles that seen with TNFα stimulation. (D) Western blot analysis showing the effect of different inhibitors (erlotinib, Src inhibitor‐1, and U0126) and TNFα stimulation on the phosphorylation of EGFR, Src, ERK, and cortactin. (E) Confocal images illustrating inhibited invadopodia formation with the inhibitors (erlotinib, Src inhibitor‐1, and U0126) prior to the addition of TNFα to NPC43^EBV+ve^ cells. The digestion of FITC‐gelatin by NPC43^EBV+ve^ cells was suppressed by these inhibitors. Scale bar: 100 μm. (F) Real‐time statistical analysis of the digested area of FITC‐gelatin per NPC43^EBV+ve^ cell from 15 random positions with different inhibitor treatments and TNFα exposure using high‐content screening microscopy over 48 h. (G) Left: confocal images of NPC43 cells with or without EBV infection treated with TNFα. The NPC43^EBV+ve^ and NPC43^EBV‐M81^ cells responded to TNFα treatment and digested a greater area of FITC‐gelatin compared with NPC43^EBV−ve^ cells. Scale bar: 100 μm. Right: statistical analysis of the digested area per cell. Quantification of the band signals in western blots of A–D is shown in supplementary material, Figure S3E–H. Means ± SEM. Student's *t*‐test *P* value indicated the significant difference among the compared groups (**p < 0.01). Each of the above experiments was repeated three times (*N* = 3).

To understand the signaling pathways involved in TNFα‐induced invadopodia formation, western blot analysis was used to detect the phosphorylation of EGFR, Src, ERK, and cortactin, all of which are highly associated with invadopodia formation [38, 39]. Increased phosphorylation of EGFR, Src, ERK, and cortactin was observed following TNFα treatment in both dose‐ (0.1–100 ng/ml) and time‐ (0–24 h) dependent manners and the result aligns with the treatment of M1/M2 conditioned medium (Figure 3A–C and supplementary material, Figure S3A). To further investigate the phosphorylation activated by TNFα, inhibitors including erlotinib, Src inhibitor‐1, and U0126 were added to NPC43^EBV+ve^ cells for 15 min prior to treatment with TNFα (Figure 3D). Western blot analysis showed that EGFR and Src cross‐inhibit each other and are common upstream signaling proteins to ERK and cortactin phosphorylation. Inhibition of ERK by U0126 reduced phosphorylation of cortactin at Ser405 and Ser418 but did not affect EGFR or Src phosphorylation (Figure 3D). Notably, all three inhibitors suppressed the downstream effector, p‐cortactin (S405 and S418). Real‐time live‐cell imaging showed that the area of gelatin digested per cell was significantly suppressed by treatment with each of the inhibitors individually (Figure 3E,F). To demonstrate that Src activation regulated invadopodia formation, the wild‐type (wt), inactivated (Y419F), and activated (Y530F) forms of Src were cloned into a bicistronic plasmid, pEF1α‐IRES‐ZsGreen1. The NPC43^EBV+ve^ cells were then transiently transfected with the plasmids and invadopodia formation was assayed. The activated Src (Y530F) induced more than 100 invadopodia per cell, whereas the wt Src induced only ~50 invadopodia per cell (supplementary material, Figure S3B). Western blot analysis confirmed that wt Src and Src (Y530F) overexpression increased Src phosphorylation (supplementary material, Figure S3C). These results indicate that Src activation could potently increase the number of invadopodia produced by NPC cells. Cortactin and its phosphorylated forms (S405 and S418) under TNFα stimulation were also shown to be present within the actin core of invadopodia in TNFα‐treated NPC cells (supplementary material, Figure S3D).

### EBV infection sensitizes NPC cells to TNFα‐induced invadopodia formation

We have clearly demonstrated that the addition of TNFα can robustly enhance the formation of invadopodia in NPC43^EBV+ve^ cells. We sought to investigate whether EBV infection plays a role in governing the formation of invadopodia. A subclone of NPC43 lacking the EBV genome established in our laboratory (unpublished data) was used. These NPC43^EBV−ve^ cells were subsequently reinfected with the M81‐EBV strain, which was isolated from an NPC patient [25]. The NPC43^EBV+ve^, NPC43^EBV−ve^, and NPC43^EBV‐M81^ cell lines were used to examine the effect of TNFα on the formation of invadopodia (Figure 3G). TNFα greatly enhanced the invadopodia formation in NPC43^EBV+ve^ and NPC43^EBV‐M81^ cells but not in NPC43^EBV−ve^ cells (Figure 3G). We also performed RNA sequencing to assess the expression profiles of genes associated with invadopodia formation in NPC43^EBV−ve^ and NPC43^EBV+ve^ cell lines under TNFα treatment (supplementary material, Figure S4A). Genes that are known to be crucial to invadopodia formation and activity, including TLN1, WAS, WIPF1, VCL, CFL1, MMP9, and MMP14, were highly upregulated in TNFα‐treated NPC43^EBV+ve^ cells, while these genes were only slightly upregulated in the TNFα‐treated NPC43^EBV−ve^ cells. These results suggest that EBV infection modulates the ability of TNFα to induce invadopodia formation.

### EBV‐encoded LMP1 activates the Cdc42/N‐WASP signaling pathway during invadopodia formation

**Figure 4:**
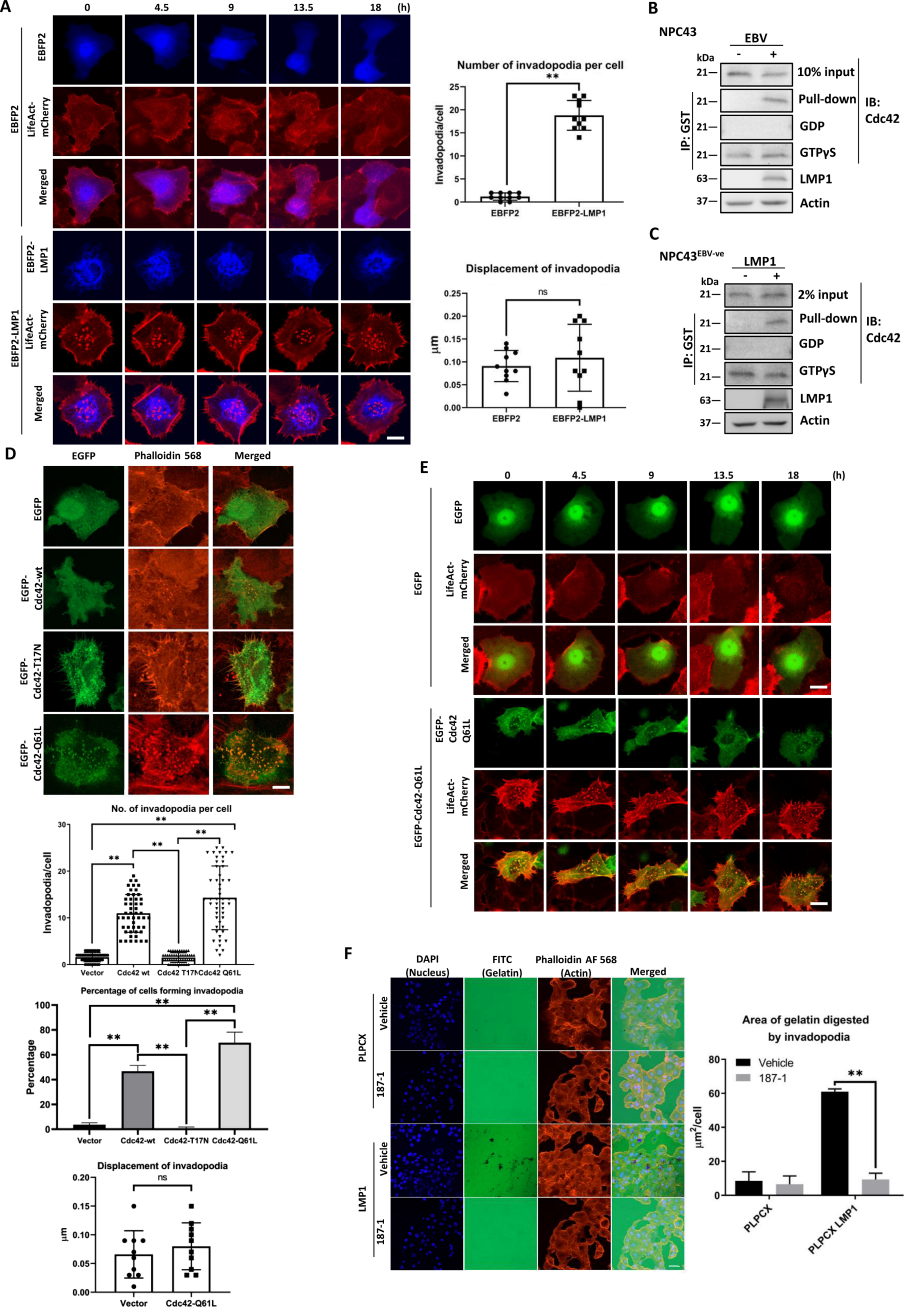
LMP1 induces invadopodia formation in NPC43^EBV+ve^ via the Cdc42/N‐WASP signaling pathway. (A) Left: confocal images of NPC43^EBV−ve^ cells transiently transfected with either control plasmid or EBFP2‐LMP1. EBFP2‐LMP1 could induce invadopodia formation, but LMP1 did not co‐localize with actin. Scale bar: 5 μm. Right: statistical analysis of the number of invadopodia and the displacement of invadopodia induced by EBFP2‐LMP1. EBFP2‐LMP1 could induce 18.80 ± 3.22 invadopodia per cell compared with only 1.21 ± 0.79 invadopodia formed in the control. No significant movement of invadopodia was induced by EBFP2‐LMP1 when compared with TNFα treatment (as shown in Figure 2C). (B) Pull‐down assay using the Cdc42‐binding domain (CBD) of N‐WASP in NPC43^EBV−ve^ and NPC43^EBV+ve^ cells. In EBV‐positive NPC43 cells, LMP1 was expressed and activated Cdc42 could be pulled down. (C) Western blot analysis showing the pull‐down of activated Cdc42 in NPC43^EBV−ve^ cells after LMP1 overexpression. (D) Top: confocal images of NPC43^EBV+ve^ cells transiently transfected with EGFP‐Cdc42‐wt, ‐T17N, or ‐Q61L. EGFP‐Cdc42‐Q61L could potently induce invadopodia formation. Scale bar: 5 μm. Bottom: statistical analysis of the number of invadopodia, the percentage of cells forming invadopodia, and the displacement of invadopodia induced by Cdc42 wt and its mutants. (E) Time‐lapse confocal images of NPC43^EBV+ve^ cells transiently transfected with either control plasmid or EGFP‐Cdc42‐Q61L. Scale bar, 10 μm. (F) Left: 187‐1 (10 μm), an inhibitor of N‐WASP, could reduce invadopodia formation in LMP1‐expressing NPC43^EBV+ve^ cells. Scale bar: 100 μm. Right: statistical analysis of the digested area of FITC‐gelatin per cell. IP, immunoprecipitation; GST, glutathione‐*S*‐transferase; IB, immunoblotting; GDP, guanosine diphosphate; GTP, guanosine triphosphate. Means ± SEM. Student's *t*‐test *P* value indicated the significant difference among the compared groups (**p < 0.01). Each of the above experiments was repeated three times (*N* = 3).

LMP1 is an EBV‐encoded protein well known for promoting the invasiveness of NPC cells [14, 15, 40]. Therefore, we first investigated if LMP1 could enhance the formation and activity of invadopodia. A plasmid expressing the EBFP2‐LMP1 sequence was generated and transiently transfected into the NPC43^EBV−ve^ cells (Figure 4A). Time‐lapse experiments indicated that LMP1 could promote invadopodia formation, but it did not co‐localize with the actin core (Figure 4A and supplementary material, Video S6). Similarly, upregulated formation of invadopodia could also be seen in LMP1‐overexpressing premalignant nasopharyngeal cells (NP460hTert) (supplementary material, Figure S4B). This is the first report to show that LMP1 can induce the formation of invadopodia. The invadopodia induced by LMP1 were identified with invadopodia markers including cortactin, TKS5, and N‐WASP (supplementary material, Figure S4C). We also overexpressed LMP2 in NPC43^EBV−ve^ cells, but there was no gelatin digestion induced (supplementary material, Figure S4D).

LMP1 has been shown to induce the activation of Cdc42 to increase the invasiveness of NPC [31]. Moreover, Cdc42 acts upstream to activate N‐WASP, which is an essential mediator of invadopodia generation. Thus, we postulated that LMP1 could induce invadopodia formation via the Cdc42/N‐WASP signaling pathway. We first assessed Cdc42/N‐WASP activation in NPC43^EBV+ve^ and NPC43^EBV−ve^ cells (Figure 4B,C). Glutathione Sepharose beads bound with the Cdc42/Rac‐interactive binding (CRIB) domain of N‐WASP was used to pull down activated Cdc42. NPC43^EBV+ve^ cells had endogenous LMP1 expression and activated Cdc42 (Figure 4B). Next, in NPC43^EBV−ve^ cells, LMP1 was expressed by retroviral infection, and we showed that LMP1 could activate Cdc42/N‐WASP (Figure 4C).

We then examined the role of Cdc42 in invadopodia formation. EGFP‐Cdc42‐wt, EGFP‐Cdc42‐T17N (inactivated), and EGFP‐Cdc42‐Q61L (activated) were transiently transfected into NPC43 cells, and the invadopodia were observed using a confocal microscope (Figure 4D). Both the wt and the activated forms of Cdc42 could effectively enhance invadopodia formation (Figure 4D). Similarly, Cdc42 could also potently induce the generation of invadopodia in NP460hTert (supplementary material, Figure S4E). Time‐lapse imaging showed that the formation of invadopodia could be sustained in NPC43 cells with stable activation of Cdc42 (Figure 4E and supplementary material, Video S7). The activated Cdc42 formed a ring structure surrounding the actin cores of the invadopodia (supplementary material, Figure S4F).

To further confirm that LMP1 can induce invadopodia formation through N‐WASP, an N‐WASP inhibitor (187‐1) was used to treat NPC43^EBV−ve^ cells transduced with PLPCX (vector) or PLPCX‐LMP1. It was found that 187‐1 could significantly reduce invadopodia formation, indicating that LMP1 induces invadopodia formation through Cdc42 and N‐WASP activation (Figure 4F).

### TNFα and LMP1 synergistically promote invadopodia formation and horizontal mobility

**Figure 5:**
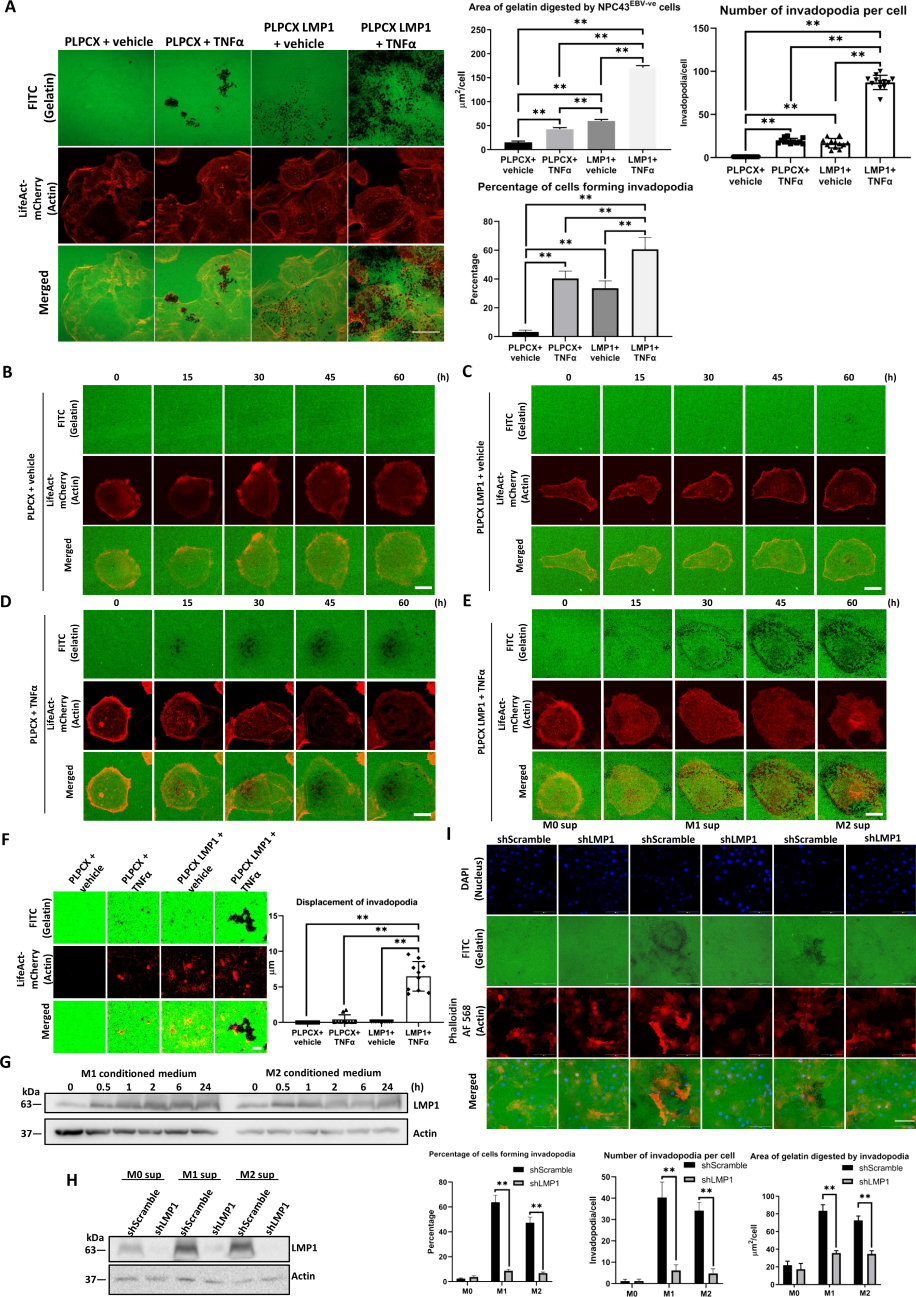
TNFα and LMP1 exert a synergistic effect on invadopodia formation and increase the mobility of invadopodia. (A) Left: confocal images of NPC43^EBV−ve^ cells with or without LMP1 expression treated with vehicle or TNFα (10 ng/ml). The NPC43^EBV−ve^ cells under the dual stimulations of LMP1 and TNFα digested the greatest area of FITC‐gelatin. Scale bar: 20 μm. Right: statistical analysis of the digested area of FITC‐gelatin, and the number of invadopodia per NPC43^EBV−ve^ cell treated with TNFα and/or expressing LMP1, as well as the percentage of cells forming invadopodia. (B–E) Time‐lapse confocal images showing invadopodia formation and gelatin degradation by single NPC43^EBV−ve^ cells treated with TNFα and/or expressing LMP1. Scale bar: 5 μm. (F) Left: magnified images from B–E showing the digestion path of an individual invadopodium under the corresponding condition. Scale bar: 0.5 μm. Right: displacement of invadopodia stimulated by TNFα or/and LMP1. The average displacement of invadopodia induced by co‐stimulation with TNFα and LMP1 was 6.5 ± 2.1 μm. (G) Western blot analysis showing that M1‐/M2‐like macrophage conditioned media increased the expression of LMP1 in NPC43^EBV+ve^ cells in a time‐dependent manner. (H) Western blot analysis showing that M1‐/M2‐like macrophage conditioned media increased the expression of LMP1 in NPC43^EBV+ve^ cells but not in those with shLMP1. (I) Top: knockdown of LMP1 could suppress the invadopodia formation of NPC43^EBV+ve^ cells treated with M1‐/M2‐like macrophage conditioned media. Scale bar: 100 μm. Bottom: statistical analysis of the digested area of FITC‐gelatin per cell, the number of invadopodia per cell, and the percentage of cells forming invadopodia. Protein quantification of G and H corresponds to supplementary material, Figure S5D and S5I. Means ± SEM. Student's *t*‐test *P* value indicated the significant difference among the compared groups (**p < 0.01). Each of the above experiments was repeated three times (*N* = 3).

We have shown that exogenous expression of LMP1 can upregulate invadopodia formation in both NPC and premalignant nasopharyngeal cells. We then sought to investigate whether TNFα‐induced invadopodia formation is potentiated in LMP1‐expressing cells. NPC43^EBV−ve^ cells stably expressing the control vector or LMP1 were treated with vehicle or TNFα (Figure 5A). Either TNFα or LMP1 alone could enhance both the formation of invadopodia and gelatin degradation (Figure 5A). Notably, a synergistic upregulation of invadopodia formation was seen when the cells were stimulated with both TNFα and LMP1. A similar experiment was carried out with the NP460hTert cells (supplementary material, Figure S5A). Again, the largest gelatin degradation area per cell was observed for NP460hTert cells stimulated with both LMP1 and TNFα (supplementary material, Figure S5A). To examine the dynamics of invadopodia formation and gelatin degradation more closely, the TNFα‐treated or/and LMP1‐expressing cells were examined using time‐lapse microscopy at the single‐cell level (Figure 5A–E and supplementary material, Video S8a and Figure S5B,C). Within the 60 h time course, both the LMP1‐expressing cells and the TNFα‐treated cells generated fewer invadopodia (~20) compared with the co‐stimulated cells (>80 invadopodia). The effect of LMP1 and TNFα also increased the percentage of activated invadopodia and prolonged the persistence of invadopodia (supplementary material, Figure S5B,C). Another intriguing phenomenon was that the invadopodia formed by the co‐stimulated cells exhibited high mobility across the horizontal plane of the gelatin (Figure 5F and supplementary material, Video S8b). Due to this, the invadopodia produced by the co‐stimulated cells had large, extending degradation tracks (supplementary material, Video S8b). In contrast, for the singly stimulated cells, the invadopodia remained stationary and dissipated locally to reform in another location and initiate a new round of gelatin degradation (supplementary material, Video S8b).

### M1‐/M2‐like macrophages upregulate the expression of LMP1 to effectively promote invadopodia formation

We have demonstrated that the addition of TNFα can promote the formation of invadopodia, and LMP1 expression can potentiate these effects (Figure 5A–E). Therefore, we hypothesized that interactions between EBV‐infected NPC cells (expressing LMP1) and stromal cells (producing TNFα) would lead to synergistic formation of invadopodia by the cancer cells. Surprisingly, M1/M2 conditioned medium further increased the expression of LMP1 in NPC43^EBV+ve^ cells (Figure 5G). We have also established a pair of EBV‐infected and non‐infected NP460hTert cell lines [24]. The NP460hTert‐EBV cells did not exhibit basal expression of LMP1, but macrophage‐conditioned medium could significantly increase LMP1 expression (supplementary material, Figure S5E). However, neither TNFα nor other cytokines alone could induce LMP1 expression (supplementary material, Figure S5F,G). Alternatively, the NPC cells could not induce TNFα expression in macrophages (supplementary material, Figure S5H). The molecular mechanisms underlying the enhanced LMP1 expression in EBV‐infected NPC cells treated with M1/M2 conditioned media remain to be elucidated. Nevertheless, to illustrate the role of LMP1 in promoting invadopodia formation, LMP1 was knocked down by viral transduction of shLMP1 in the NPC43^EBV+ve^ cells (Figure 5H). The invadopodia‐forming abilities of NPC43‐shLMP1 cells and the vector control cell line with or without treatment with M1/M2 conditioned media were observed using microscopy (Figure 5I). FITC‐gelatin degradation was strongly reduced in the LMP1‐knockdown cells. Transiently transfected Cdc42 in NPC43‐shLMP1 cells regained the FITC‐gelatin degradation, indicating that Cdc42 is a downstream effector of LMP1 (supplementary material, Figure S5J).

### Synergistic formation of invadopodia occurs via co‐activation of TNFα‐Src/p‐cortactin and LMP1–Cdc42/N‐WASP signaling pathways

**Figure 6:**
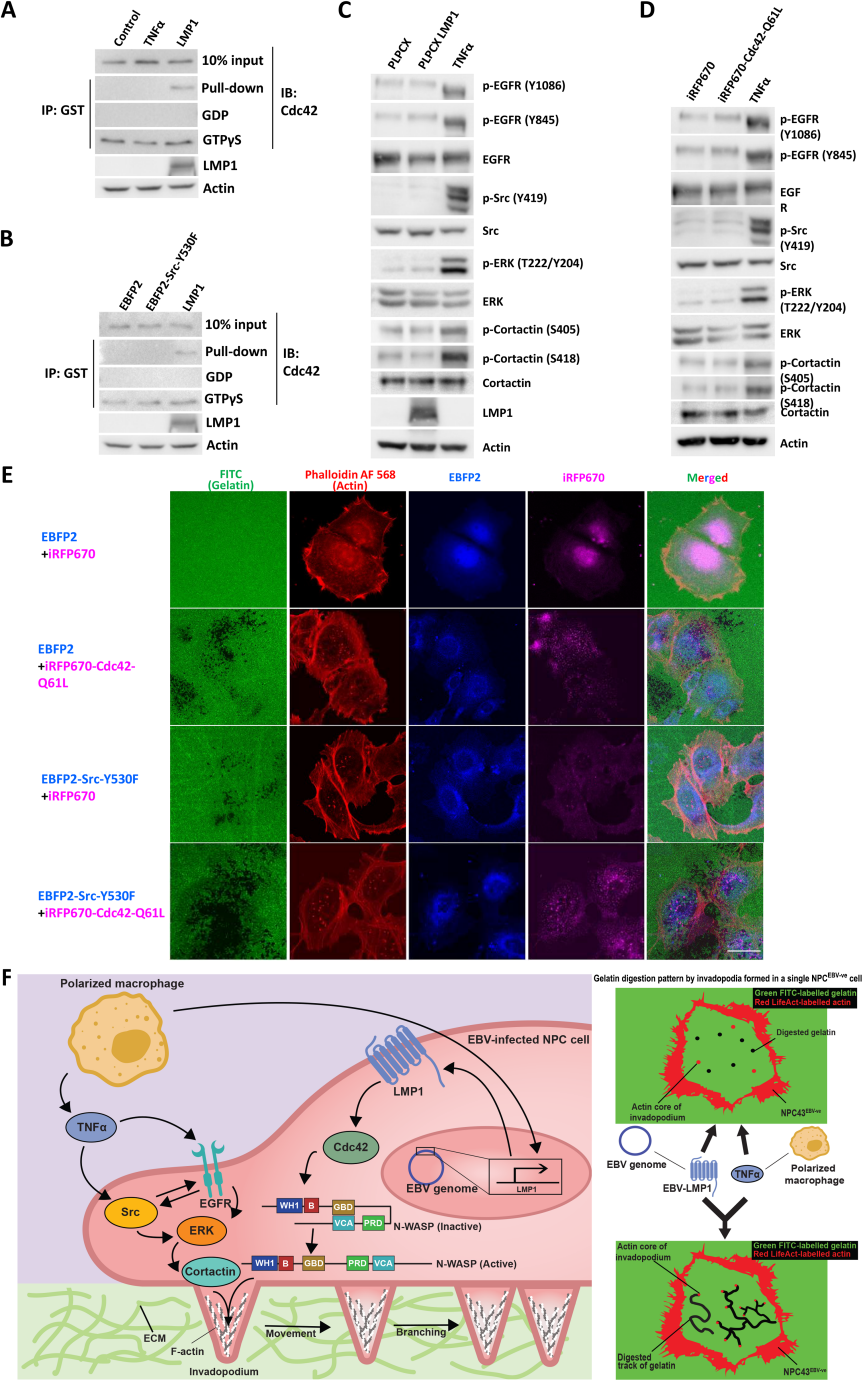
Co‐activation of TNFα–Src–p‐cortactin and LMP1–Cdc42/N‐WASP signaling axes exerts a synergistic effect on invadopodia formation. (A) GST pull‐down showing that TNFα could not activate Cdc42 in NPC43^EBV−ve^ cells. (B) GST pull‐down showing that the transient transfection of NPC43^EBV−ve^ cells with EBFP2‐Src‐Y530F could not activate Cdc42. (C) Western blot analysis showing that LMP1 could not induce the phosphorylation of EGFR, Src, ERK, or cortactin. (D) Western blot analysis showing that iRFP670‐Cdc42‐Q61L could not induce the phosphorylation of EGFR, Src, ERK or cortactin. (E) Confocal images of NPC43^EBV−ve^ cells transiently transfected with EBFP2‐Src‐Y530F and/or iRFP670‐Cdc42‐Q61L. The co‐transfected cells digested the greatest area of gelatin. Scale bar: 20 μm. IP, immunoprecipitation; GST, glutathione‐*S*‐transferase; IB, immunoblotting. Each of the above experiments was repeated three times (*N* = 3). Protein quantification of C and D corresponds to supplementary material, Figure S6A and S6B. (F) Schematic diagram of this study showing how a TAM induces invadopodia formation in an EBV‐infected NPC cell through secreting TNFα and how upregulation of LMP1 activates the Cdc42/N‐WASP pathway. Each pathway could induce dot‐like, non‐moving invadopodia in an NPC cell. The synergistic effect of the two pathways could induce mobile, dendritic‐like invadopodia in an NPC cell.

In this study, we found that the dual activation of TNFα‐mediated and LMP1‐mediated signaling axes could induce significant formation of mobile, degradative invadopodia in NPC43^EBV−ve^ cells. We then further assessed whether TNFα could cross‐activate the N‐WASP pathway and whether LMP1 could cross‐activate the cortactin pathway. Our results indicate that neither treatment with TNFα nor transfection with the activated form of Src could activate the N‐WASP pathway (Figure 6A,B). Additionally, neither LMP1 expression nor activated Cdc42 could activate the EGFR/Src/ERK/cortactin pathway (Figure 6C,D and supplementary material, Figure S6A,B). This suggests that these two regulatory pathways are independently activated by TNFα and LMP1. To observe whether activated Src and Cdc42 could induce invadopodia formation synergistically, EBFP2‐Src(Y530F) and iRFP670‐Cdc42(Q61L) were transiently transfected individually and co‐transfected into NPC43^EBV−ve^ cells. We found that both EBFP2‐Src(Y530F) and iRFP670‐Cdc42(Q61L) alone could induce the formation of invadopodia. (Figure 6E). Importantly, when the NPC cells were co‐transfected with both plasmids, a synergistic effect was seen (Figure 6E). A summary of the mechanisms underlying the synergistic formation of invadopodia under the stimulation of EBV oncogene and stromal factors is shown in Figure 6F.

## Discussion

EBV‐associated NPC has a highly invasive and metastatic nature. Most NPC patients (60–70%) present with advanced disease (stages III and IV) at the time of diagnosis [12, 41]. Despite effective first‐line treatment with chemoradiation, more than one third of NPC patients eventually experience recurrences with distal metastases [41]. Invadopodia are present in cells capable of crossing extracellular barriers [4, 5]. Recent publications have confirmed that invadopodia are required for intravasation and extravasation for metastatic spread of cancer cells [5, 42, 43]. In this study, we strived to explore the role of the TME in modulating EBV‐associated NPC cells, invadopodia formation, and their matrix degradative properties. Previous studies have shown that TAMs support NPC progression and correlate with low survival rates in NPC patients [22, 44]. However, how the TAMs promote distal metastasis of NPC cells remains unknown. This study reveals that TNFα is a major effector, which can be released by both M1‐like and M2‐like macrophages, in promoting the invasive behavior of NPC cells via the promotion of invadopodia formation (Figures 1 and 2). It is well understood that the anti‐inflammatory, M2‐like macrophages promote tumor growth and metastasis [45]. However, this may not be the case in the development of infection‐related cancers [46]. M1‐like macrophages might mediate inflammatory responses to promote tumor initiation and progression. Examples of such infection‐related cancers include hepatitis B virus (HBV)‐associated hepatocellular carcinoma (HCC) and *Helicobacter pylori*‐associated gastric carcinoma. The presence of tumor necrosis factor alpha (TNFα), which is associated with M1 TAM polarization, in the TME has been reported to promote carcinogenesis [47, 48]. Cooperative interactions between HBV and TNFα were shown to play important roles in the activation of NF‐κB and the expression of metabolic pathway‐associated genes, which promote the pathogenesis of HCC [49]. Moreover, macrophage‐derived TNFα can promote Wnt/β‐catenin signaling and may contribute to tumor development in *Helicobacter*‐infected gastric mucosa [50]. In fact, several different populations of macrophages with diverse phenotypes may be required during carcinogenesis. In several large‐scale transcriptomic analyses of cancer tissues, tumor‐infiltrating macrophages have a mixed phenotype, expressing both M1 and M2 markers [46, 51]. Moreover, specific ablation of either M1 or M2 macrophages has not been achieved in any experimental setting, making their specific roles in tumorigenesis unclear. In this study, TNFα was found to be secreted by both types of macrophages and could promote the formation of invadopodia by both NPC cells and premalignant nasopharyngeal cells (NP460hTert).

A signaling axis consisting of TNFα–EGFR–SRC–ERK–cortactin was shown to mediate invadopodia generation (Figure 3A–F). The mechanisms underlying TNFα‐mediated activation of these signaling pathways and how these signaling pathways crosstalk to phosphorylate cortactin in NPC cells warrant further studies. TNFα can stimulate transactivation of the EGFR signaling pathway to promote cell survival in colonic epithelial cells [52]. EGFR can mediate the Src activation in A431 epidermal carcinoma cells [53], while cortactin is a well‐known substrate of ERK1/2 and Src family kinases in the regulation of invadopodia formation [54]. Previous publications have shown that phosphorylation of cortactin by EGFR, Src, and ERK can promote cortactin activity, actin polymerization, and invadopodia formation [38, 55]. Consistent with the central role of cortactin in regulating invadopodia formation, clinical studies have shown that cortactin overexpression is associated with local invasion, lymph node metastasis, and/or distal metastases [55, 56].

The fact that the robust TNFα‐induced invadopodia formation could be observed only in EBV‐positive NPC cells (Figure 3G) suggests that an EBV‐encoded component plays a crucial role in responding to the inflammatory tumor environment and governing the invasive properties of NPC cells. Among the EBV‐encoded proteins, LMP1 has been found to be involved in NPC invasion. LMP1 can increase epithelial cell motility and invasion by regulating the actin cytoskeleton and inducing the expression of MMPs [15, 16, 17]. Cdc42, a small GTPase, is one of the downstream targets of LMP1 and regulates cell migration [31]. Cdc42 is known to mediate cellular invasive properties such as focal complex formation, integrin localization, and MMP expression. It is also a key component in the activation and formation of invadopodia [57]. Notably, the Cdc42/N‐WASP signaling axis was activated in the LMP1‐expressing cells to promote invadopodia formation (Figure 4). Association of GTP‐bound Cdc42 with N‐WASP promotes a conformational change in the N‐WASP molecule, revealing its VCA domain [58]. Taken together, this is the first report to show that LMP1 is involved in invadopodia formation through the activation of Cdc42 and its downstream effector N‐WASP.

Lastly, live‐cell imaging in this study revealed a horizontal displacement of the invadopodia in the *Z*‐plane of the gelatin treated with the EBV/LMP1‐positive cells activated by TNFα, whereas with the cells stimulated by TNFα or LMP1 alone, the invadopodia formed and retracted locally, resulting in a dot‐like pattern of gelatin degradation (Figures 2C and 5F). Additionally, some invadopodia appeared to branch to form two or more newly formed daughter invadopodia, resulting in dendritic tracks of gelatin degradation. These novel patterns may be due to rapid actin nucleation by the activated cortactin and N‐WASP in the peripheral region of the actin core of the invadopodia. Nevertheless, Helgeson and Nolen have demonstrated that a synergistic activation of the Arp2/3 complex by cortactin and N‐WASP promotes actin nucleation [33]. In response to certain signals, actin can assemble into linear filaments or can form branches with one end anchored to an existing filament. Branch formation requires the Arp2/3 complex, which initiates and anchors branches to existing filaments, in addition to various nucleation‐promoting factors (NPFs), which promote the branching activity of the Arp2/3 complex. Two types of NPFs have been identified: type I NPFs (e.g. N‐WASP) interact with individual actin molecules, while type II NPFs (e.g. cortactin) bind to actin filaments. These two types of NPFs cooperate to create branches using an ‘obligatory displacement’ model. According to this model, before more actin molecules can be added, N‐WASP must be released either slowly on its own or by being rapidly displaced by cortactin. In our system, LMP1‐expressing cells have elevated levels of GTP‐bound Cdc42, which can promote the open configuration of N‐WASP and expose the VCA domain. Concurrently, TNFα activates cortactin to promote rapid displacement of N‐WASP. The synergistic nucleation of actin may drive the rapid formation of actin bundles at the lateral side of the actin core of the invadopodia. Therefore, the actin core of the invadopodia does not dissipate, but rather becomes laterally displaced. This lateral displacement and increased invadopodia mobility result in an extensive degradation of gelatin. We believe that in the inflammatory TME of NPC, stromal macrophages provide TNFα to stimulate the EBV/LMP1‐postive NPC cells and promote their degradative power for dissolving the ECM and permitting metastasis.

In conclusion, we report here that the invadopodia‐forming abilities of EBV‐positive NPC cells depend on an intrinsic interaction with the inflammatory stroma via the secretion of TNFα from macrophages. TNFα–EGFR–Src–ERK–cortactin and LMP1–Cdc42–N‐WASP signaling pathways enhance invadopodia formation and matrix degradation. These two pathways are activated independently but act synergistically to intensify invadopodia formation and matrix degradation in NPC. This study provides the basis for the development of translational studies that could examine the effectiveness of the dual targeting of tumor and stromal cells. This will open new avenues for the development of new, effective therapies to prevent the metastasis of NPC cells in patients, addressing a major clinical problem in the treatment of this invasive cancer.

## Author contributions statement

WT, CY, TL, JZ, and CT designed, performed, and interpreted the experiments. ST, GJ, KL, XL, and CT gave valuable input on structuring the experiments. XL, MT, HD, SH, YC, and HC provided and prepared valuable materials for experiments. WD and ML helped in data analysis. WT and CT wrote the manuscript and put the figures together.

## Supporting information


Supplementary materials and methods
**Figure S1.** Polarized M1‐/M2‐like macrophages induced invadopodia information in NPC43^EBV+ve^ cells. Related to Figure 1
**Figure S2.** Macrophage‐derived TNFα could induce invadopodia information in NPC43^EBV+ve^ cells. Related to Figure 2
**Figure S3.** TNFα, EGFR, Src, ERK, and cortactin are essential for invadopodia formation. Related to Figure 3
**Figure S4.** LMP1 and its downstream Cdc42 could induce invadopodia formation. Related to Figure 4
**Figure S5.** LMP1 and TNFα synergistically induce invadopodia in NP460 cells. Related to Figure 5
**Figure S6.** Quantification of the western blots of Figure 6
**Table S1.** Key resources

**Video S1.** NPC43^EBV+ve^ co‐culture with conditioned medium of M0, M1, or M2 macrophages

**Video S2.** NPC43^EBV+ve^ co‐culture with vehicle or TNFα

**Video S3.** Accumulated areas digested by the invadopodia of TNFα‐treated NPC43^EBV+ve^ cells

**Video S4.** Confocal time‐lapse microscopy of a selected invadopodium showing lateral movement accompanied by the digestion of FITC‐gelatin

**Video S5.** Confocal microscopy of the branched formation of invadopodia

**Video S6.** EBFP2‐LMP1 induced invadopodia formation in NPC43^EBV−ve^ cells

**Video S7.** EGFP‐Cdc42‐Q61L induced invadopodia formation in NPC43^EBV+ve^ cells

**Video S8a.** TNFα and LMP1exert a synergistic effect on invadopodia formation and increase the mobility of invadopodia

**Video S8b.** Magnified live‐cell images of Video S8a to show the movement of invadopodia

## Data Availability

The RNA‐Seq dataset for bulk NPC tumors was obtained from the Gene Expression Omnibus (GEO) (Accession No. GSE68799). The RNA‐Seq dataset for NPC43^EBV+ve^ and NPC43^EBV−ve^ cells treated with or without TNFα was also obtained from the Gene Expression Omnibus (GEO) (Accession No. GSE218167).
