# Supplementary material for: Latent membrane protein 1 and macrophage‐derived TNFα synergistically activate and mobilize invadopodia to drive invasion of nasopharyngeal carcinoma
Source: J Pathol. 2023 Jan 6;259(2):163–79. doi: 10.1002/path.6036 (PMC10108171; doi:10.1002/path.6036)
Supplement: Supplementary file 1 — Supplementary materials and methods Figure S1. Polarized M1‐/M2‐like macrophages induced invadopodia information in NPC43EBV+ve cells. Related to Figure 1 Figure S2. Macrophage‐derived TNFα could induce invadopodia information in NPC43EBV+ve cells. Related to Figure 2 Figure S3. TNFα, EGFR, Src, ERK, and cortactin are essential for invadopodia formation. Related to Figure 3 Figure S4. LMP1 and its downstream Cdc42 could induce invadopodia formation. Related to Figure 4 Figure S5. LMP1 and TNFα synergistically induce invadopodia in NP460 cells. Related to Figure 5 Figure S6. Quantification of the western blots of Figure 6 Table S1. Key resources [file PATH-259-163-s010.doc]

**Latent membrane protein 1 and macrophage-derived TNFα synergistically activate and mobilize invadopodia to drive invasion of nasopharyngeal carcinoma**

WC Tang *et al. J Pathol* <https://doi.org.10.1002/path.6036>

**Supplementary materials and methods**

**Supplementary Figures S1–S6**

**Supplementary Table S1**

**Supplementary materials and methods**

Reference numbers refer to the main text list

*Cell cultures*

EBV-positive and -negative NPC43 and NP460hTert cell lines established by our laboratory were used in this study [23,24]. The M81 strain of EBV (EBV-M81) was provided by Dr HJ Delecluse [25]. EBV-positive, EBV-negative, and EBV-M81 NPC43 cells were cultured in RPMI (Sigma-Aldrich, Carlsbad, CA, USA) supplemented with 20 μm ROCK inhibitor, 10% FBS, and 1% penicillin–streptomycin stock solution (PS). The HEK293T/Phoenix cells were cultured in DMEM (Sigma-Aldrich) supplemented with 10% FBS and 1% PS. The THP-1 cell line was an immortalized monocyte-like cell line derived from the peripheral blood of a childhood case of acute monocytic leukemia (M5 subtype) [59]. The THP-1 and human primary monocytes were cultured in RPMI 1640 medium with 10% heat-inactivated FBS and 1% penicillin–streptomycin stock solution. All cell lines were incubated in a humidified incubator at 37 °C with 5% CO_2_.

*Differentiation and polarization of M1-/M2-like macrophages from the THP-1 cell line*

THP-1 cells were seeded at a density of 1 × 10^6^ cells/ml on a Transwell (0.4 μm pore size, 30 mm diameter; Millipore, Billerica, MA, USA) which was hung in a six-well plate containing 2 ml of medium per well. To induce differentiation into M1-/M2-like macrophages, THP-1 cells were first treated with phorbol 12-myristate 13-acetate (PMA, 200 ng/ml; Sigma) overnight. The next day, to induce polarization of M1-like macrophages, lipopolysaccharides (LPS, 100 ng/ml; Sigma) and interferon gamma (IFNγ, 20 ng/ml; GenScript, Piscataway, NJ, USA) were added to the culture for 24 h. To induce polarization of M2-like macrophages, IL-4 (20 μg/ml; PeproTech, Rocky Hill, NJ, USA) and IL-13 (20 μg/ml; PeproTech) were added to the culture for 24 h. On the third day, the Transwells containing the differentiated macrophages were transferred to a six-well plate pre-seeded with NPC cells on an FITC-gelatin coating. For some experiments, THP-1 cells were differentiated and polarized into M1-/M2-like macrophages in a six-well plate. After polarization, the conditioned media were collected to treat NPC cells. The supernatant was centrifuged at 3,000 rpm for 5 min, followed by filtering through a 0.2-μm-pore filter to remove cell debris. The supernatant was used immediately.

*Differentiation and polarization of M1-/M2-like macrophages from human primary monocytes*

Human buffy coat from the Hong Kong Red Cross Blood Transfusion Service was diluted with PBS at a 1:1 ratio. The diluted buffy coat was centrifuged with Ficoll-Paque PLUS density gradient media (GE Healthcare, Waukesha, WI, USA) at a 1:1 ratio at 1,800 rpm for 30 min at 25 °C. The fraction of peripheral blood mononuclear cells (PBMCs) found at the plasma–Ficoll interface was collected and washed three times with PBS. CD14 microbeads (Miltenyi Biotec, Auburn, CA, USA) were used to isolate the monocyte lineage from the PBMCs by magnetic sorting. CD14^+^ monocytes were seeded at a density of 1 × 10^6^ per ml on a Transwell (0.4 μm pore size, 30 mm diameter; Millipore) which was suspended in a six-well plate with 2 ml of medium. To induce differentiation into M1-like macrophages, the cells were first treated with GM-CSF (20 ng/ml; PeproTech) overnight. The next day, LPS (100 ng/ml; Sigma) and IFNγ (20 ng/ml; GenScript) were added to the culture for 24 h. To polarize M2-like macrophages, the cells were first treated with M-CSF (20 ng/ml; PeproTech) overnight. IL-4 (20 μg/ml; PeproTech) and IL-13 (20 μg/ml; PeproTech) were then added to the culture for 24 h. On the third day, Transwells containing polarized M1-/M2-like macrophages were transferred to a six-well plate containing NPC cells pre-seeded on an FITC-gelatin coating. For some experiments, the supernatant of the M1-/M2-polarized macrophages was collected to treat the NPC cells.

*Gelatin coating preparation*

The 15-mm glass coverslips and 35-mm MakTek glass bottom dishes (MatTek Corporation, Ashland, MA, USA) were first coated with 100 or 500 μl of 1 mg/ml FITC-conjugated gelatin (Thermo Fisher Scientific, Waltham, MA, USA), respectively, for 10 min in the dark at room temperature. The FITC-gelatin was aspirated carefully, and glutaraldehyde solution (2% in PBS; Sigma) was added dropwise for 15 min to fix the coating. The coating was then washed three times with PBS. Sodium borohydride (5 mg/ml in PBS; Sigma) was added for 3 min to reduce autofluorescence. Lastly, the coated glass was washed again three times with PBS.

*Flow cytometry analysis*

The polarized M1-/M2-like macrophages were detached and resuspended in PBS. Fc receptor-blocking solution, Human TruStain FcX™ (BioLegend, San Diego, CA, USA), was used to reduce non-specific binding. The macrophages were co-stained with BV605 CD80 antibody (BD Pharmingen, San Diego, CA, USA) and AF647 CD163 antibody (BD Pharmingen) or their corresponding isotype control antibodies (BD Pharmingen) for 30 min at 37 °C in the dark. The stained cells were analyzed using flow cytometry (FACSAria SORP, BD Biosciences, San Diego, CA, USA), and the data were examined using FlowJo (FlowJo, LLC, Ashland, OR, USA).

*Immunofluorescence microscopy*

Cells grown on the gelatin coating were fixed with 4% paraformaldehyde (Merck, Branchburg, NJ, USA) in PBS for 10 min in the dark. The cells were permeabilized with 0.1% Triton X-100 (Sigma) in PBS for 10 min. The samples were blocked with 3% BSA in PBS for 30 min at room temperature. Anti-N-WASP (5F4, 1:100; Novus Biologicals, Littleton, CO, USA), anti-cortactin (4F11, 1:100, Millipore), or SH3PXD2A (18976-1-AP, 1:100; Proteintech, Rosemont, IL, USA) in 3% BSA/PBS were used for primary antibody incubation and their corresponding AF647 secondary antibodies (A21235 and A21245, 1:1,000; Invitrogen, Carlsbad, CA, USA) were used. After washing with PBS, phalloidin AF568 (6.6 μm; Thermo Fisher Scientific) and DAPI (1 µg/ml; Sigma) were added to the cells for 30 min. Images were acquired using confocal laser scanning microscopes with ZEN 2.3 software (LSM800, Carl Zeiss, Oberkochen, Germany). Horizontal plane images (1024 × 1024 pixels corresponding to 253.6 × 253.6 μm) were acquired using a 40× oil objective for each sample. The digested area of gelatin and the total number of cells were measured using ImageJ software. The RGB profiles of invadopodia cross-sections were analyzed with the RGB profiler plugin of ImageJ. A freehand line was plotted across selected invadopodia to analyze the fluorescent signal distributions.

*Inhibition of cell signaling pathways*

Prior to co-culture with TNFα, the cells were treated with various signaling inhibitors including U0126 (MEK inhibitor, 10 μm; Millipore), Src inhibitor-1 (Src inhibitor, 20 μm; Sigma), and erlotinib (EGFR inhibitor, 10 nm; Sigma) for 15 min. TNFα (10 ng/ml; PeproTech) was then added to the culture. For inhibition of N-WASP, 187-1 (10 μm; Millipore) was added to the culture for 24 h. The treated cells were then used for western blot analysis or live-cell imaging.

*Establishing NPC43 and NP460hTert cells stably expressing LMP1*

Plasmids containing PLPCX or PLPCX-LMP1 sequences were transiently transfected into HEK293T/Phoenix cells with VSVG plasmid and X-tremeGENE HP DNA transfection reagent (Roche, Indianapolis, IN, USA) for retrovirus production. The retroviruses were filtered using a 0.45-μm-pore syringe filter and directly added to the culture with polybrene (4 μg/ml). Successfully infected cells were selected using 0.5 μg/ml puromycin. The NPC43 and NP460hTert cells with stable expression of LMP1 were used for studying signaling pathways and the gelatin invasion assay.

*Establishing LifeAct-mCherry-expressing cells*

Plasmid containing the LifeAct-mCherry sequence was a kind gift from Professor Michael Way [56]. LifeAct is a 17-amino acid-long actin-binding peptide. When tagged with a fluorescent molecule, it may be used to visualize filamentous actin (F-actin) structures in live cells. Plasmid containing the LifeAct-mCherry sequence was transiently transfected into HEK293FT cells with psPAX2, pMD2.G plasmid, and X-tremeGENE HP DNA transfection reagent. The lentivirus was filtered using a 0.45-μm-pore syringe filter and directly added to the culture with polybrene (4 μg/ml). Successfully infected cells were selected using 0.5 μg/ml puromycin. A BD FACSAria Fusion Cell Sorter with a 561 nm laser was used to sort the LifeAct-mCherry-expressing cells. Cells expressing LifeAct-mCherry with normal size and granularity were collected.

*Cloning of DNA constructs and mutagenesis*

Q5® Hot Start High-Fidelity DNA Polymerase [New England BioLabs (NEB), Ipswich, MA, USA] was used for cloning. The sequence of the *SRC* gene CDS region was cloned from HEK293T cell cDNA. The wt Src was subcloned into the plasmid pEF1α-IRES-ZsGreen1 between the EcoRI and NheI restriction sites. Src mutants (Y419F, Y530F) were created using a mutagenesis kit (NEB) with desired sequences (supplementary material, Table S1) following the manufacturer’s instructions. The wt and mutant Src sequences were also tagged with a blue-fluorescent marker for confocal imaging by subcloning the sequences into the plasmid EBFP2.N1 (#54595; Addgene, Watertown, MA, USA) between the EcoRI and AgeI restriction sites. The *LMP1* sequence (CDS region) was subcloned into the plasmid EBFP2.C1 (#54665, Addgene) between the EcoRI and BamHI restriction sites. The EBFP2-LMP1 construct was used to visualize the localization of LMP1 during the formation of invadopodia under live-cell imaging. The digested DNA fragments were separated in 1% agarose gels (Biowest, Bradenton, FL, USA) and purified with a DNA purification kit (Thermo Fisher Scientific). The DNA fragments were ligated with T4 ligase (NEB) overnight at room temperature. The plasmids EGFP-Cdc42-wt, -Q61L, and -T17N were obtained from Addgene (#12599, #12600, and #12601). The iRFP670 sequence was obtained from piRFP670-N1 (#45457, Addgene). The plasmids were amplified in competent bacteria, DH5α (Invitrogen), on agar plates with the relevant antibiotics. A single clone was picked for each plasmid and cultured in 1X LB medium. The plasmids were extracted using either the QIAprep Spin Miniprep Kit (Qiagen, Valencia, CA, USA) or the QIAGEN Plasmid Maxi Kit (Qiagen). The sequences used for cloning are listed in supplementary material, Table S1.

*Immunoprecipitation assay*

Culturing medium was replaced with serum-free medium 6 h prior to the collection of the cells. The cells were lysed with ice-cold NP-40 lysis buffer. For the input control, 10 μg of lysate was used, while 400 μg of lysate was incubated with 20 μl of glutathione-*S*-transferase (GST)-Cdc42-binding domain (CBD) of N-WASP binding beads (a kind gift provided by Gareth Jones, King’s College London, UK) on a spinning wheel overnight at 4 °C. Loading buffer (6X, 20 μl) was added to the beads and heated to 95 °C for 10 min for protein denaturation. The samples were then validated using western blotting.

## *Western blotting and analysis*

Cells were first rinsed with ice-cold PBS on ice, then lysed with RIPA buffer [10 mm Tris–HCl, pH 8.0, 1 mm EDTA, 0.5 mm EGTA, 1% Triton X-100, 0.1% sodium deoxycholate, 0.1% SDS, 140 mm NaCl, and phosphatase inhibitor (PhosSTOP™; Roche, USA)], and collected in 1.5-ml tubes using a cell scraper. The samples were centrifuged at 14,000 × *g* for 10 min at 4 °C. The supernatant was collected, and the protein concentration was quantified using the Bio-Rad DC protein assay kit (Bio-Rad, Hercules, CA, USA). The protein concentrations in all samples were adjusted; SDS-containing loading buffer was added to the samples; and the samples were boiled at 95 °C for 10 min.

Protein samples (10–20 μg) were loaded into an SDS-PAGE gel electrophoresis system (Mini-Protean II Electrophoresis Cell, Bio-Rad) containing running buffer. The voltage was set to 110 V, and the system was run until the loading dye reached the bottom of the gel. The proteins in the gel were transferred to a polyvinylidene difluoride (PVDF) membrane for 2 h. The PVDF membrane was then blocked with 5 g/100 ml skimmed milk powder in TBST for 30 min. The primary antibodies (supplementary material, Table S1) in 5 g/100 ml skimmed milk powder in TBST were applied to the membrane, and the membrane was incubated overnight at 4 °C. The next day, the membrane was washed three times with TBST for 10 min each time. Secondary antibodies in 5 g/100 ml skimmed milk powder in TBST were then applied to the membrane, followed by incubation for 1 h at room temperature. The membrane was then washed three times with TBST for 15 min each time and probed with chemiluminescence (Bio-Rad). The fluorescent signal was captured on an X-ray film. The antibodies used for western blotting are listed in supplementary material, Table S1.

*Immunohistochemistry*

Formalin-fixed, paraffin-embedded (FFPE) primary NPC sections (4 μm) were deparaffinized and rehydrated. The slides were placed in 100 °C sodium citrate buffer (10 mm sodium citrate, 0.05% Tween 20, pH 6.0) for 15 min for antigen retrieval. The endogenous peroxidase and biotin activity were blocked by hydrogen peroxide solution and 3% BSA solution in PBS, and the sections were incubated in primary antibody (anti-TNFα; Santa Cruz Technology, Dallas, TX, USA) in a moist chamber. The HRP-labeled secondary antibody was applied to the sections for 1 h and then the DAB substrate was applied for color development. The slides were counterstained with hematoxylin for nucleus visualization. Lastly, the slides were dehydrated and mounted with Permount mounting medium (Thermo Fisher Scientific).

*Bioinformatic analysis of RNA-Seq data*

NPC43^EBV−ve^ and NPC43^EBV+ve^ cells with or without TNFα treatment were lysed in 1 ml of TRIzol. The lysates were sent to a company (Genewiz, Suzhou, PR China) for library preparation, sequencing, and data analysis. In brief, the libraries were prepared using the Illumina TruSeq mRNA Library Prep Kit (Illumina, San Diego, CA, USA) according to the manufacturer’s instructions. Sequencing was performed on an Illumina HiSeq2000 sequencing system. The gene expression ratios were calculated based on the fragments per kilobase million (FPKM) values for each gene. The threshold for differentially expressed genes was set as log2(fold-change) > 1.3 and log2(fold-change) < 0.7. The heatmap was generated using ClustVis for visualizing and clustering multivariate data [60].

**Supplementary Figures S1–S6**

**
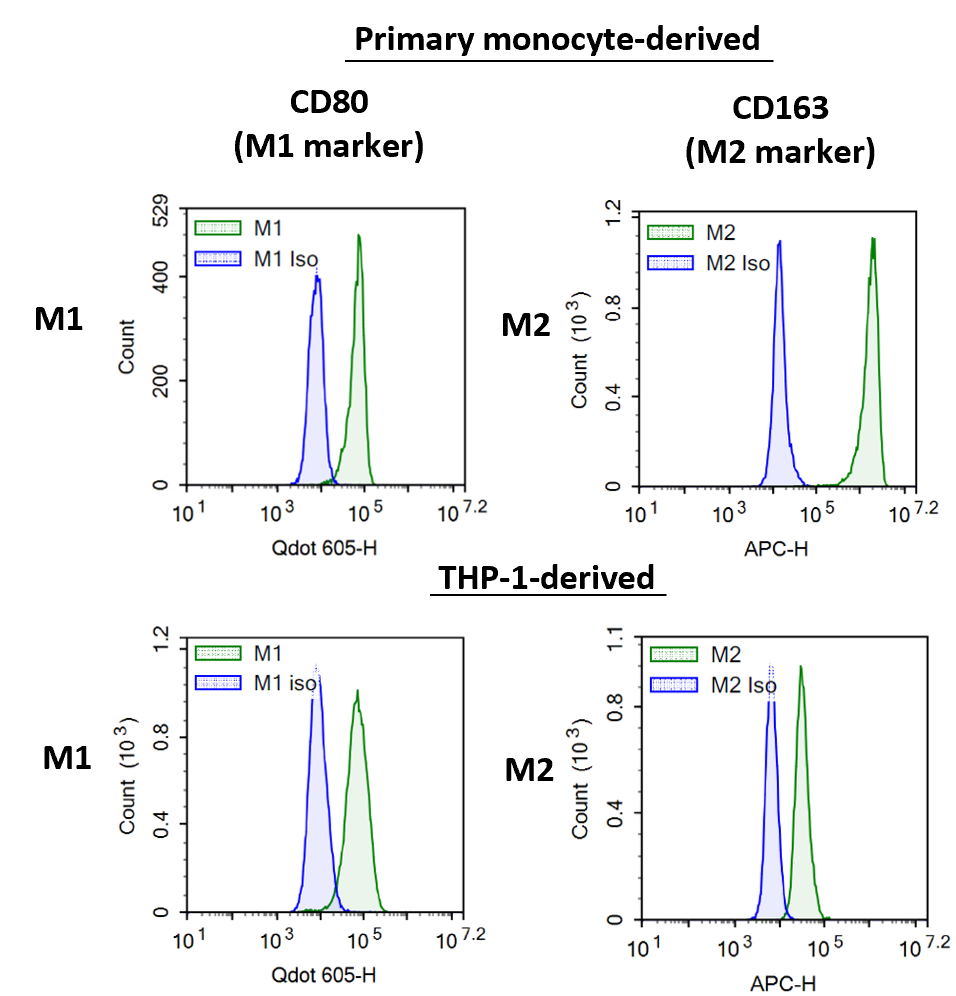
**

**B**


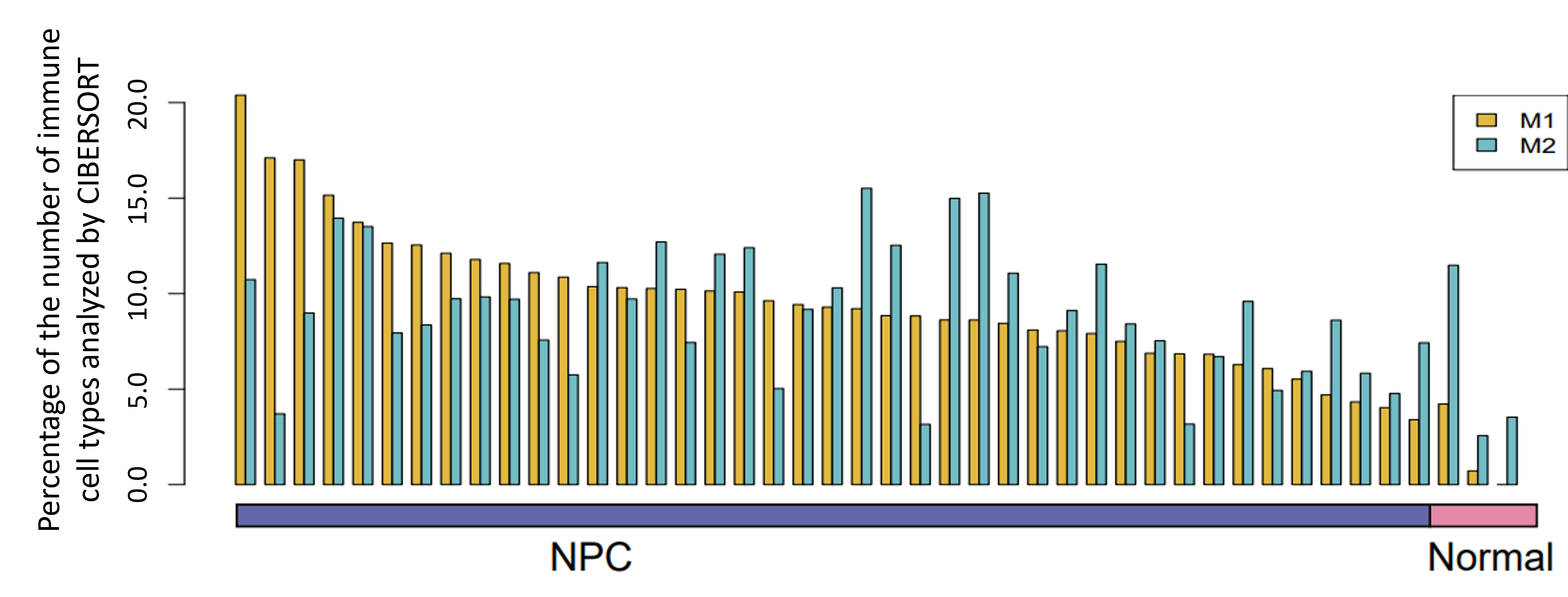
**A**


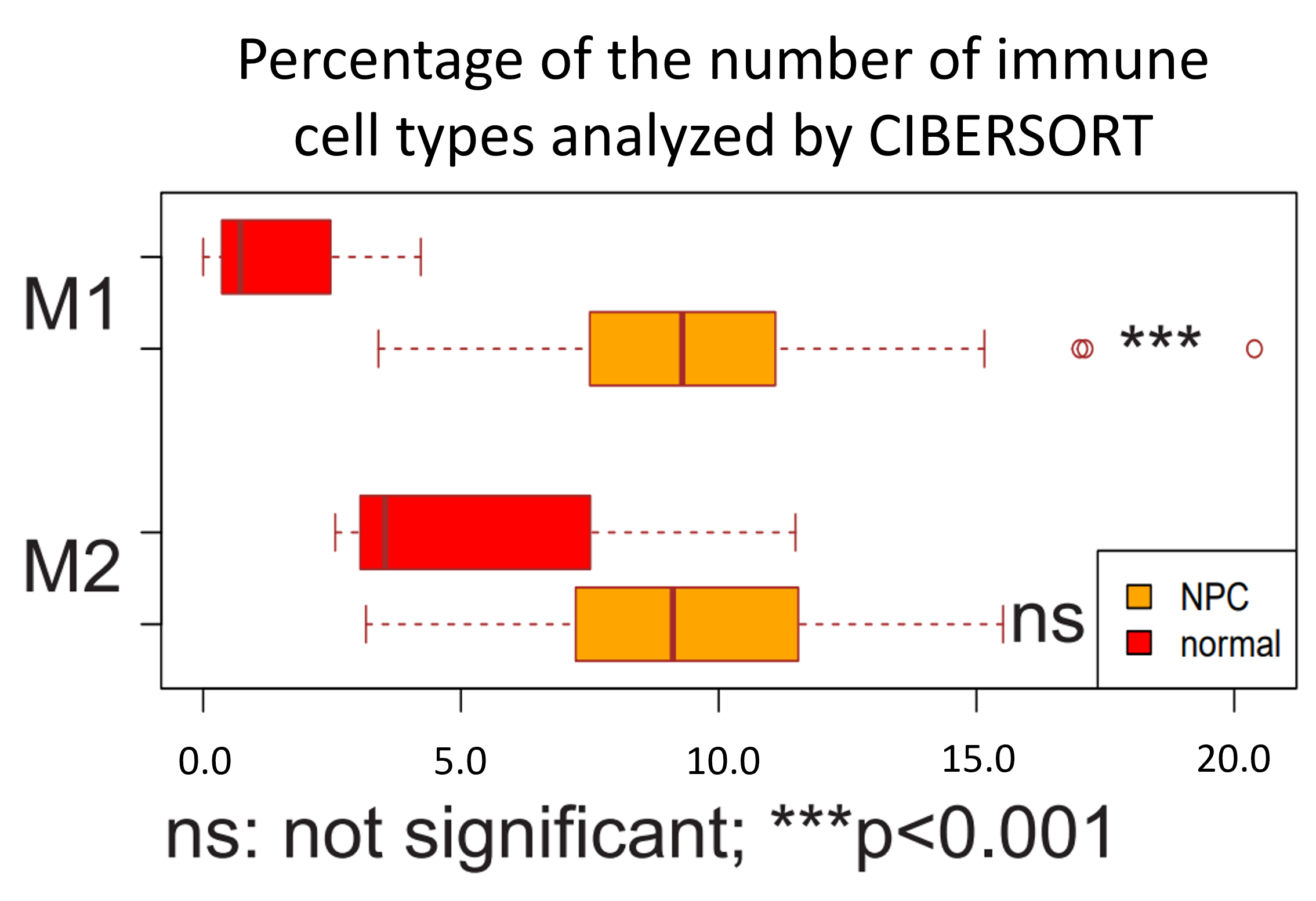


**C**

**
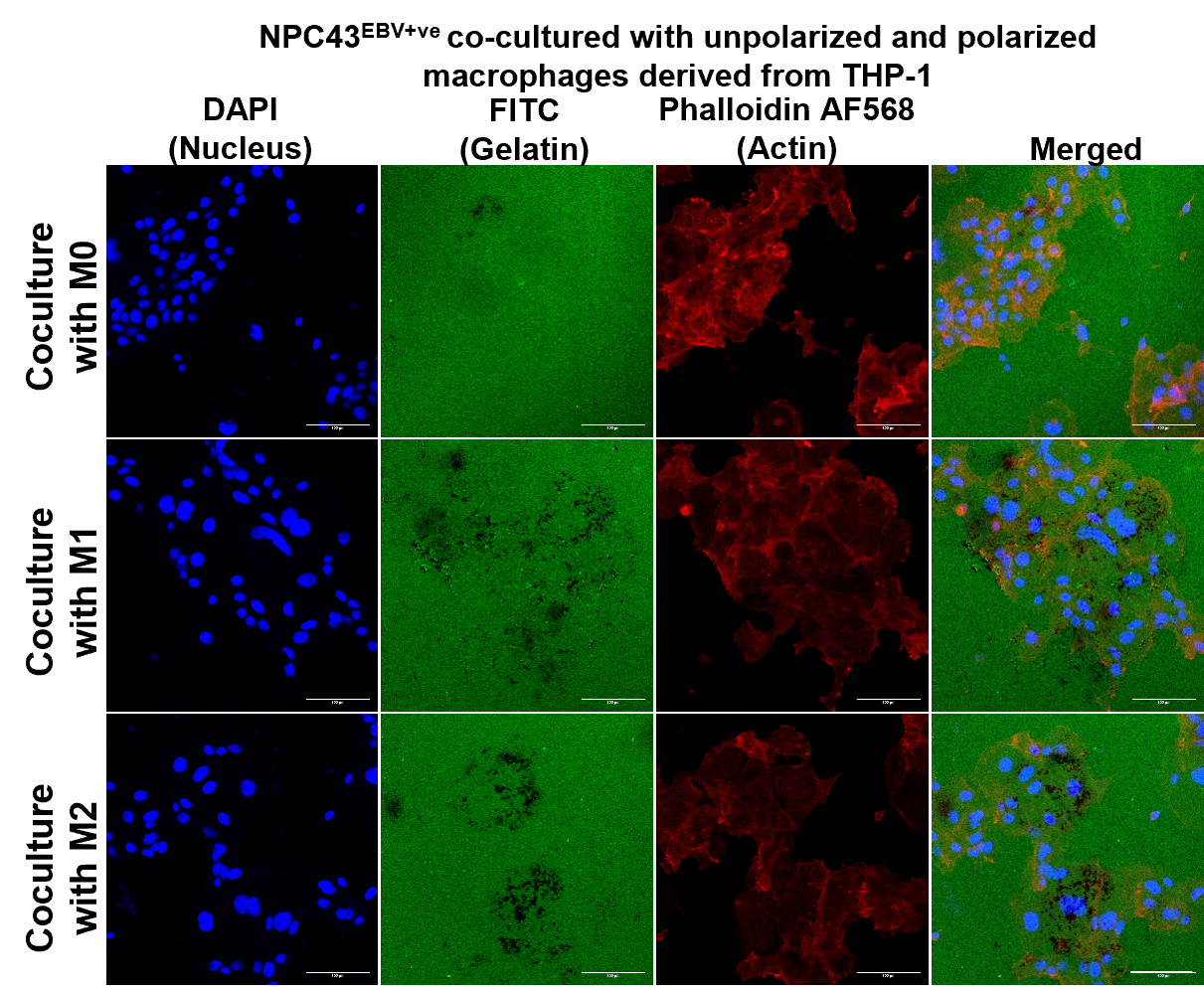

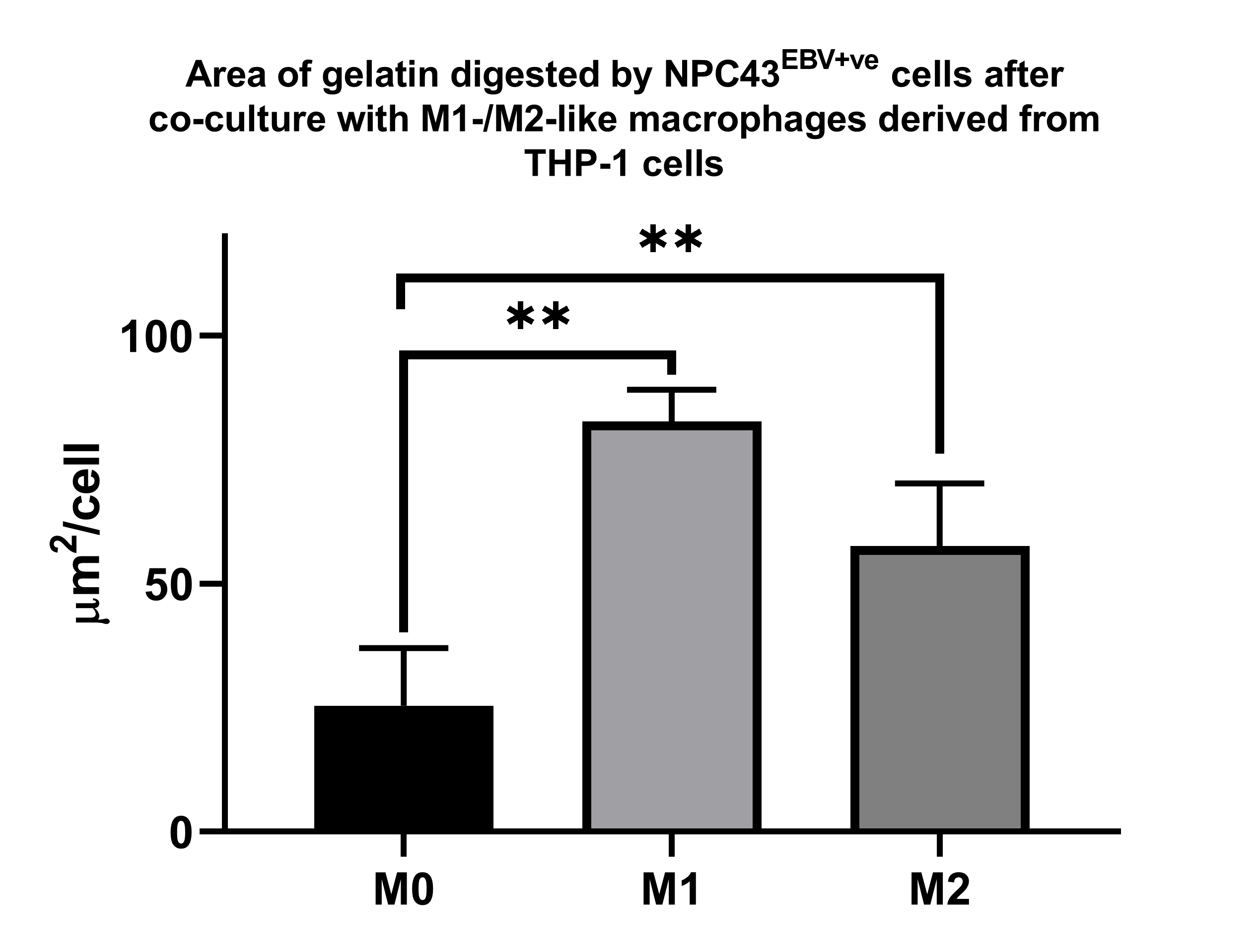
**

**
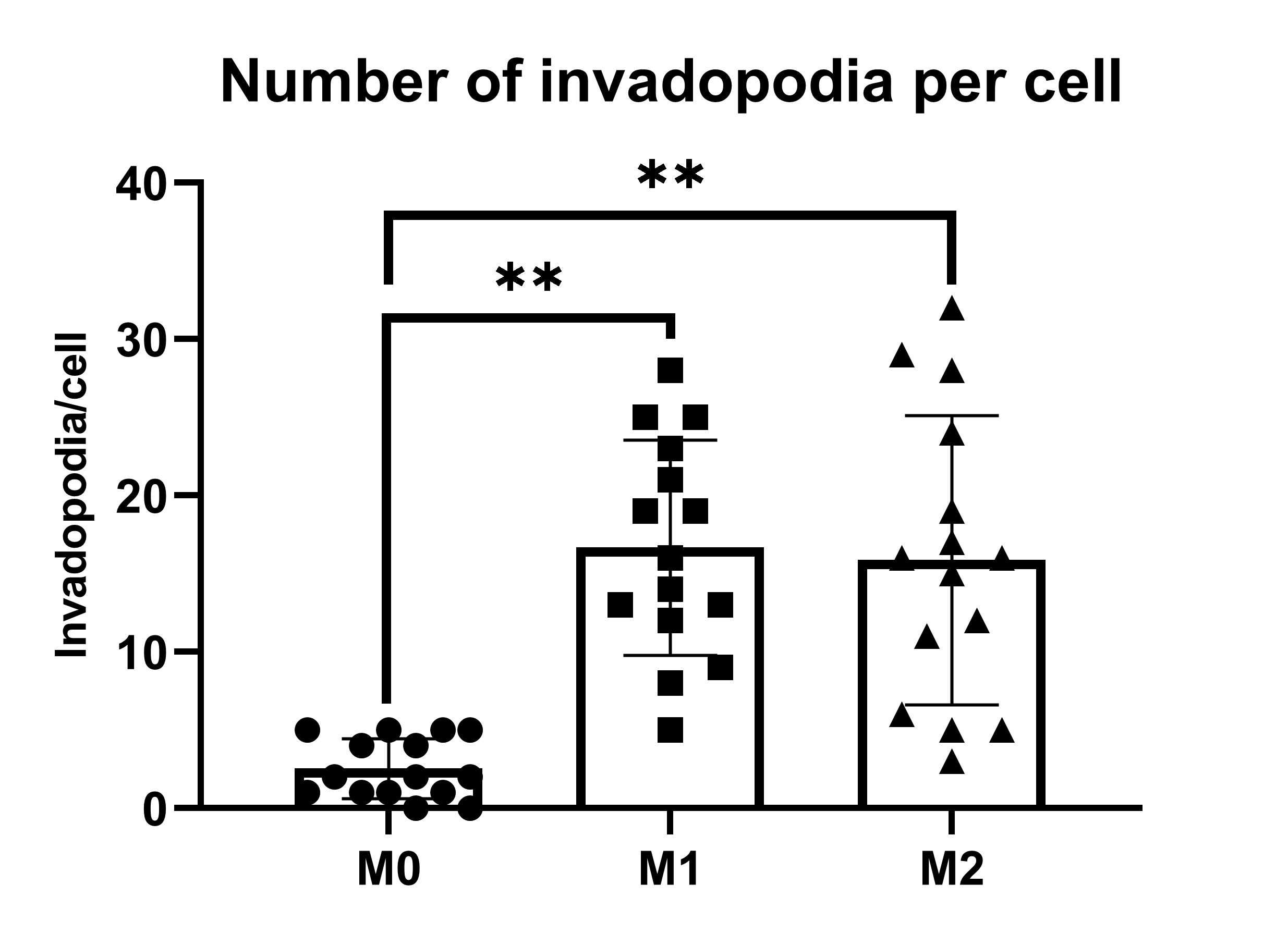
**

**Figure S1.** **Polarized M1-/M2-like macrophages induced invadopodia information in NPC43^EBV+ve^ cells. Related to Figure 1.** (A) Top: percentages of M1- and M2-like macrophages estimated from the bulk RNA sequencing data of NPC tumors (*n* = 41) and healthy tissue (*n* = 3) by CIBERSORT. The data were obtained from the Gene Expression Omnibus (GEO) (Accession No. GSE69799) and the TPM (transcript per million) was used for deconvolution using the default parameter in CIBERSORT. Bottom: a comparison of the percentages of M1- and M2-like macrophages out of total immune cells was made between NPC tumors (*n* = 41) and non-tumor tissue (*n* = 3). ****p* < 0.001; ns: not significant; Mann–Whitney *U*-test. (B) Validation of polarized M1-/M2-like macrophages derived from primary monocytes or THP-1 using flow cytometry. M1 (CD80) and M2 (CD163) markers were used to confirm successful polarization. (C) Left: confocal images of FITC-gelatin digested by NPC43^EBV+ve^ cells after co-culture with polarized M1-/M2-like macrophages derived from THP-1 cells. Scale bar: 100 µm. Right: statistical analysis of the number of invadopodia formed and the area of FITC-gelatin digested per NPC43^EBV+ve^ cell. Means ± SEM. Student’s *t*-test *P* value indicated the significant difference among the compared groups (***p* < 0.01). Each of the above experiments was repeated three times (*N* = 3).


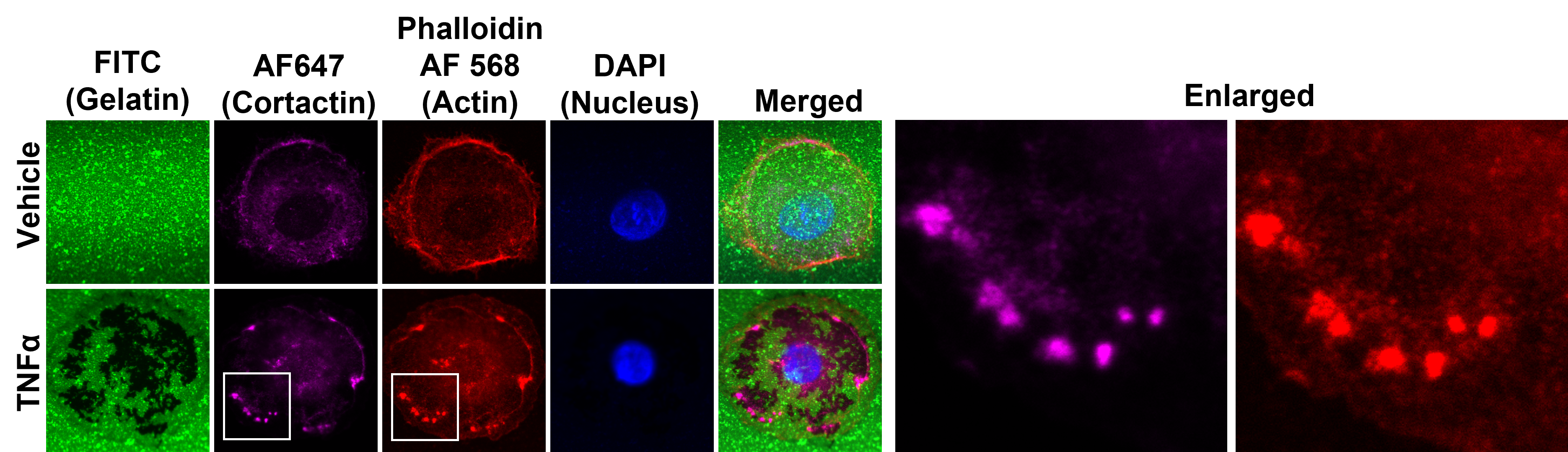
**A**

**
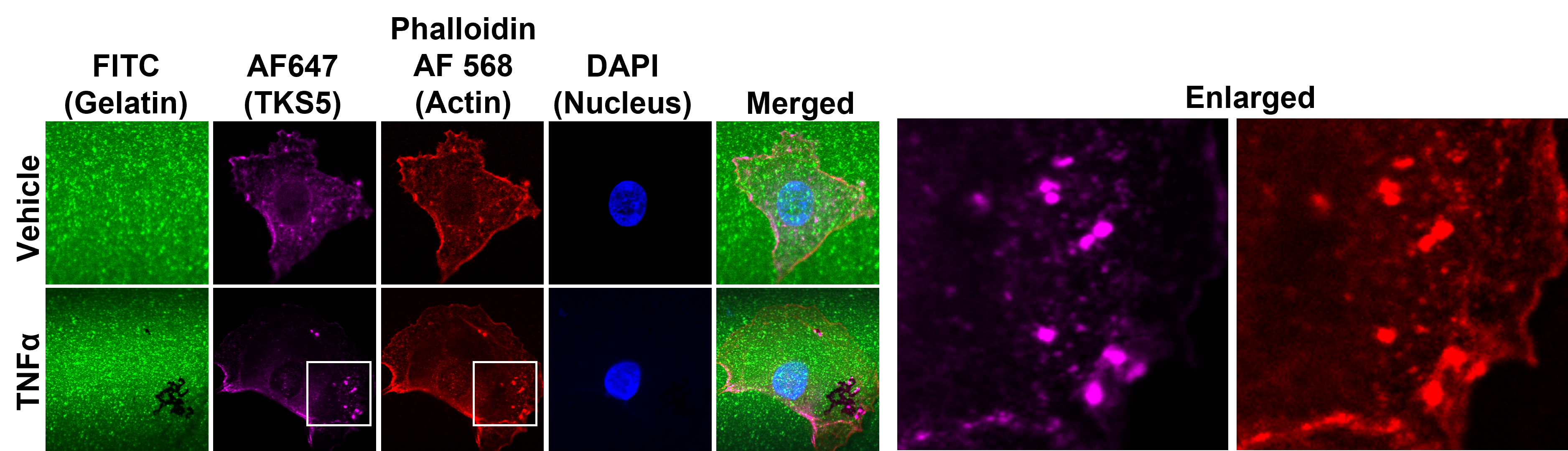
**


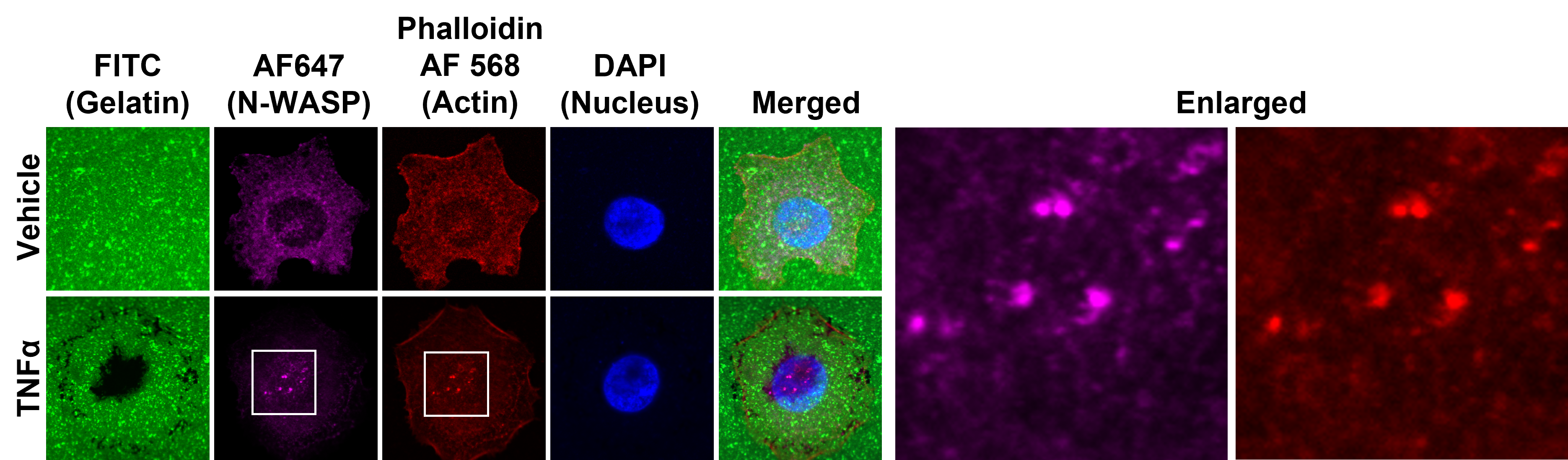


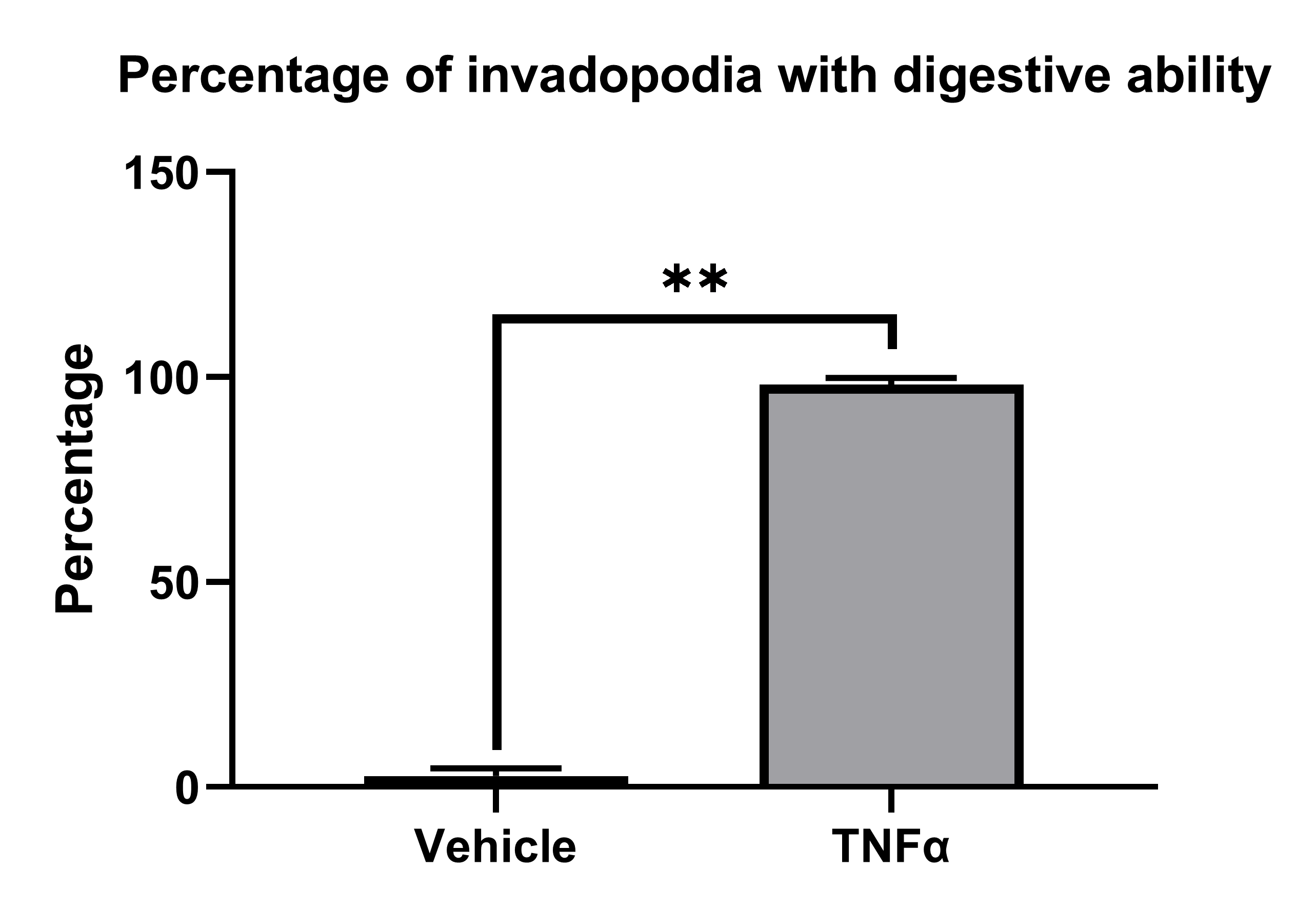

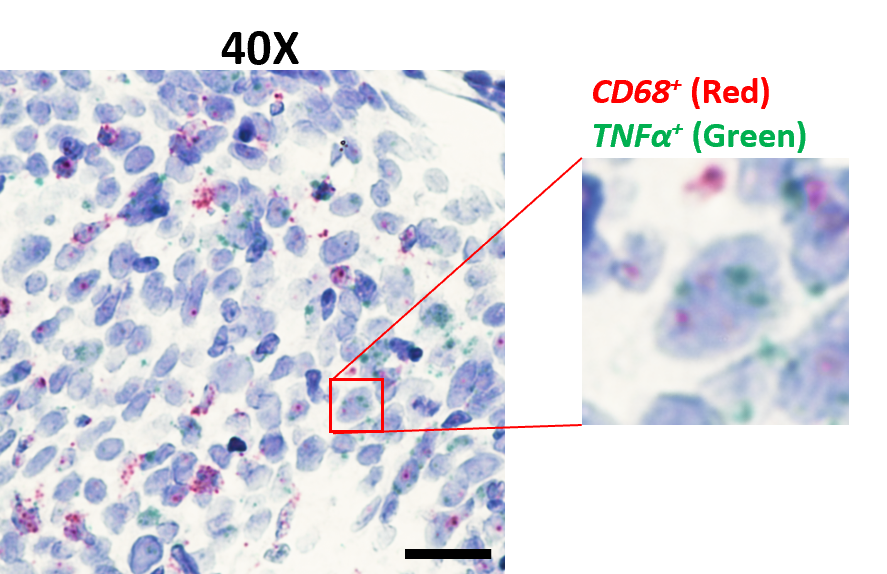
**B D**

**C**

**
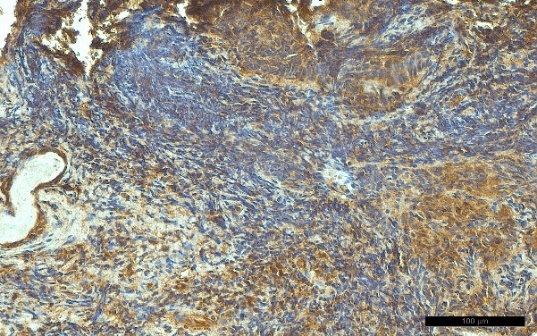

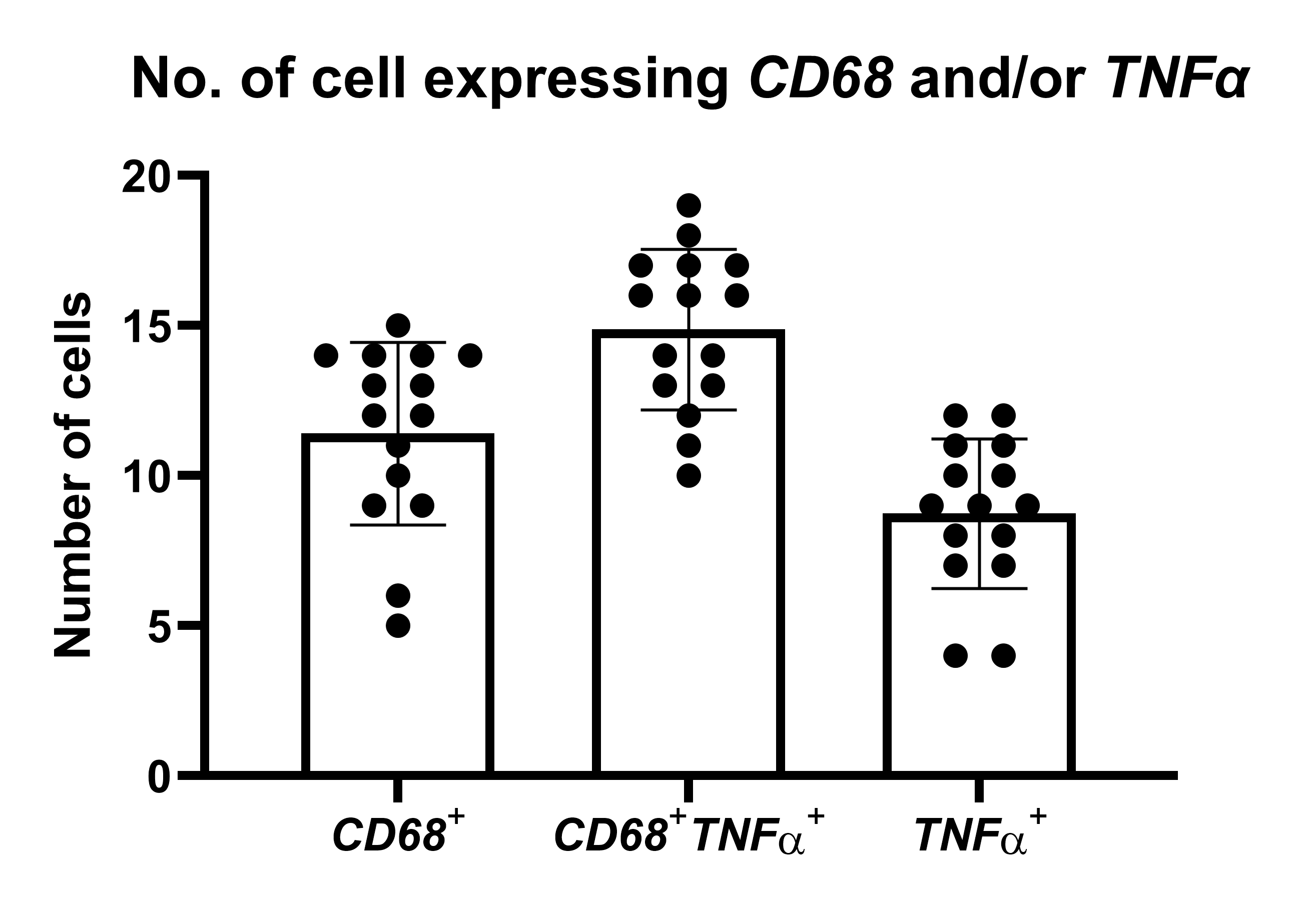
**

**Figure S2. Macrophage-derived TNFα could induce invadopodia information in NPC43^EBV+ve^ cells. Related to Figure 2.** (A) Left: co-localization of cortactin, TKS5, and N-WASP at the invadopodia (punctate dots of actin) in NPC43^EBV+ve^ cells under TNFα treatment. Right: magnified images of the markers and invadopodia from the white box are presented. (B) Statistical analysis of the percentage of invadopodia with digestive ability. Approximately 98% of invadopodia induced by TNFα possessed digestive capabilities (related to Figure 2B). Means ± SEM. Double asterisks denote values significantly different from control cells (*p* < 0.01). (C) Representative image showing the diffuse IHC staining of TNFα in NPC tissue. Scale bar: 100 μm. *N*= 8. (D) RNAscope analysis indicating that a portion of *CD68*^+^ cells (red) express the transcripts of *TNF* (green). Scale bar: 40 μm. *N* = 8. Means ± SEM. Student’s *t*-test *P* value indicated the significant difference among the compared groups (***p* < 0.01). Each of the above experiments was repeated three times (*N* = 3).


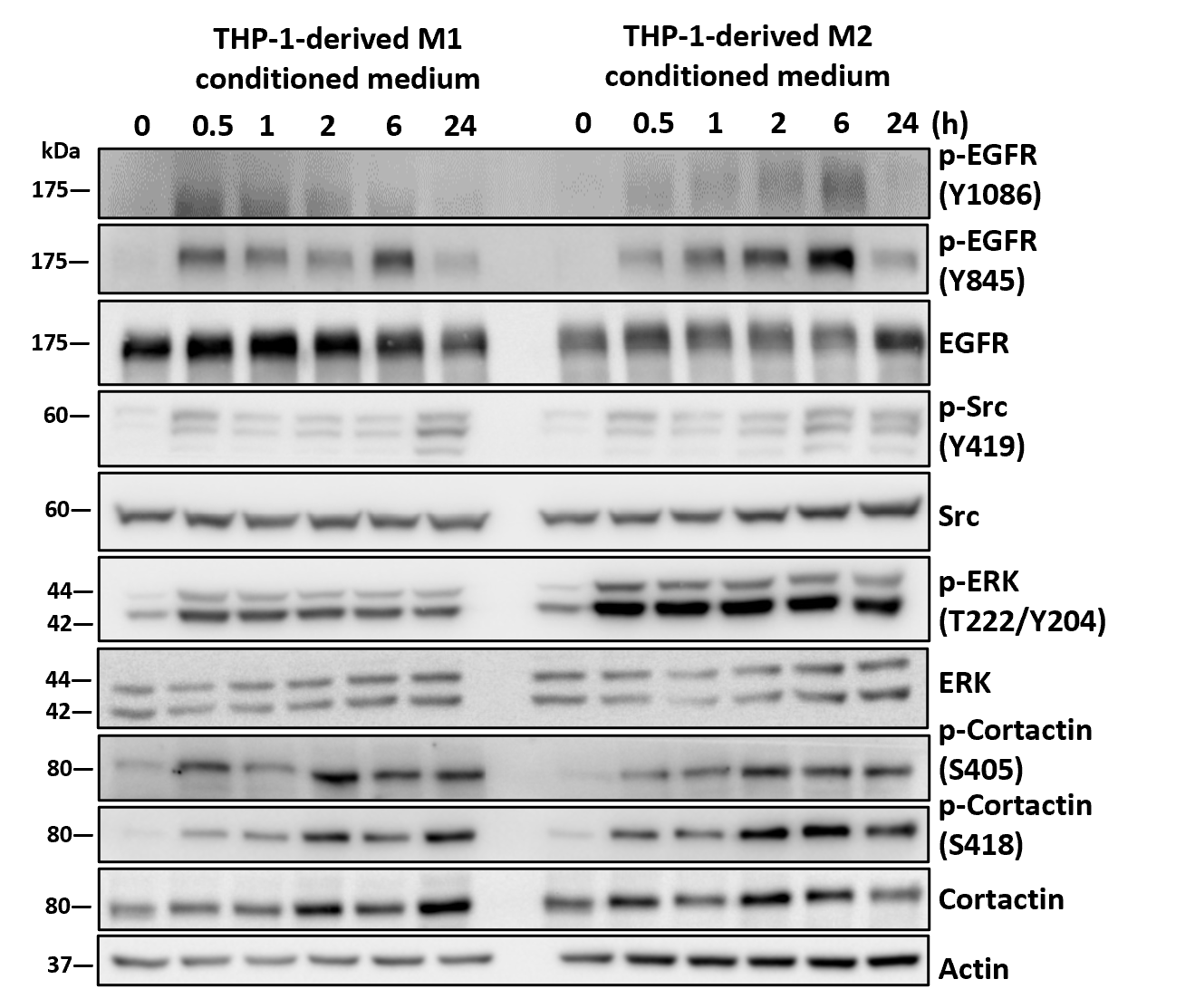
**A B**

**
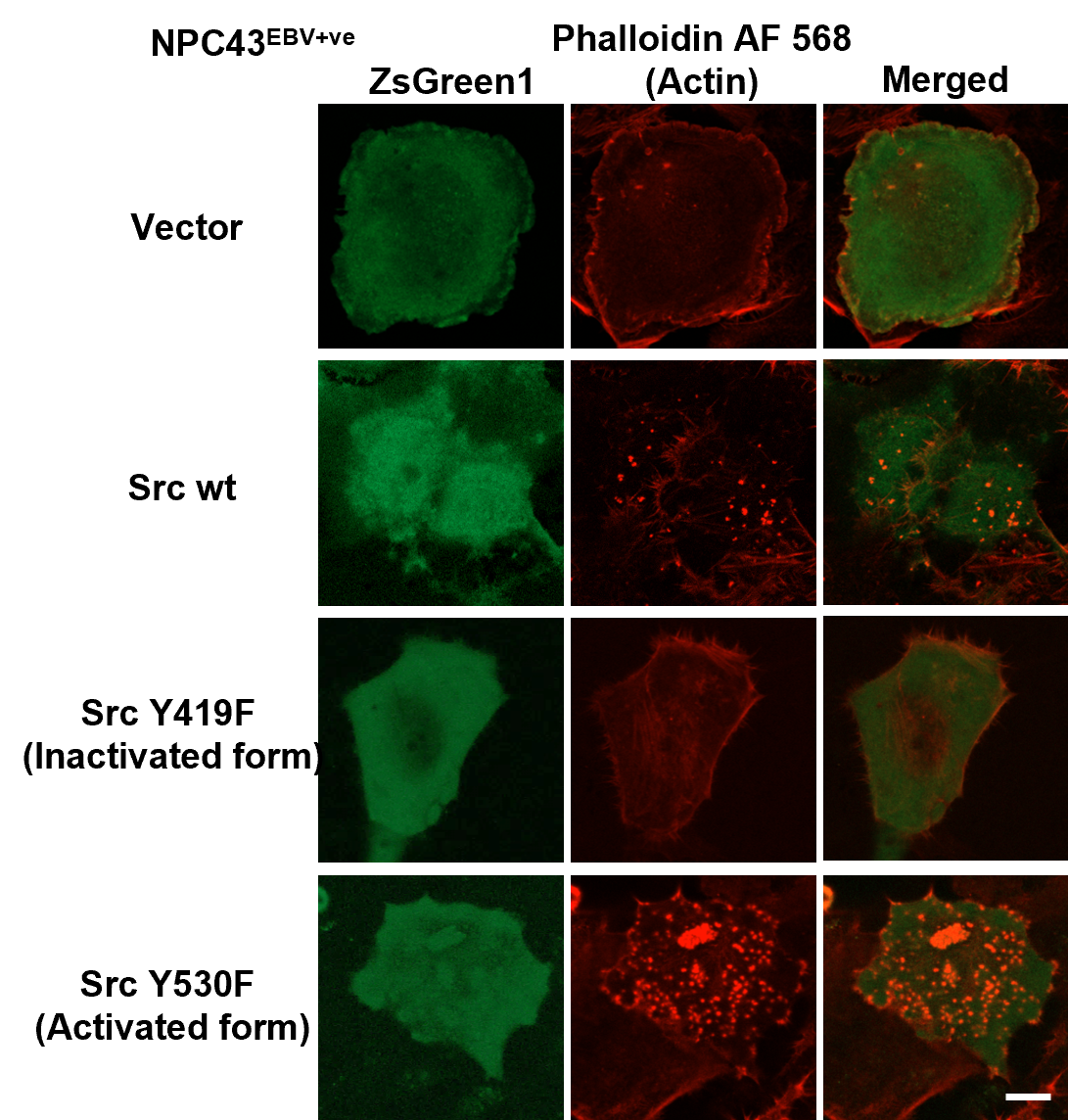
**

**
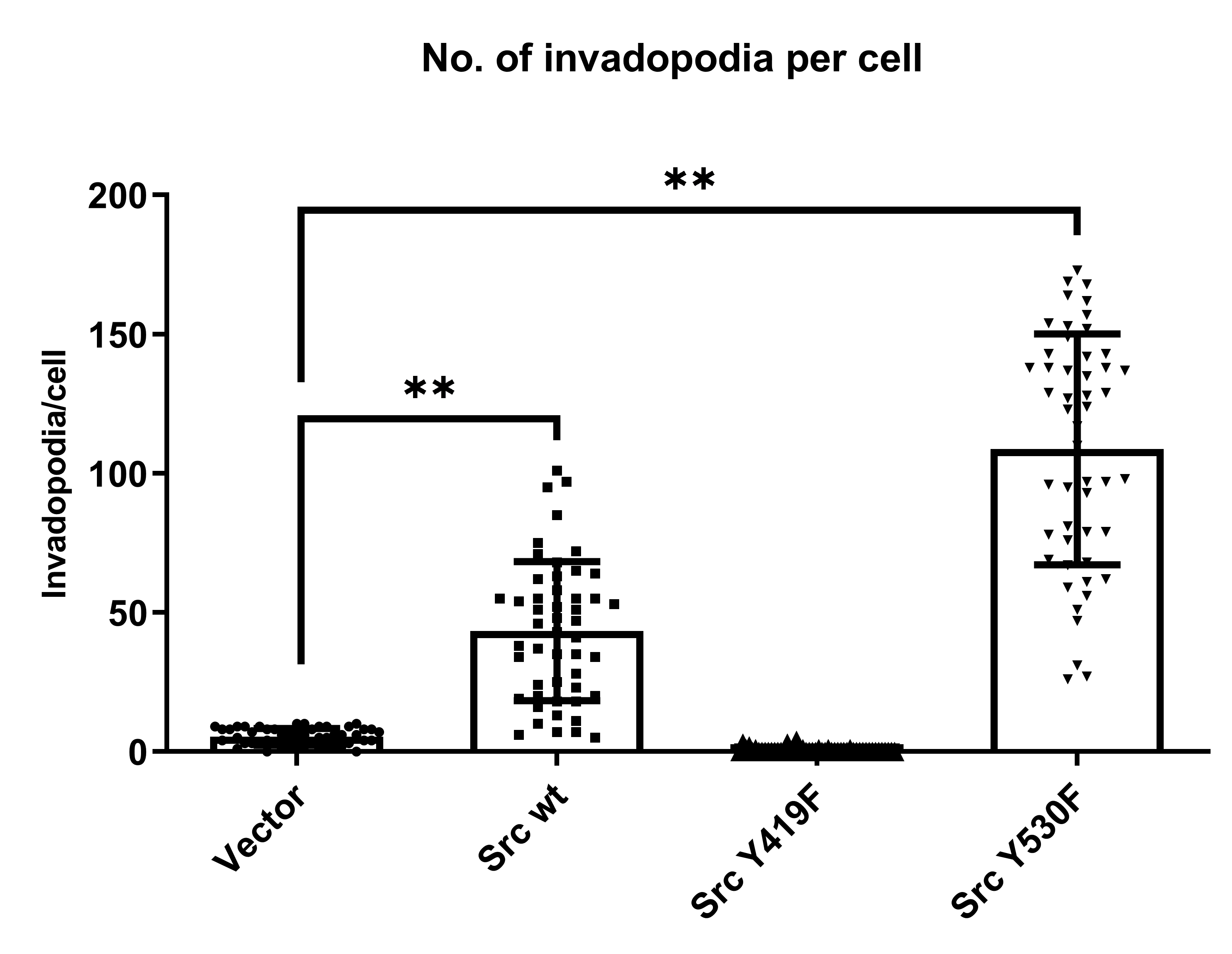
**


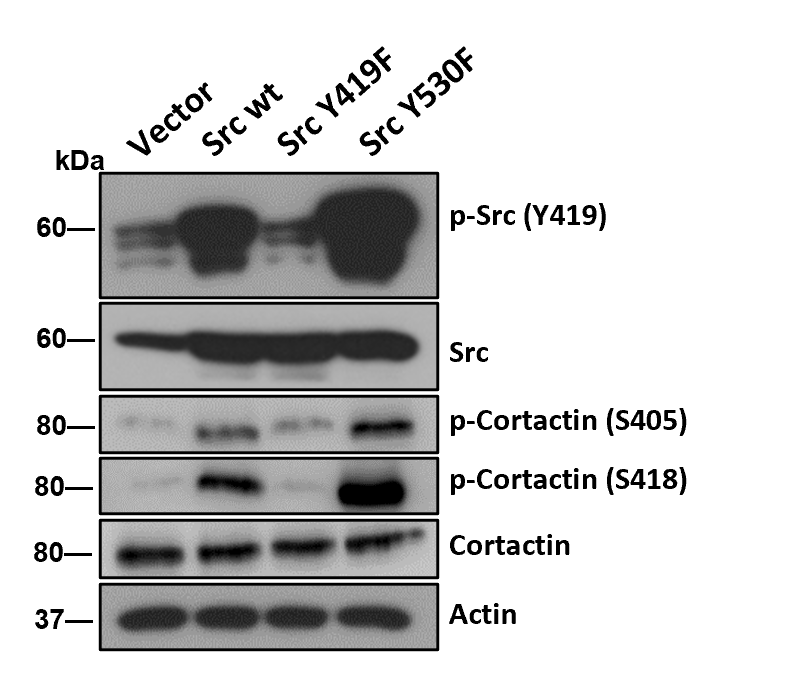
**C**

**
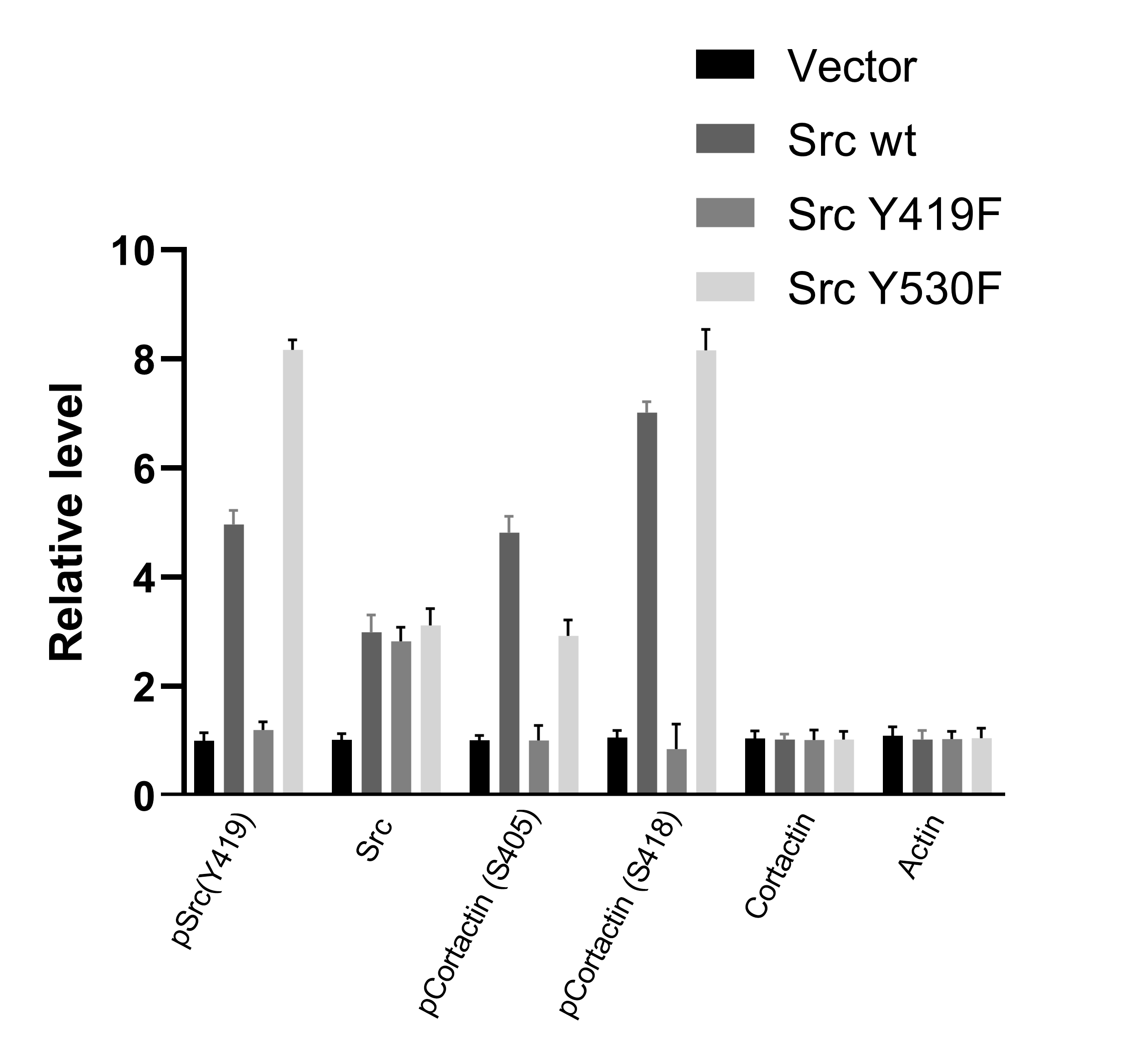
**


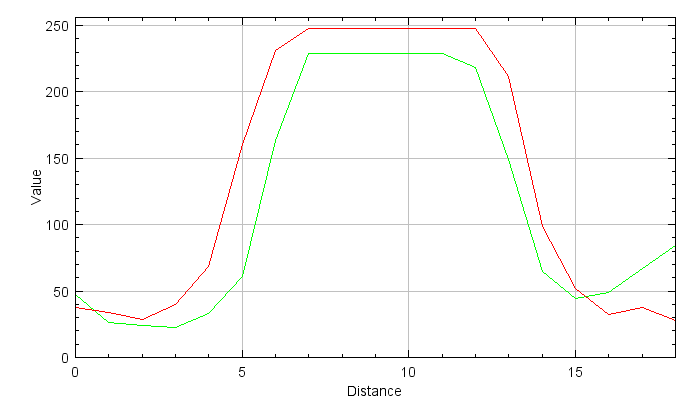

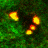

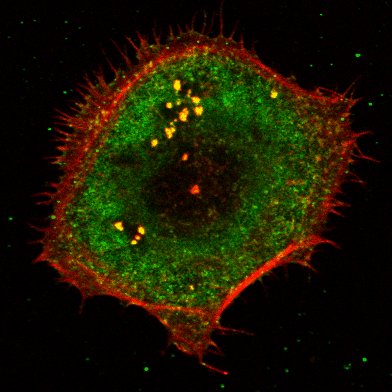


**Red: Phalloidin AF 568 (Actin)**

**Green: AF 488 (Cortactin)**

**D**

**
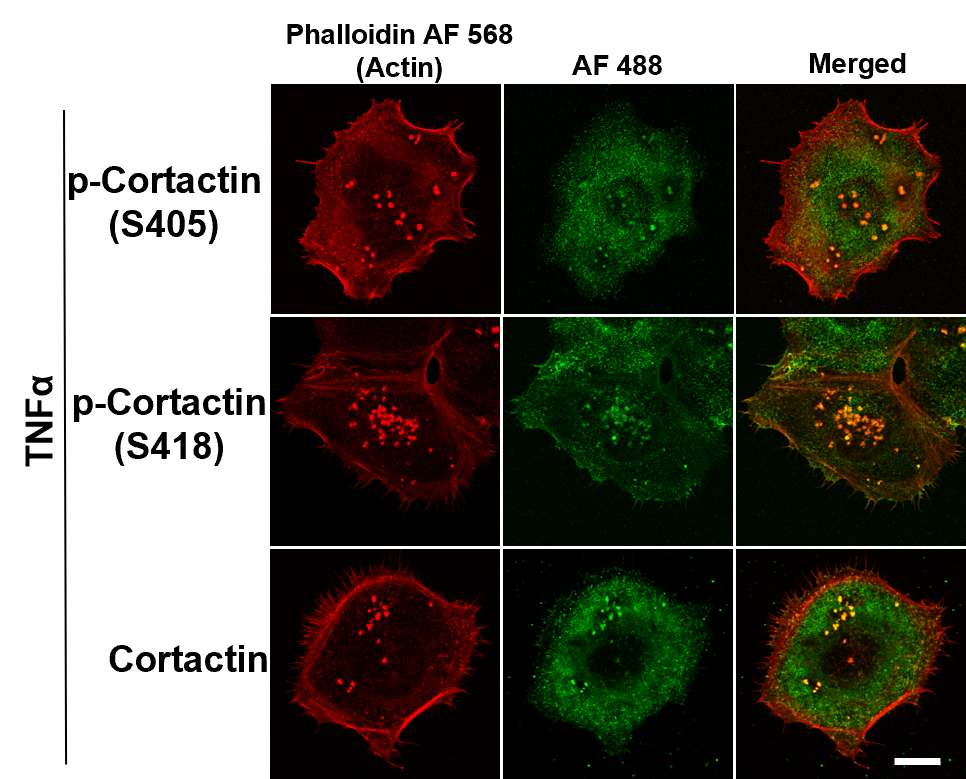
**


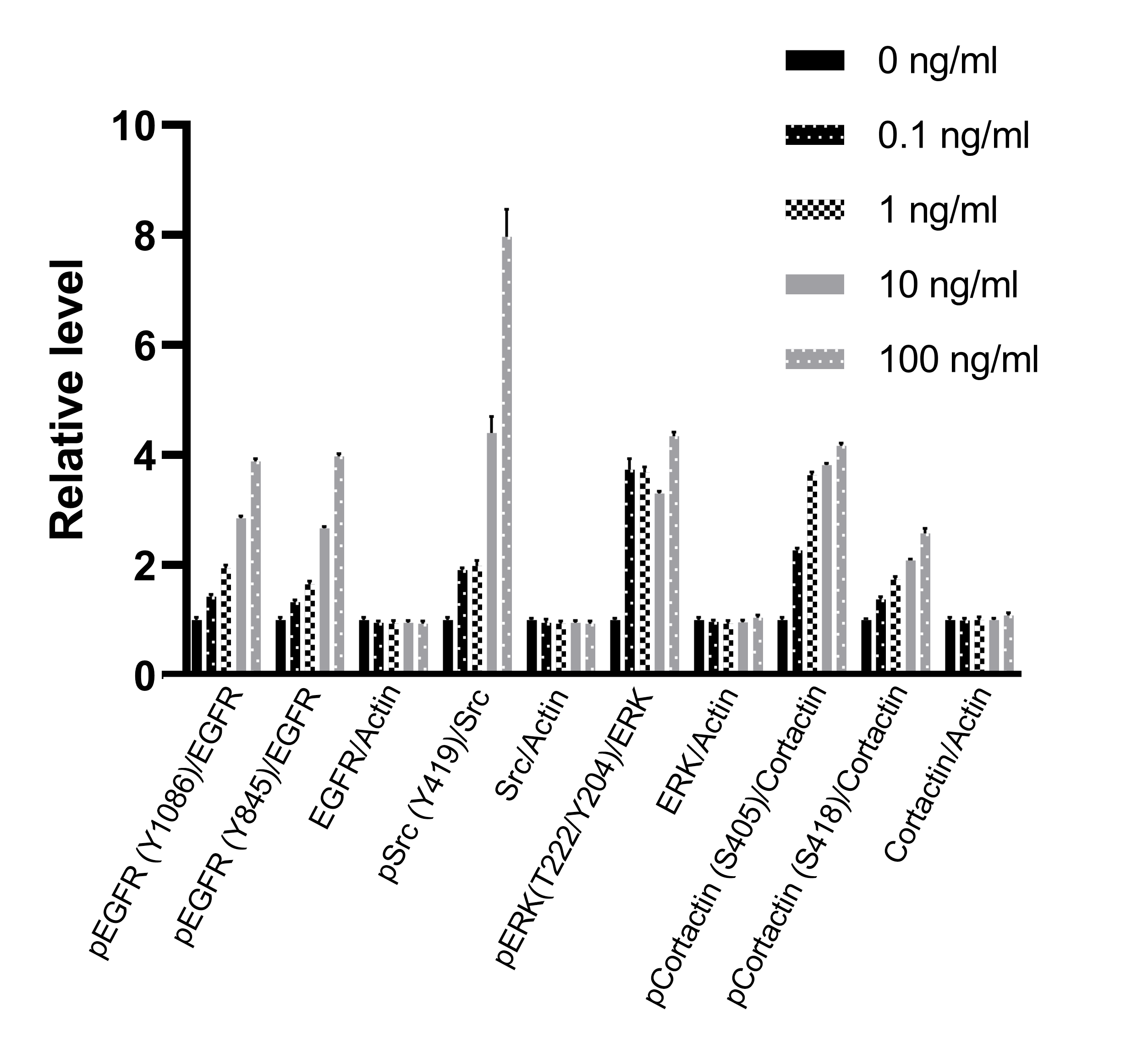

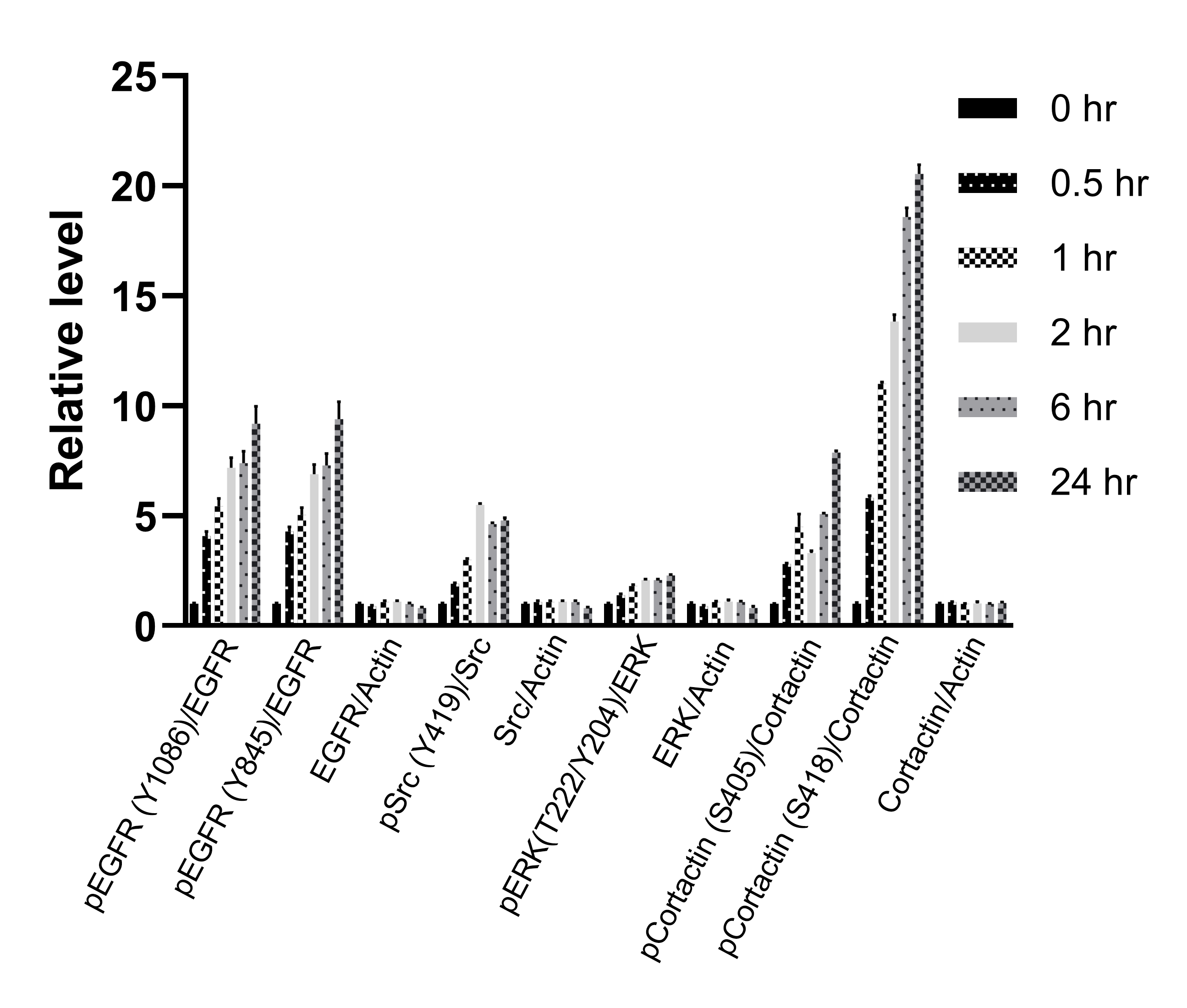
**E F**

**
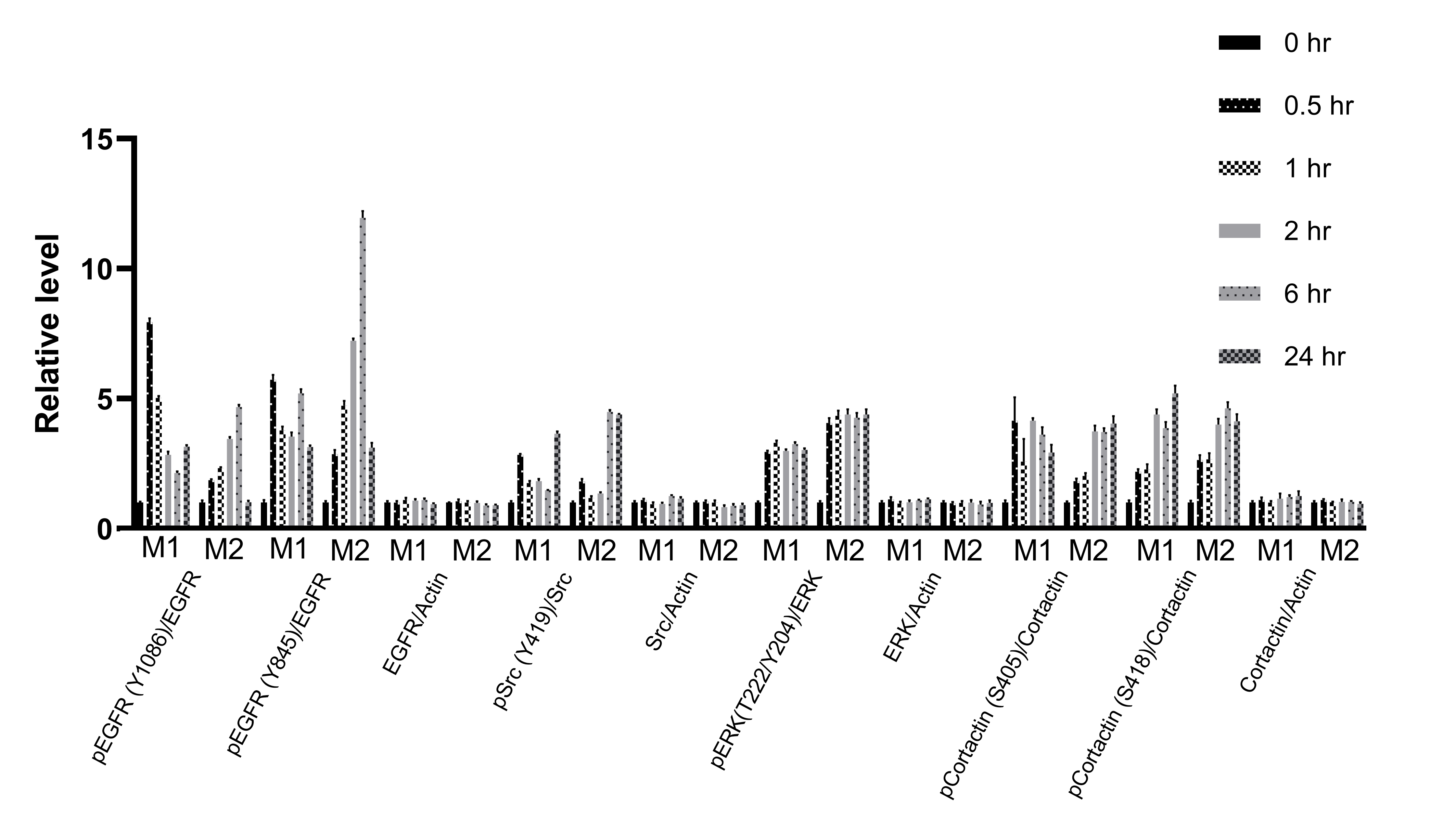
**

**G**


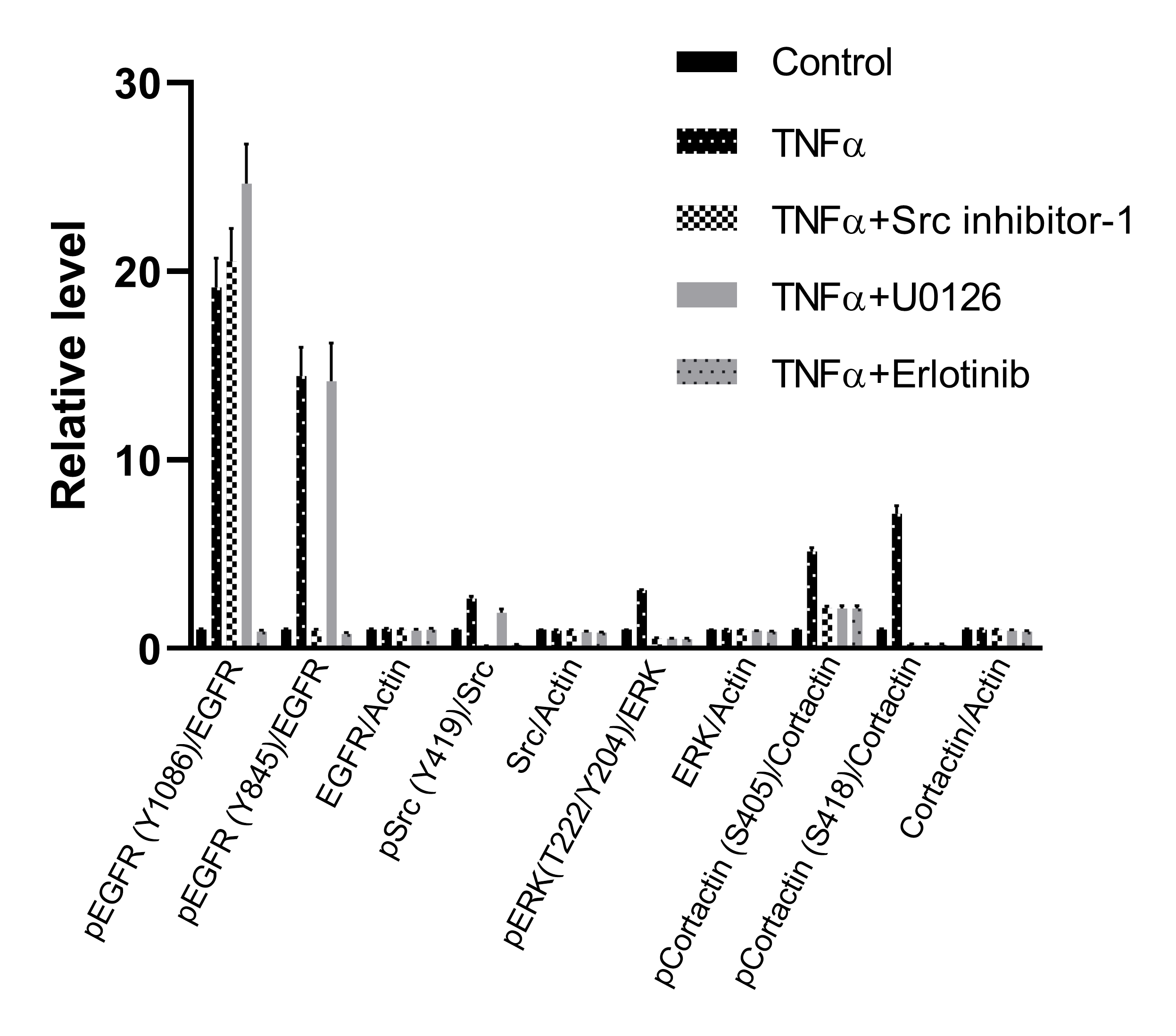
**H**

**
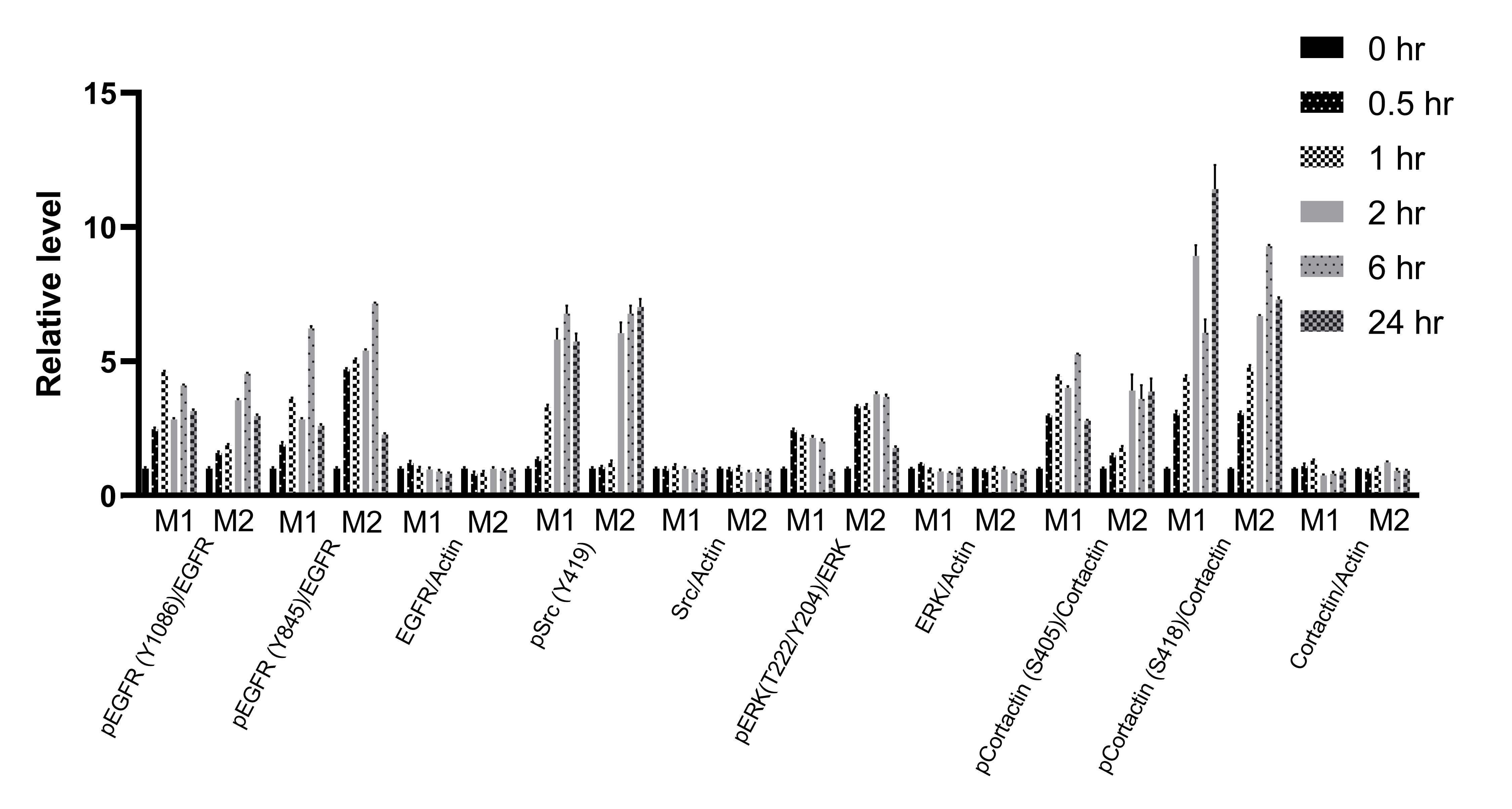
**

**I**

**Figure S3.** **TNFα, EGFR, Src, ERK, and cortactin are essential for invadopodia formation. Related to Figure 3.** (A) Western blot analysis of EGFR, SRC, ERK, and cortactin phosphorylation in NPC43^EBV+ve^ cells treated with THP-1-derived M1-/M2-like macrophage conditioned media at different time points. (B) Top: confocal images of NPC43^EBV+ve^ cells transiently transfected with bicistronic plasmids containing the sequence for wild-type, inactivated (Y419F), or activated (Y530F) Src. ZsGreen1 expression indicated successful transfection. Scale bar:  5 µm. Bottom: statistical analysis of the number of invadopodia per cell induced by SRC wt and its mutants. (C) Western blot analysis illustrating upregulated phosphorylation of SRC and cortactin in NPC43^EBV+ve^ cells after transfection with the wild-type and activated SRC. (D) Left: presence of cortactin at the invadopodia. Phospho-cortactin is present within the actin core of invadopodia in TNFα-treated NPC43^EBV+ve^ cells. Scale bar: 5 µm. Right: a magnified image was used for analysis. An RGB profile shows the cross-section (white line) of an invadopodium. Scale bar:  0.1 µm. Means ± SEM. Double asterisks denote values significantly different from control cells (*p* < 0.01). (E–H) Quantification of protein expression in the western blots of Figure 3A–D. (I) Quantification of protein expression in the western blot of supplementary material, Figure S3A. The values reported under each blot (E–I) are the mean fold of protein expression relative to vehicle controls taken as 1 after normalization by the non-phosphorylated form of the corresponding protein or actin (ImageJ quantification). Means ± SEM. Student’s *t*-test *P* value indicated the significant difference among the compared groups (***p* < 0.01). Each of the above experiments was repeated three times (*N* = 3).


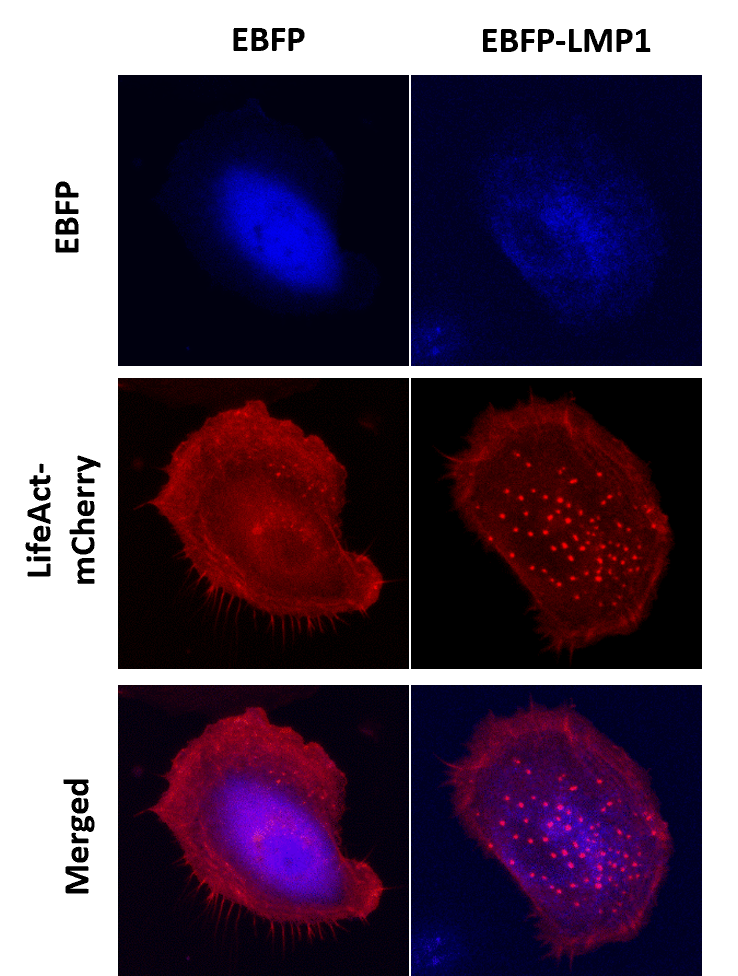
**A B**

**
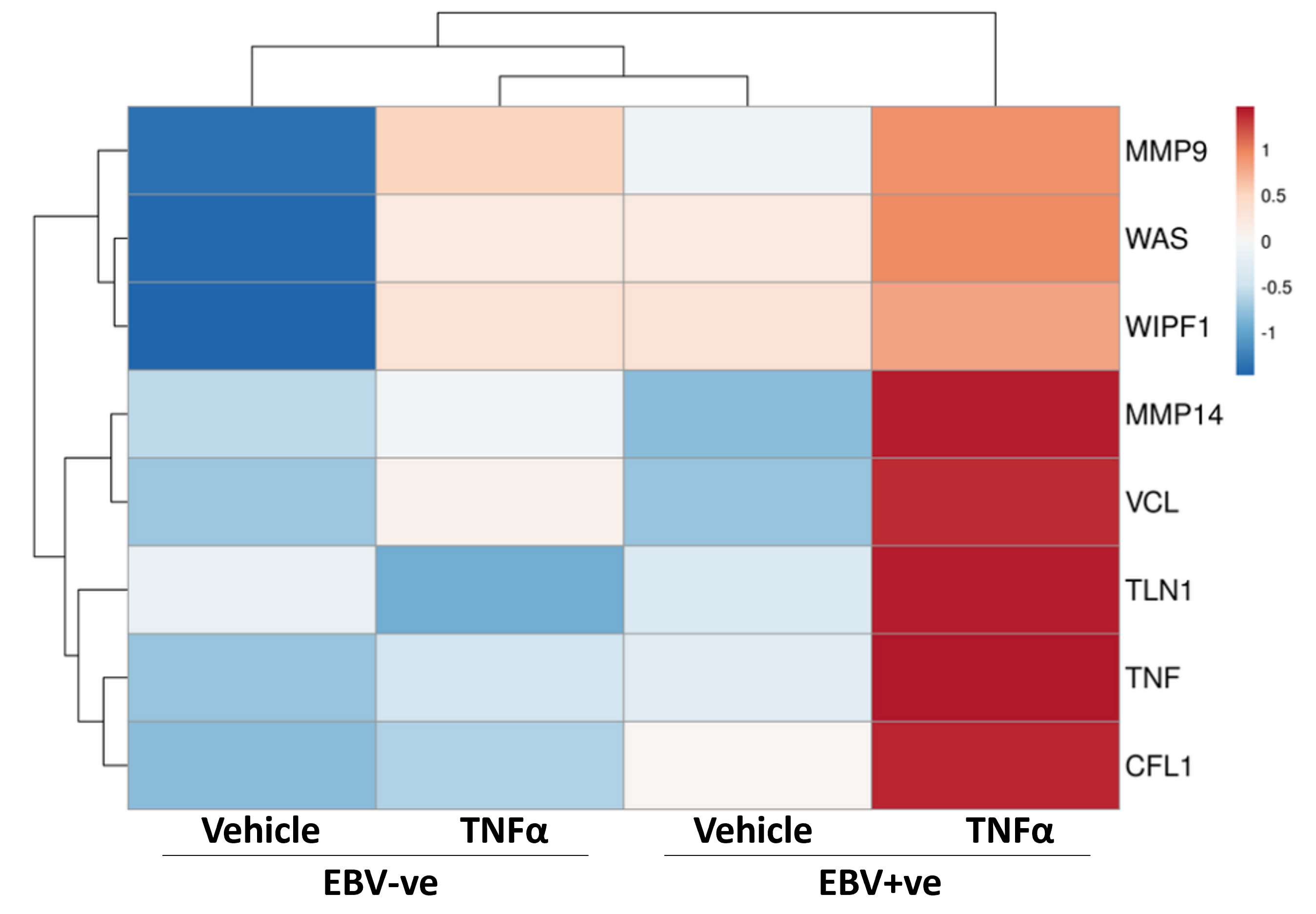
**


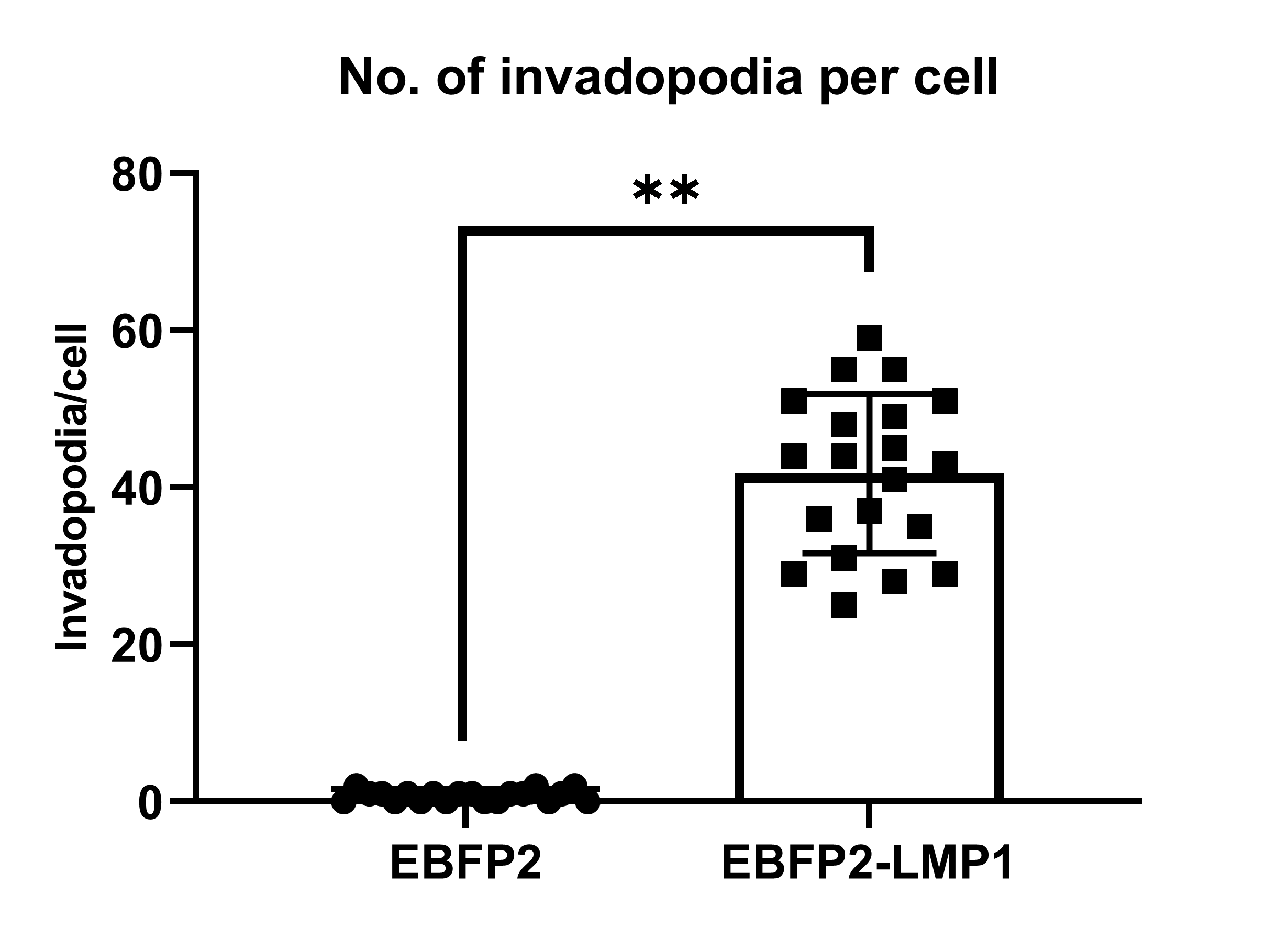


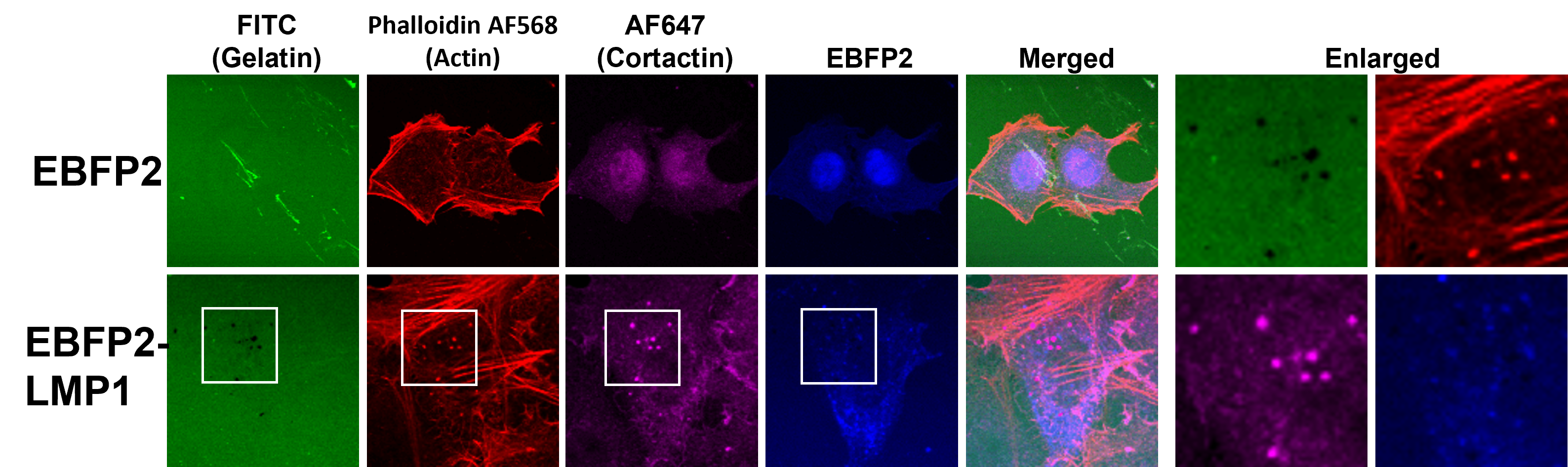
**C**

**
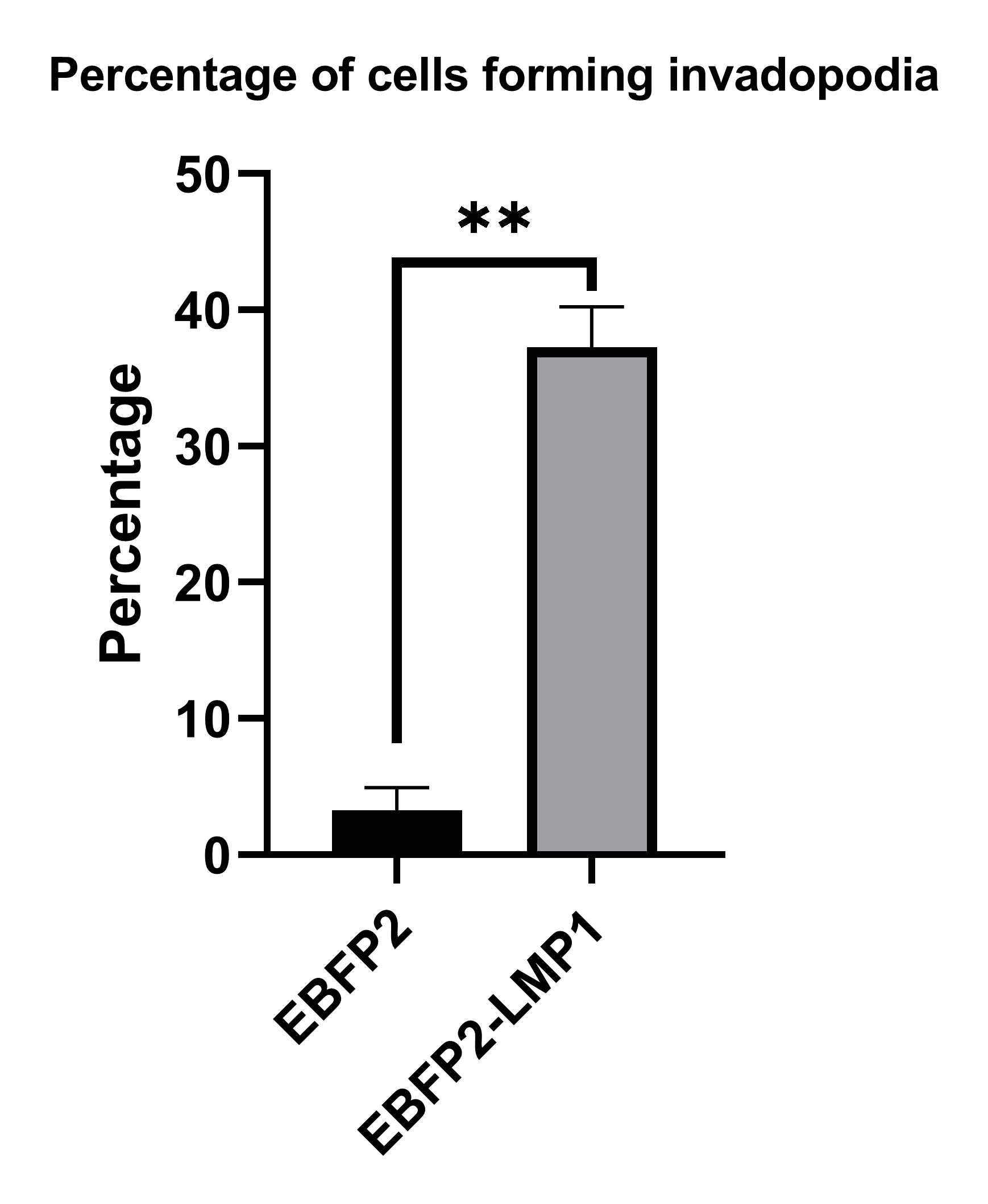
**


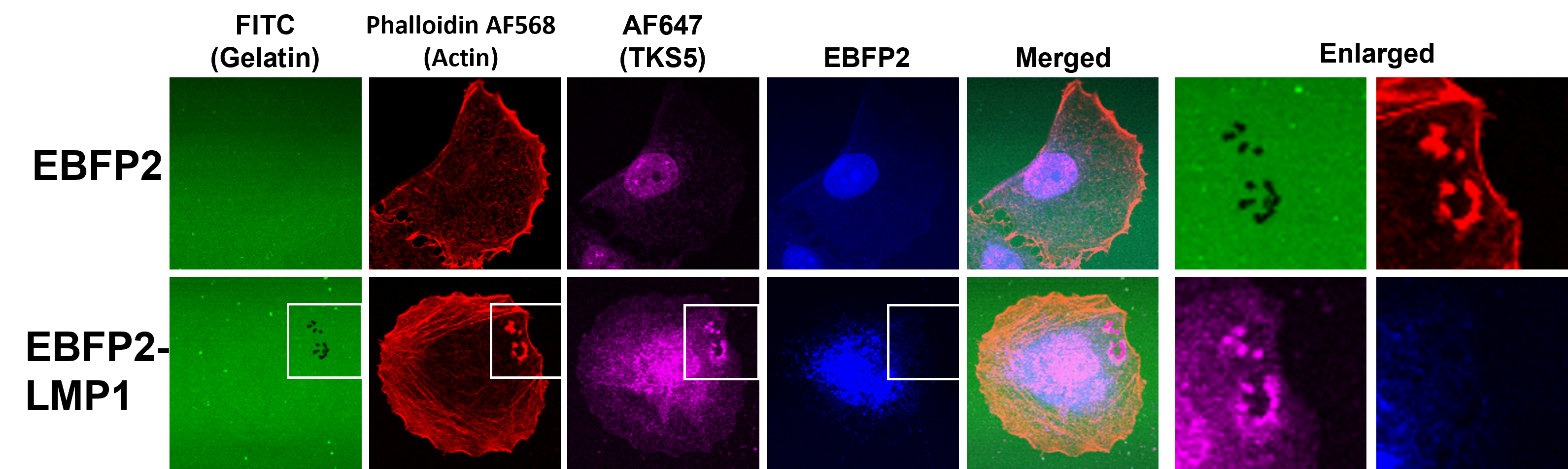


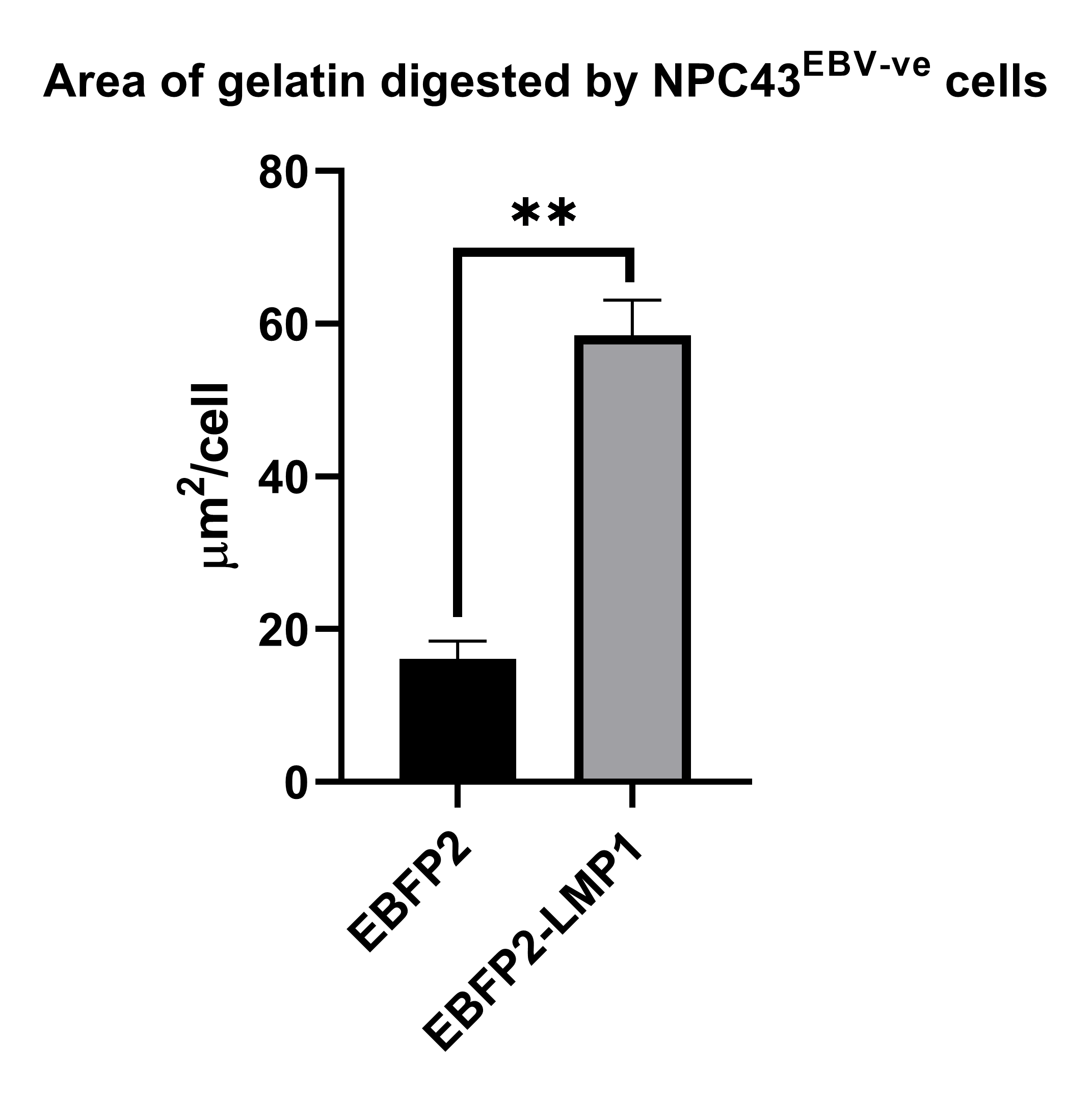


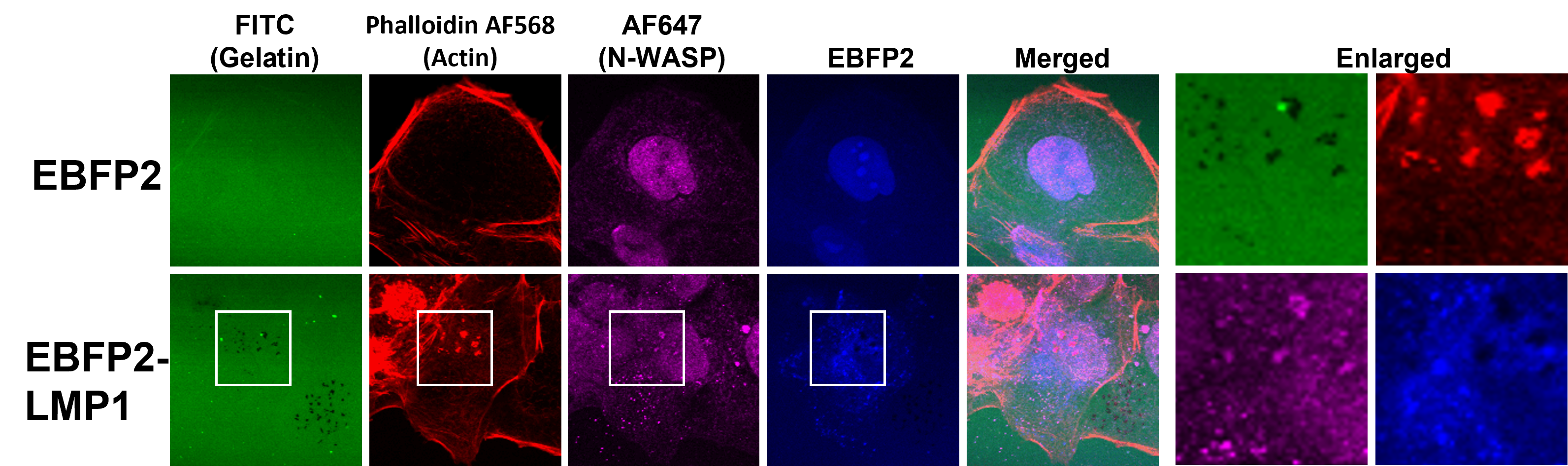


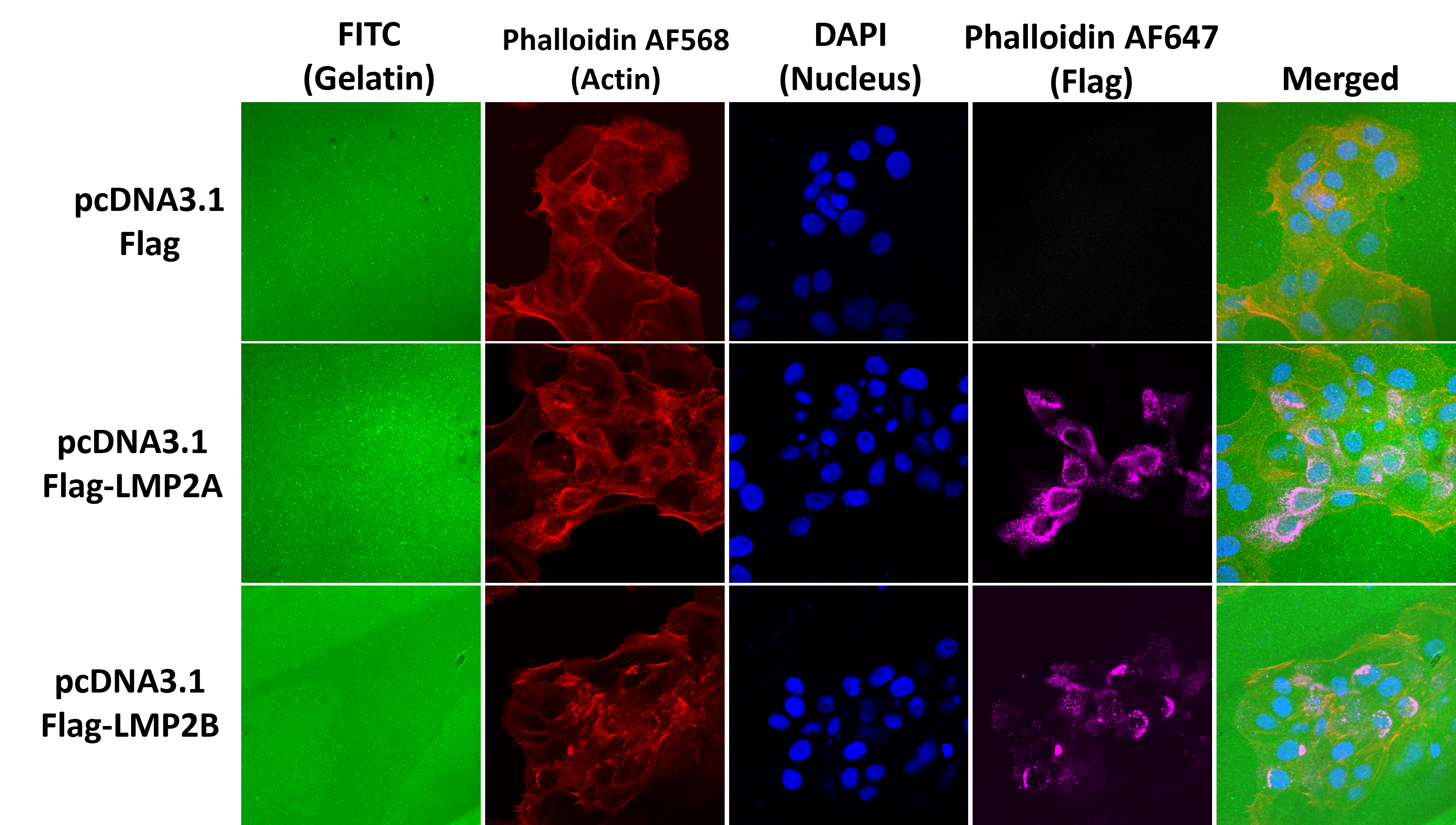

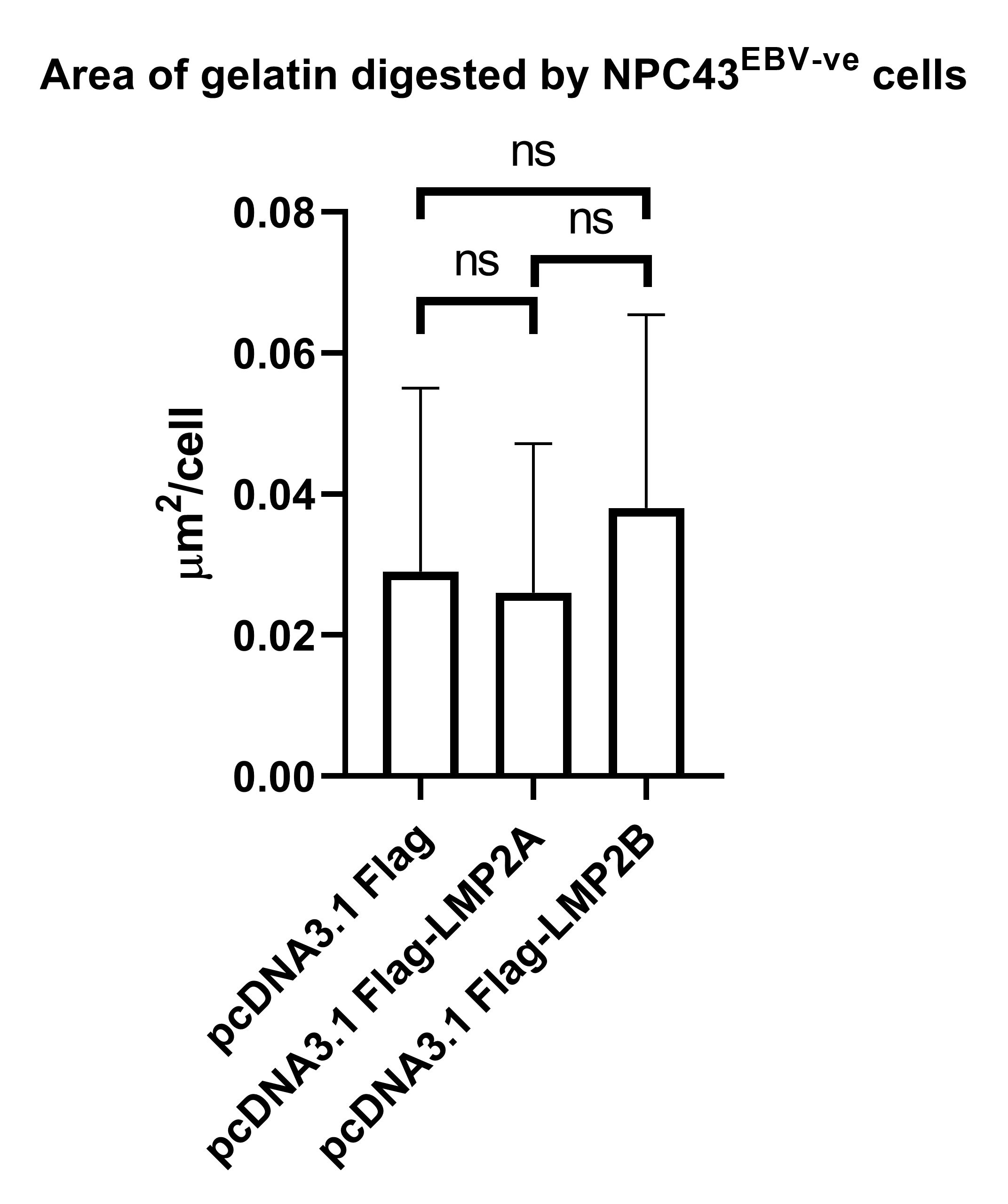
**D**

**E**

**
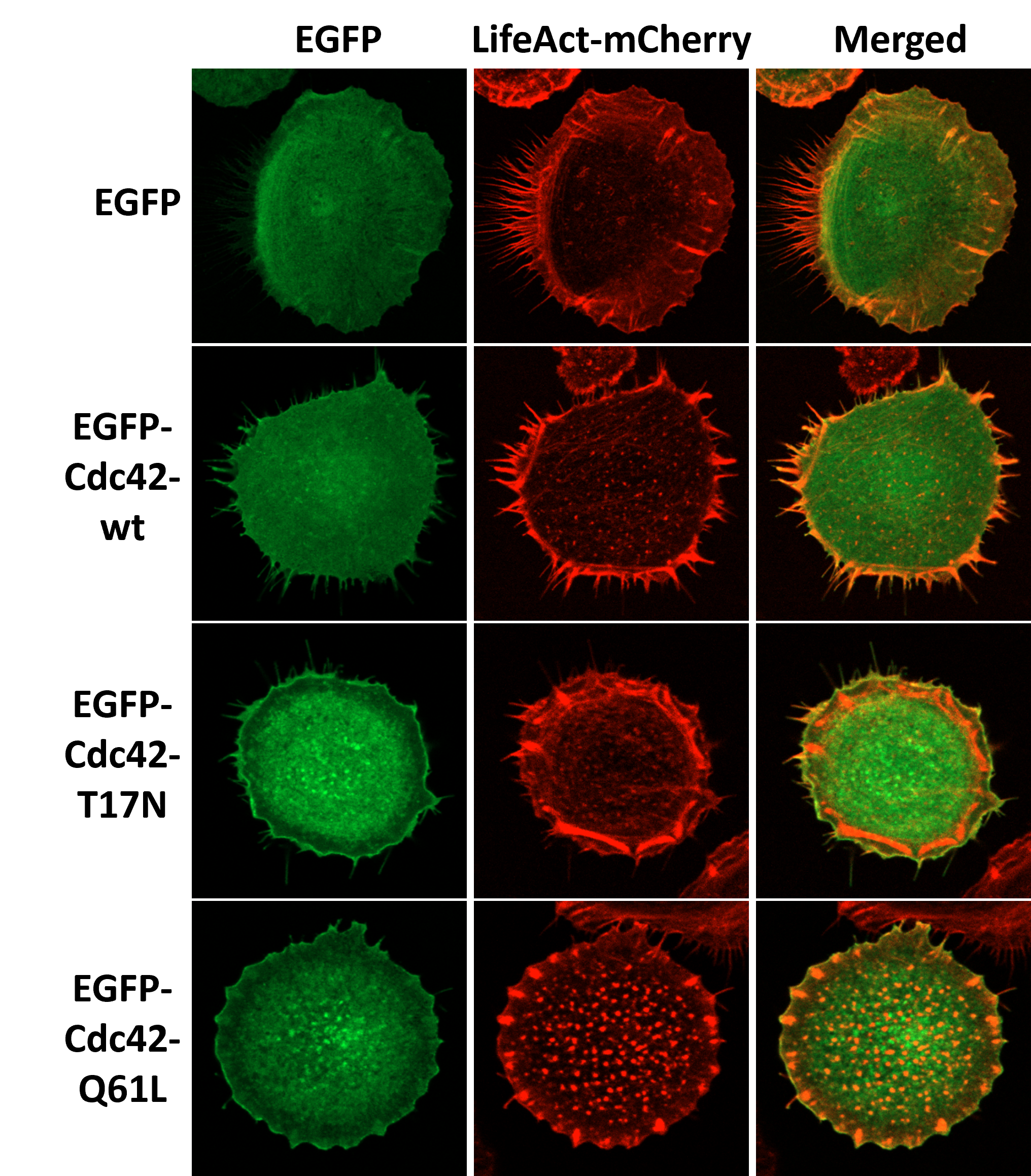
**

**
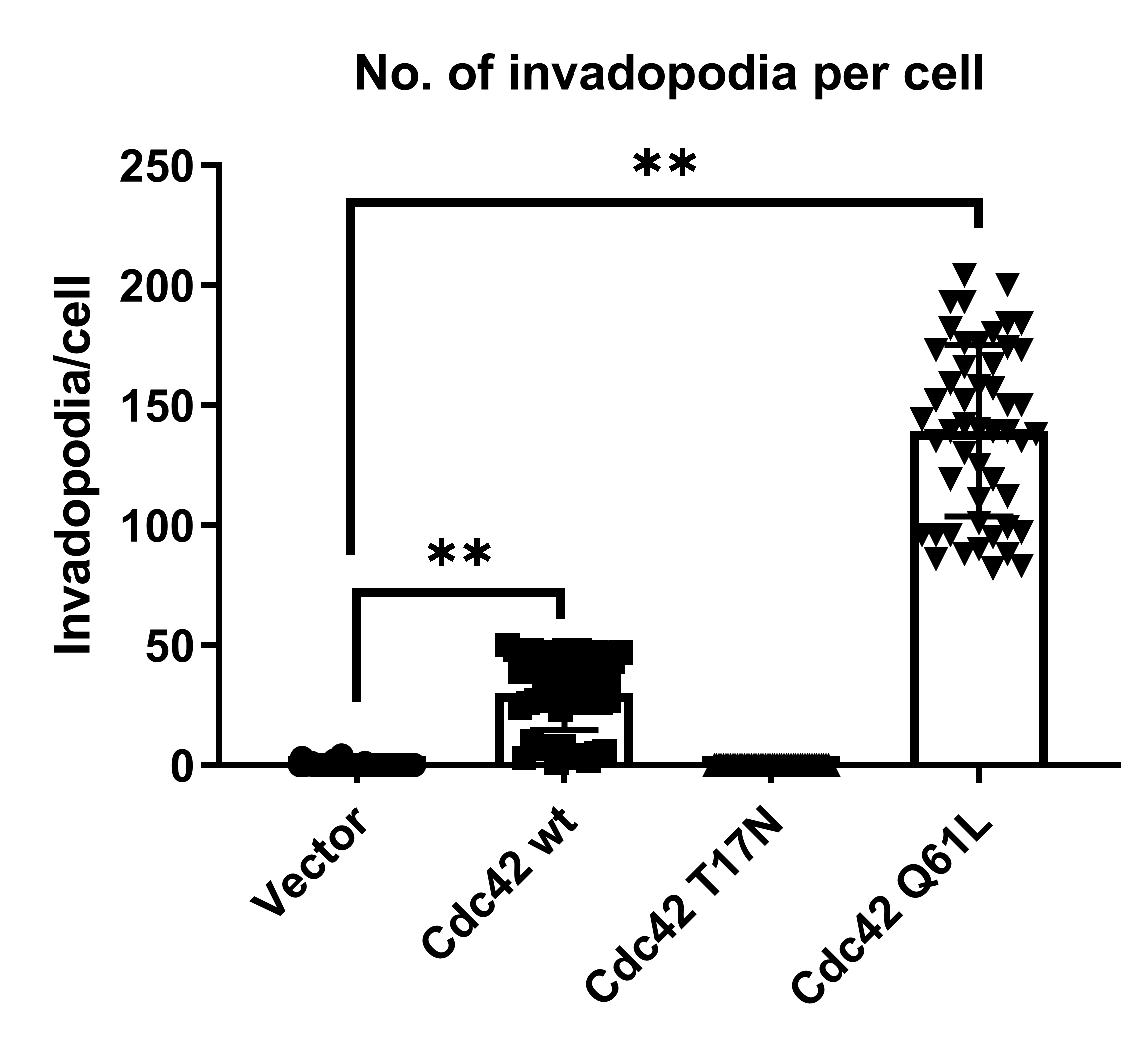
**

**F**


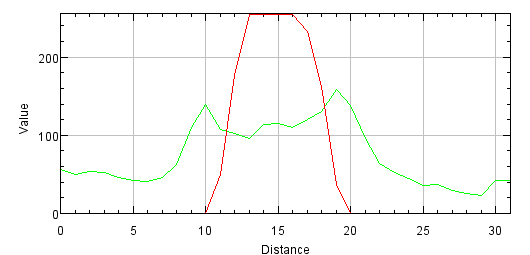

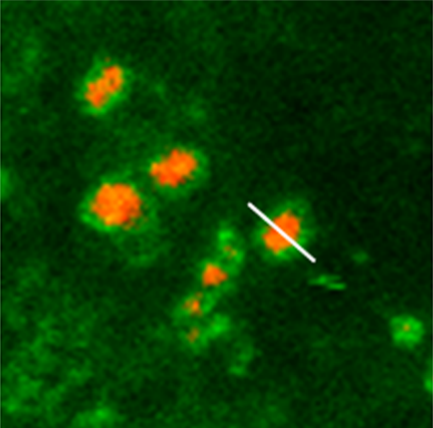

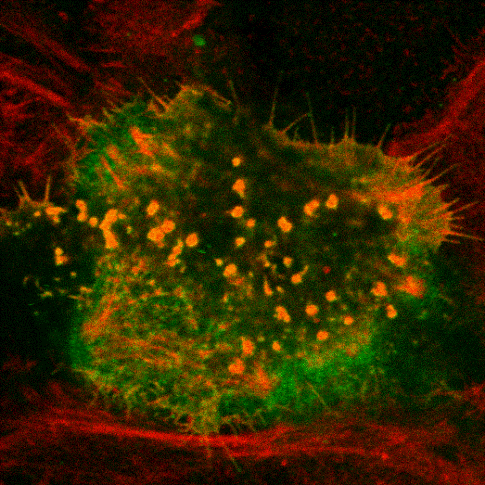


**Red: Phalloidin AF 568**

**Green: EGFP-Cdc42-Q61L**

**Figure S4.** **LMP1 and its downstream Cdc42 could induce invadopodia formation. Related to Figure 4.** (A) Expression profiles of differentially expressed invadopodia-related genes in NPC43^EBV−ve^ and NPC43^EBV+ve^ cells treated with vehicle or TNFα. Means ± SEM. Double asterisks denote values significantly different from control cells (*p* < 0.01). (B) Top: transient transfection of EBFP2-LMP1 induced invadopodia in NP460hTert cells. EBFP2-LMP1 did not co-localize with the invadopodia in the transfected cells. Bottom: statistical analysis of the number of invadopodia induced by EBFP2-LMP1 expression. Means ± SEM. Double asterisks denote values significantly different from control cells (*p* < 0.01). (C) Left: transient transfection of EBFP2 or EBFP2-LMP1 into NPC43^EBV−ve^ cells. The cells were fixed and stained with cortactin, TKS5, and N-WASP, which are the markers of invadopodia. Magnified images of the markers and invadopodia from the white box are presented. Right: the percentage of cells forming invadopodia and the area of gelatin digested were calculated. (D) Top: gelatin degradation assay of NPC43^EBV−ve^ cells transiently transfected with either pcDNA3.1 Flag, pcDNA3.1 Flag-LMP2A, or pcDNA3.1 Flag-LMP2B. There was no significant difference of the gelatin degradative ability between the LMP2A-, LMP2B-, and control-cells. Bottom: statistical analysis of the area of FITC-gelatin digested per cell. Means ± SEM. (E) Left: transient transfection of EGFP, EGFP-Cdc42, or EGFP-Cdc42 mutants in NP460hTert cells. Right: statistical analysis of the number of invadopodia induced by EGFP, EGFP-Cdc42, or EGFP-Cdc42 mutants. The activated form of Cdc42 (Q61L) potently induced invadopodia formation in NP460hTert cells. All values are means ± SEM. Double asterisks denote values significantly different from control cells (*p* < 0.01). (F) EGFP-Cdc42-Q61L formed a ring structure surrounding the invadopodium. Top: confocal image showing a Cdc42-Q61L-transfected cell and a magnified region-of-interest for fluorescent signal analysis. Bottom: an RGB profile showing the cross-section (white line) of an invadopodium. Scale bar: 0.1 µm. Means ± SEM. Student’s *t*-test *P* value indicated the significant difference among the compared groups (***p* < 0.01). Each of the above experiments was repeated three times (*N* = 3).

**A**

**
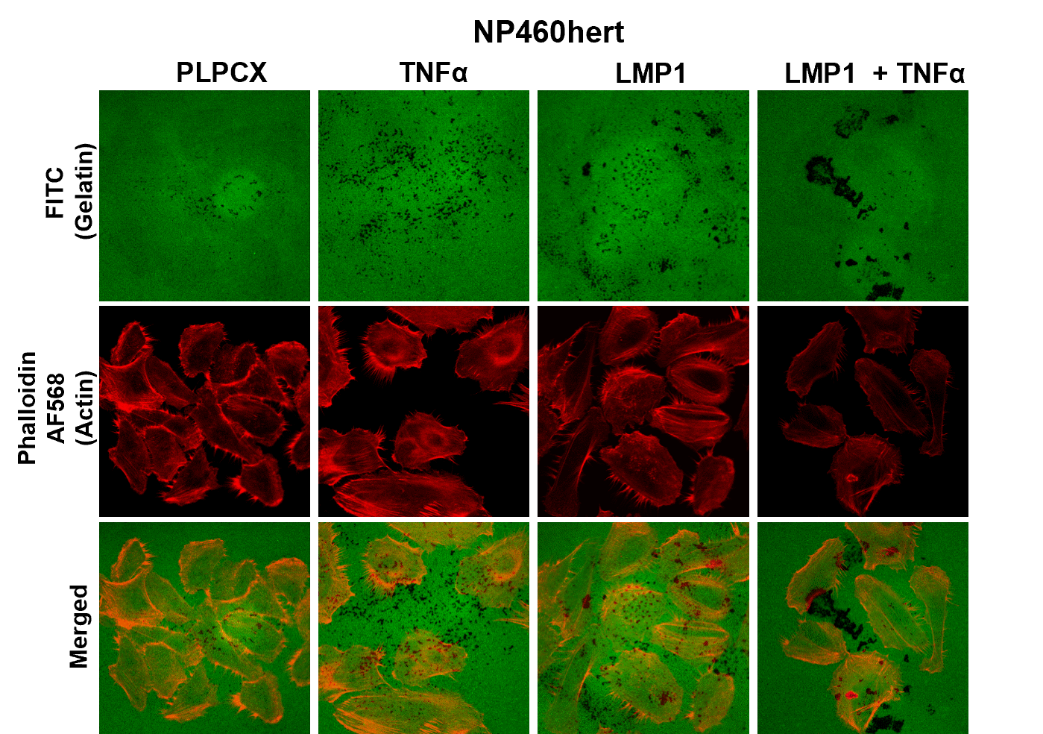
**

**
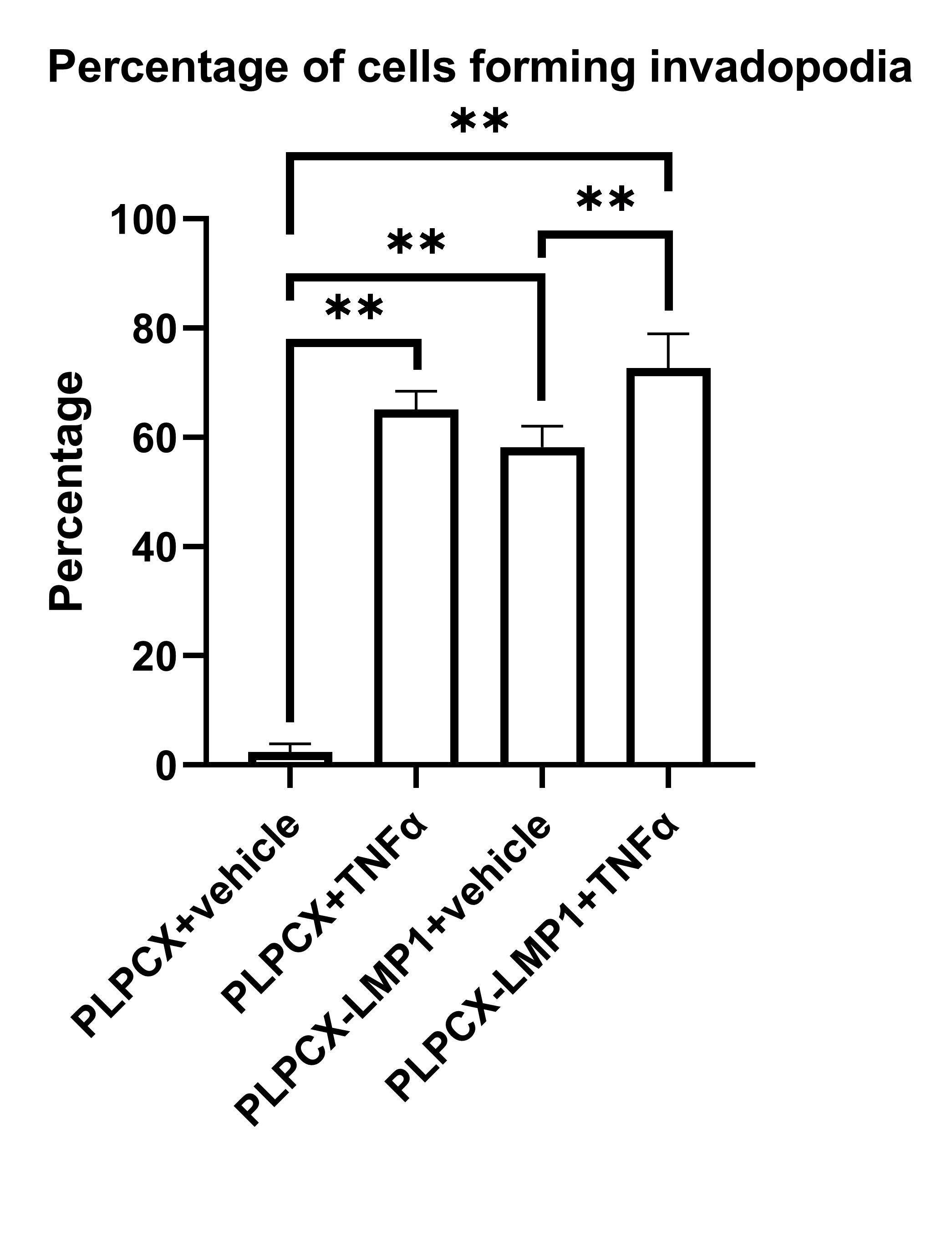

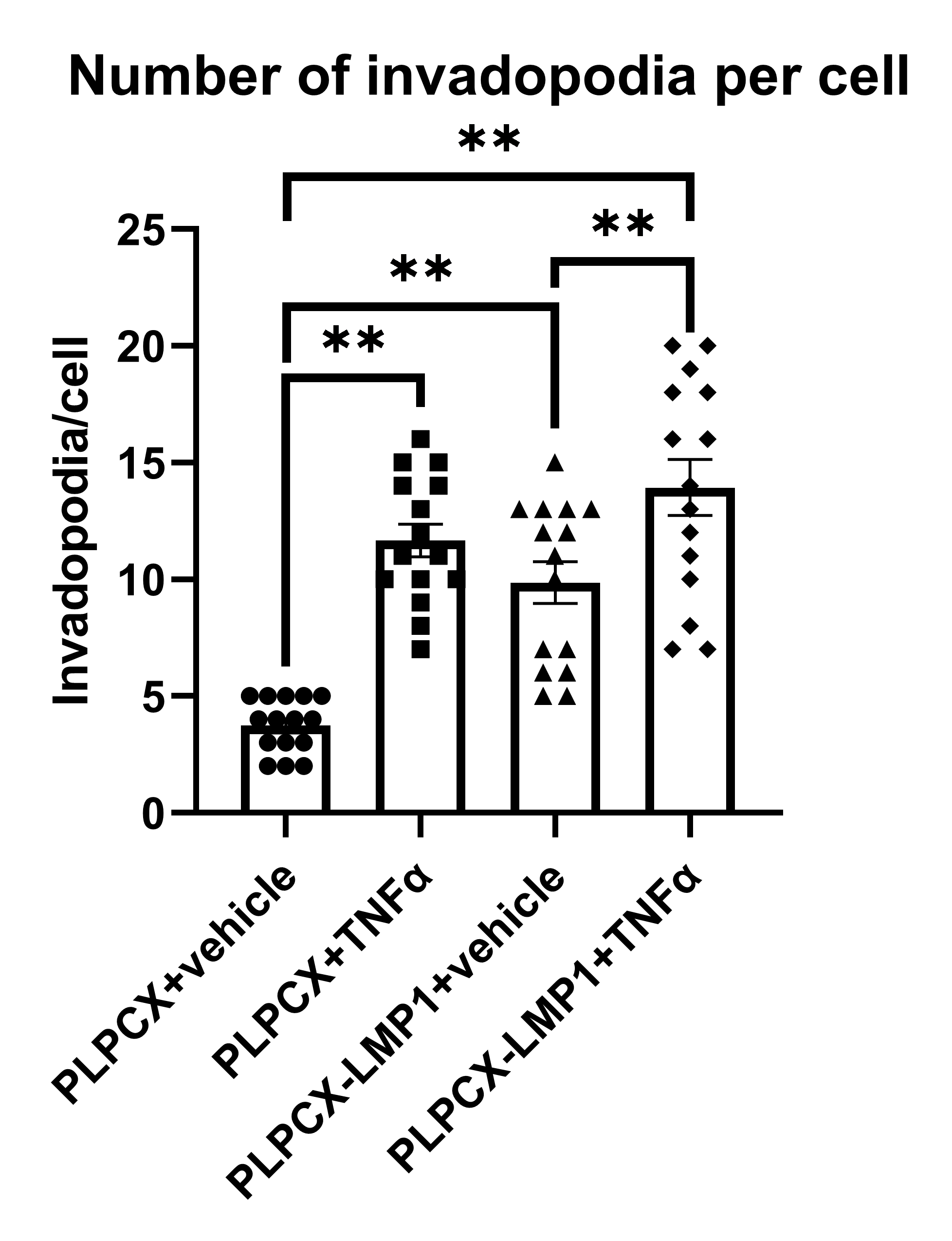
**

**
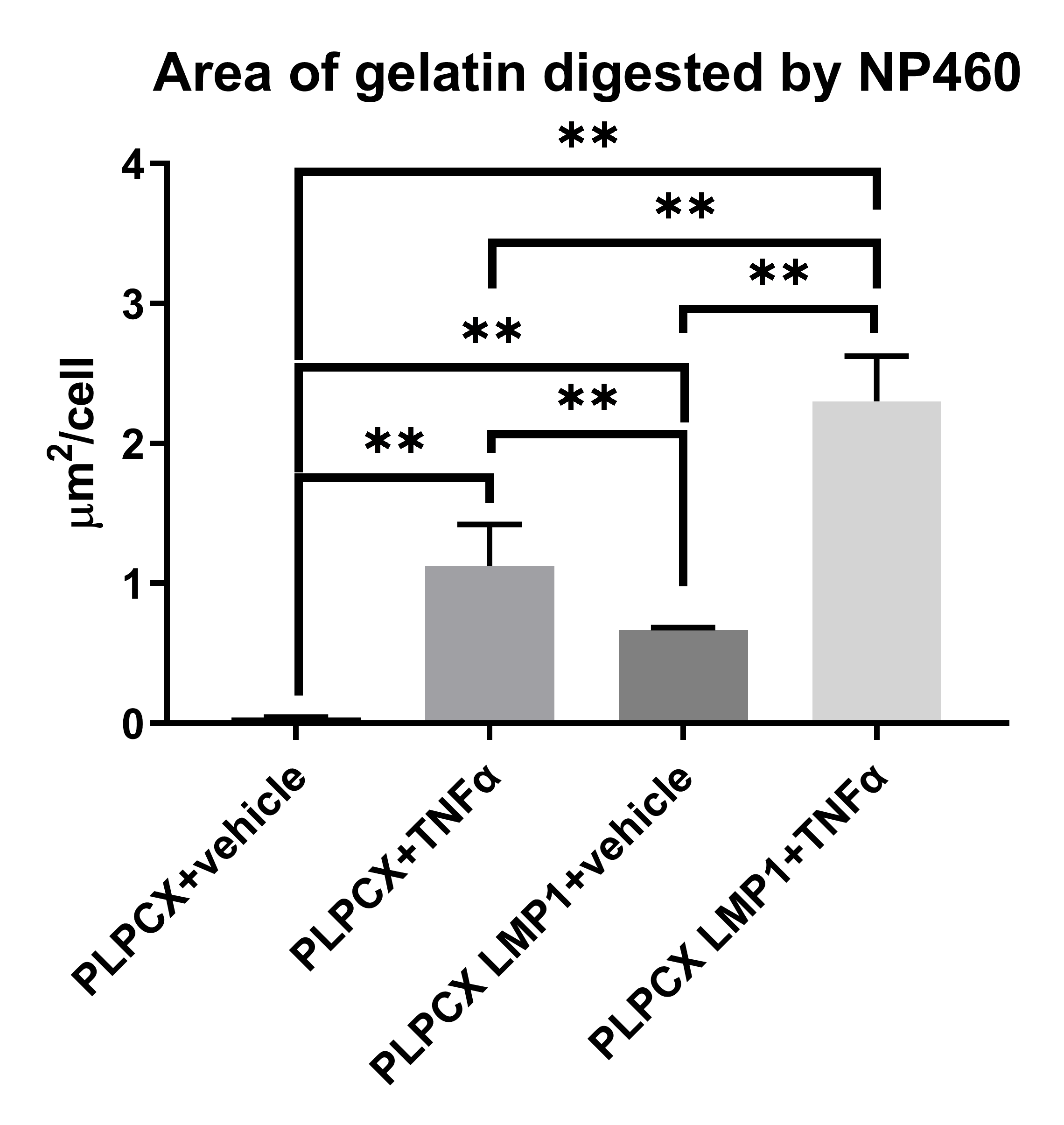
**

**B C**

**
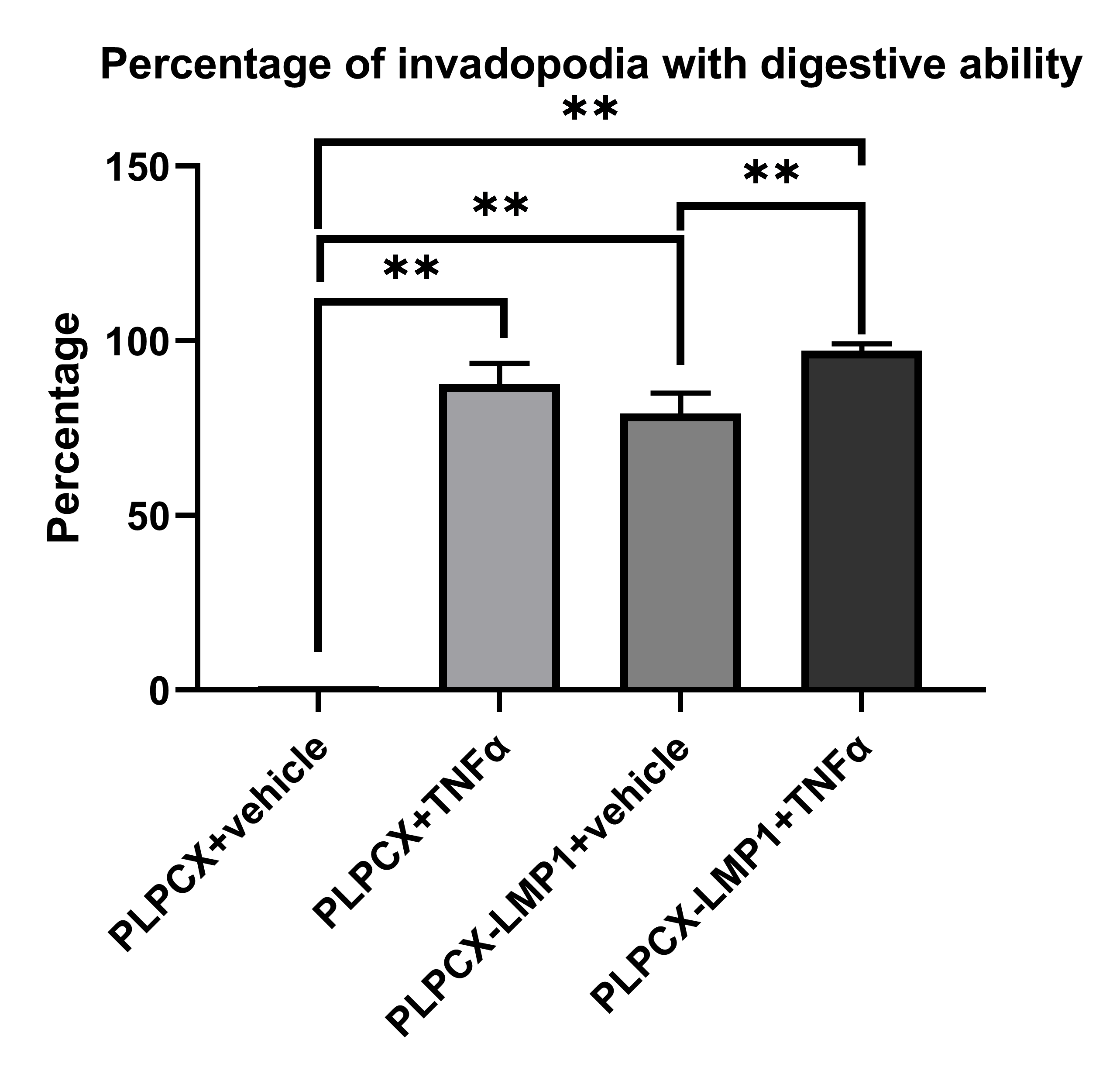

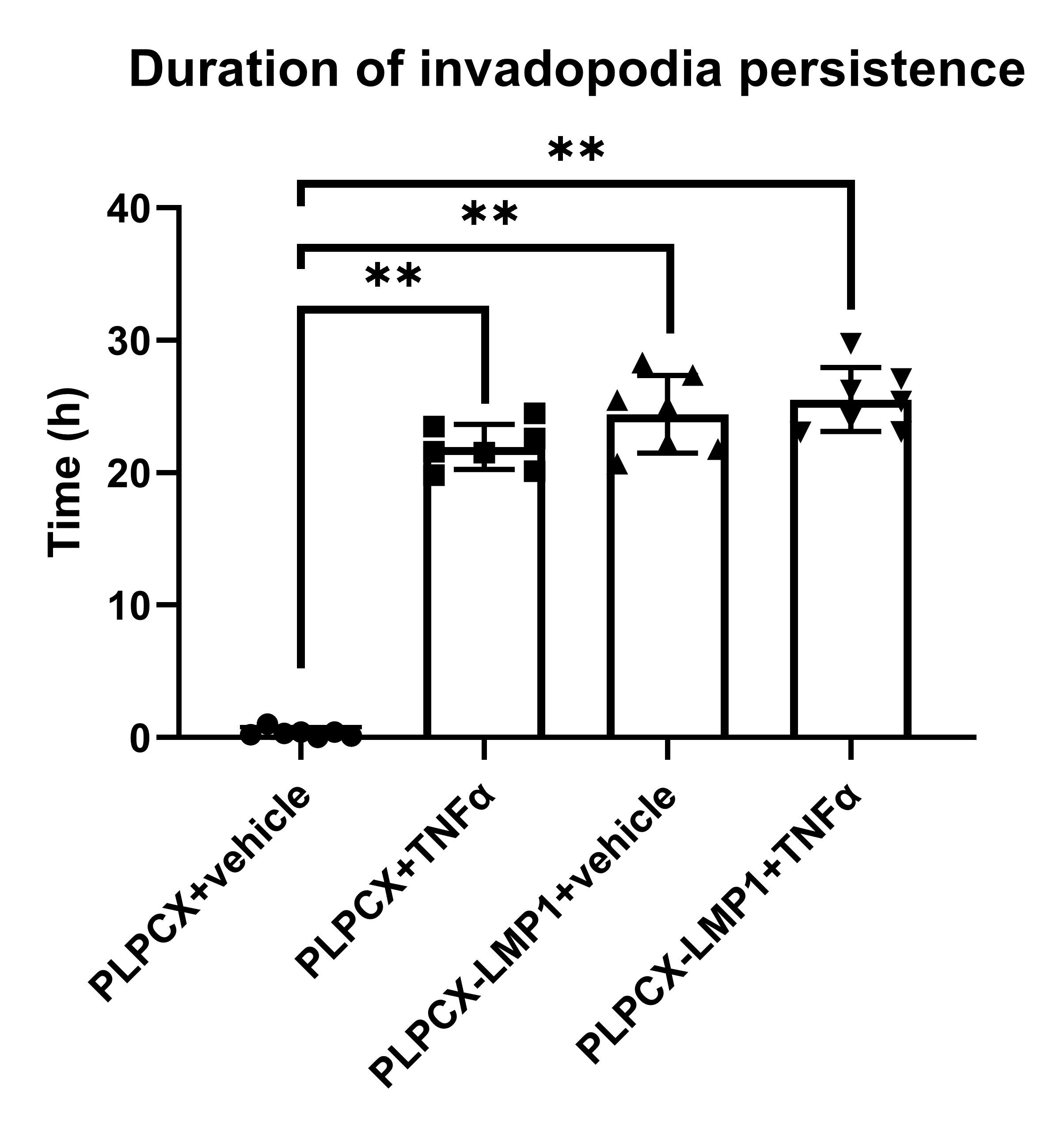
**


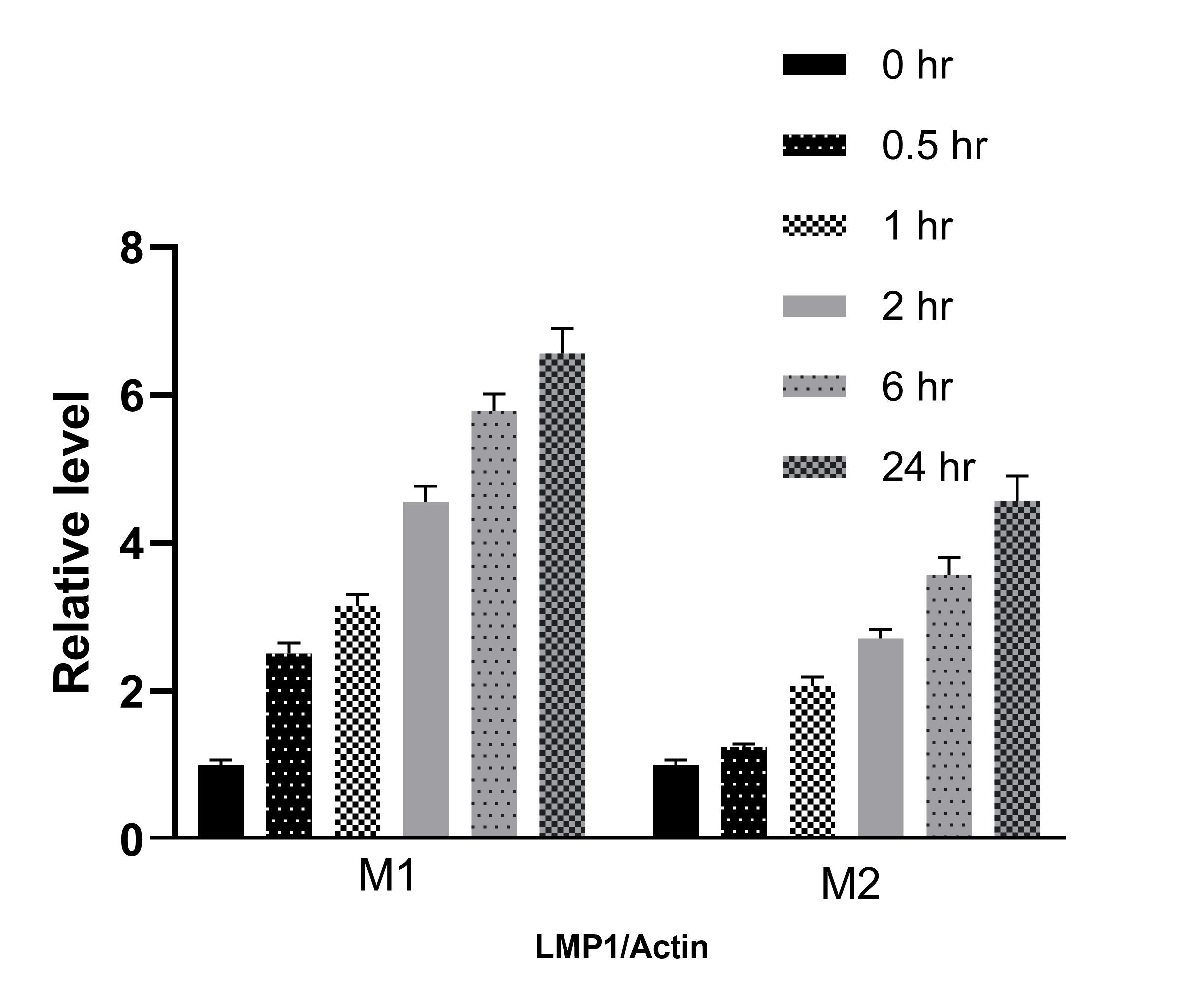
**D**

**E**

NP460hTert e


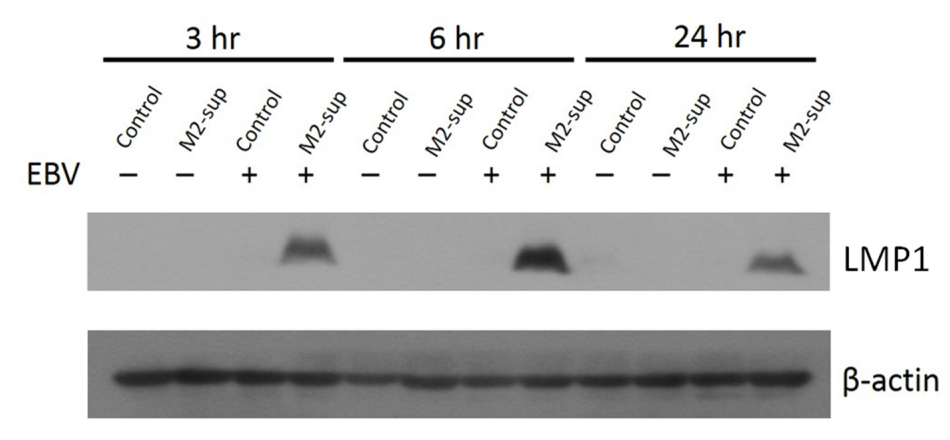


**
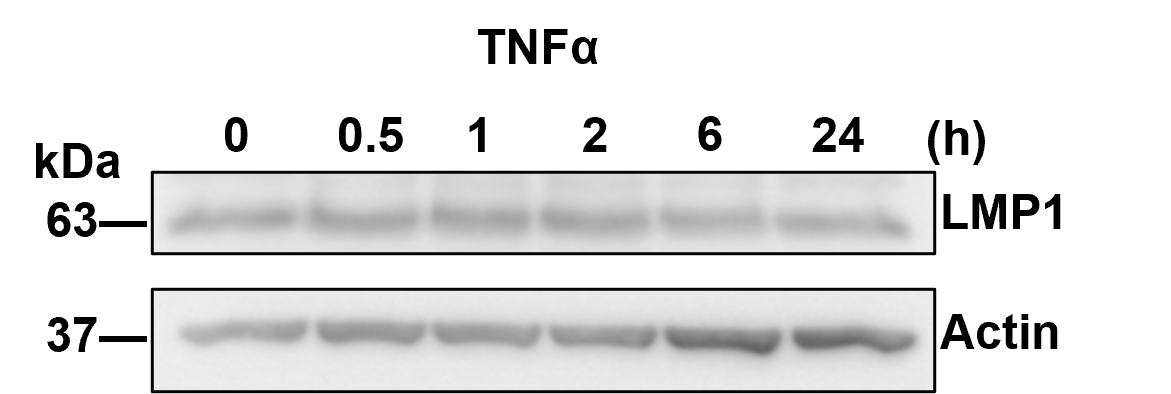
**

**F**


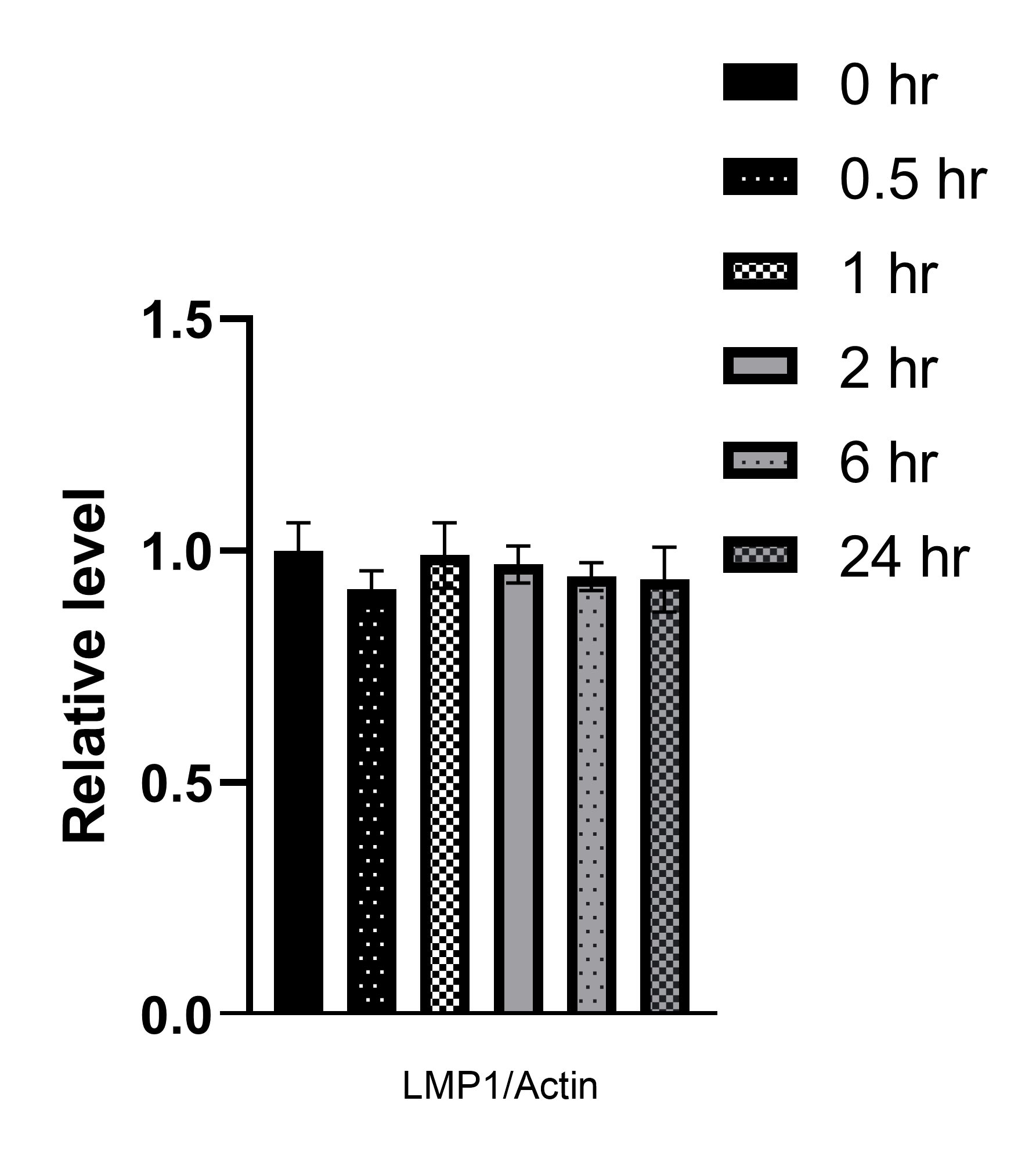


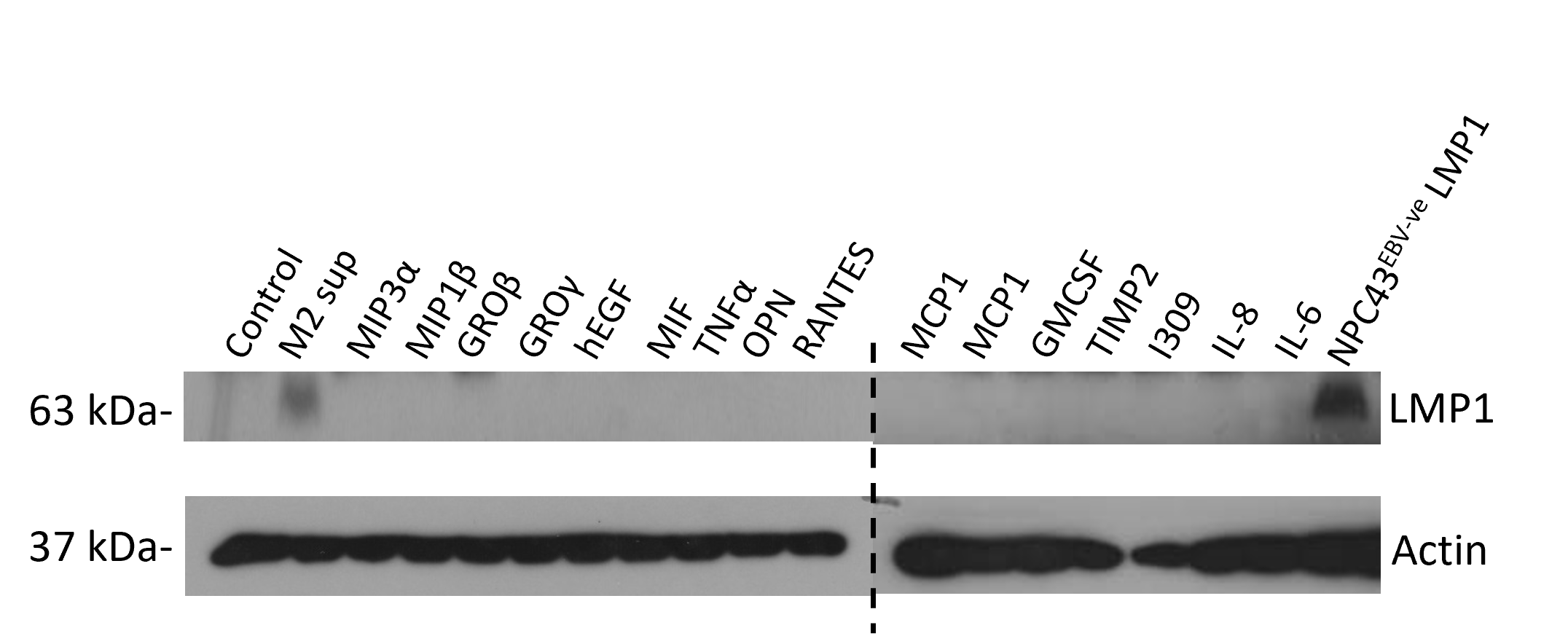


**G**

**H**


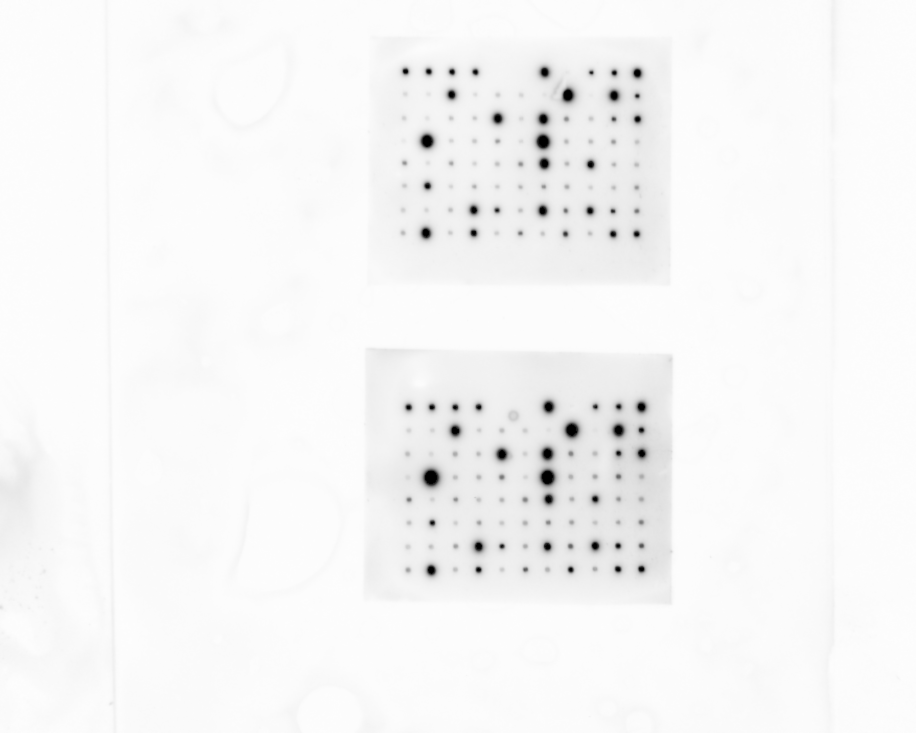

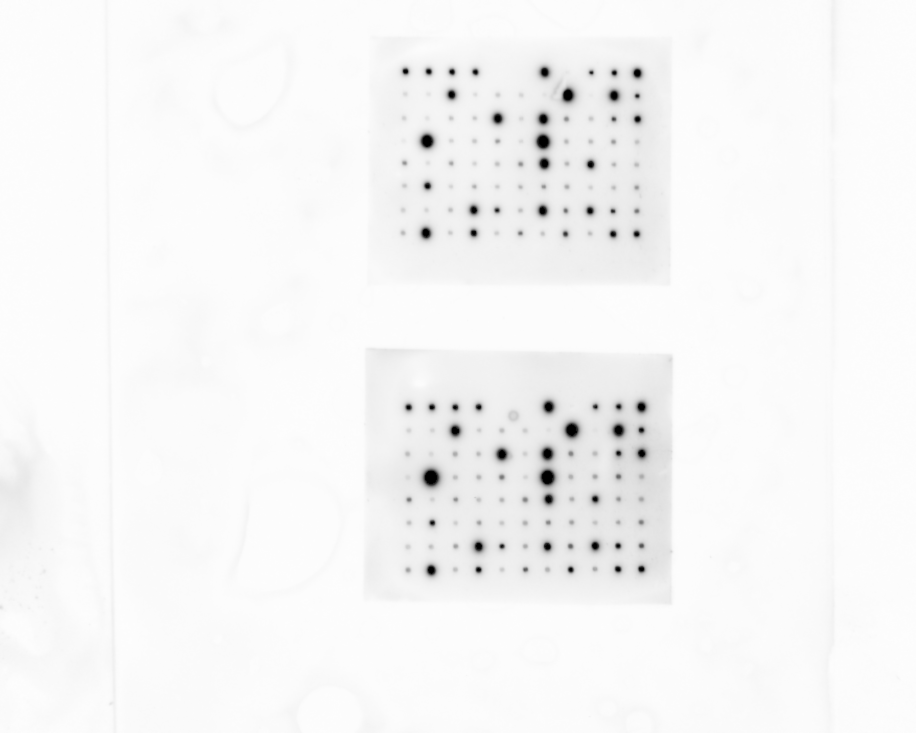

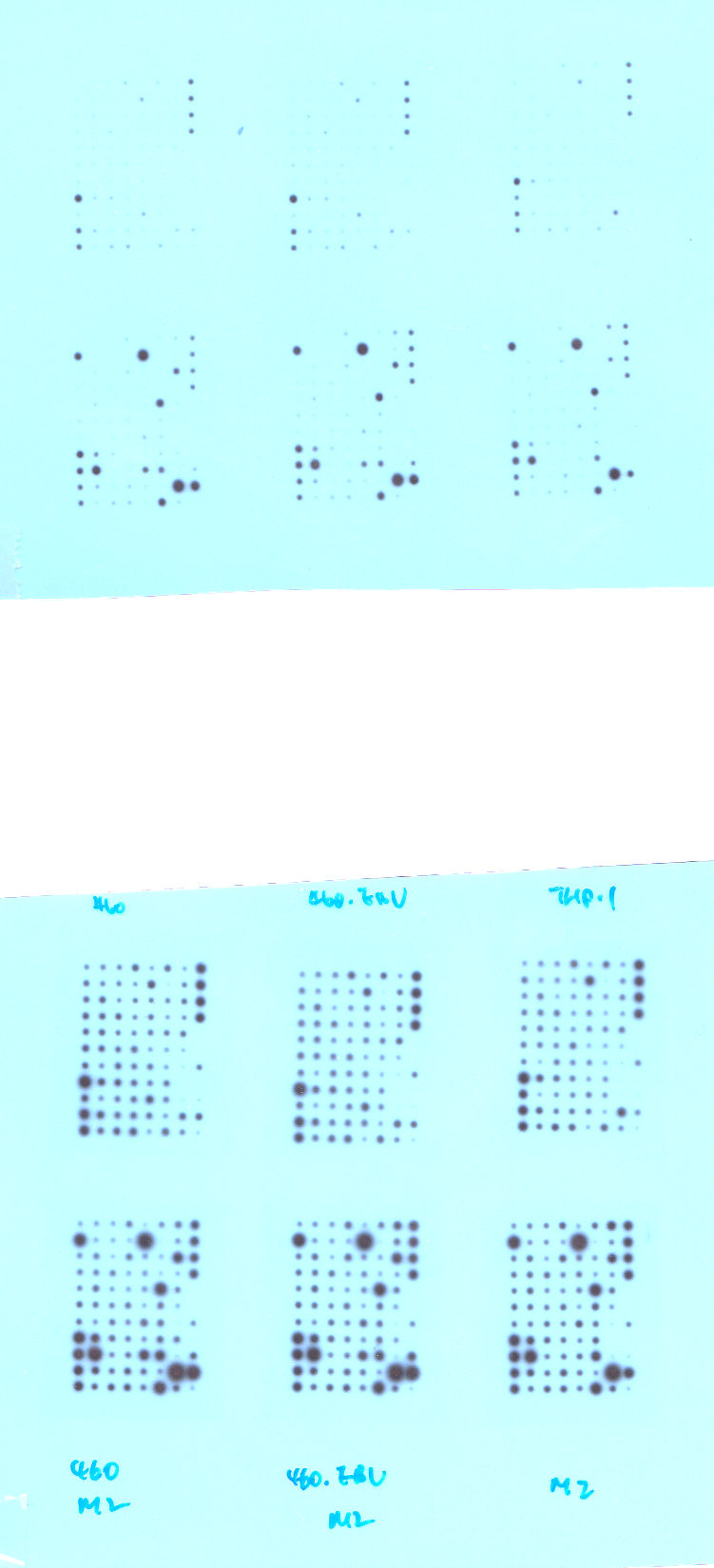

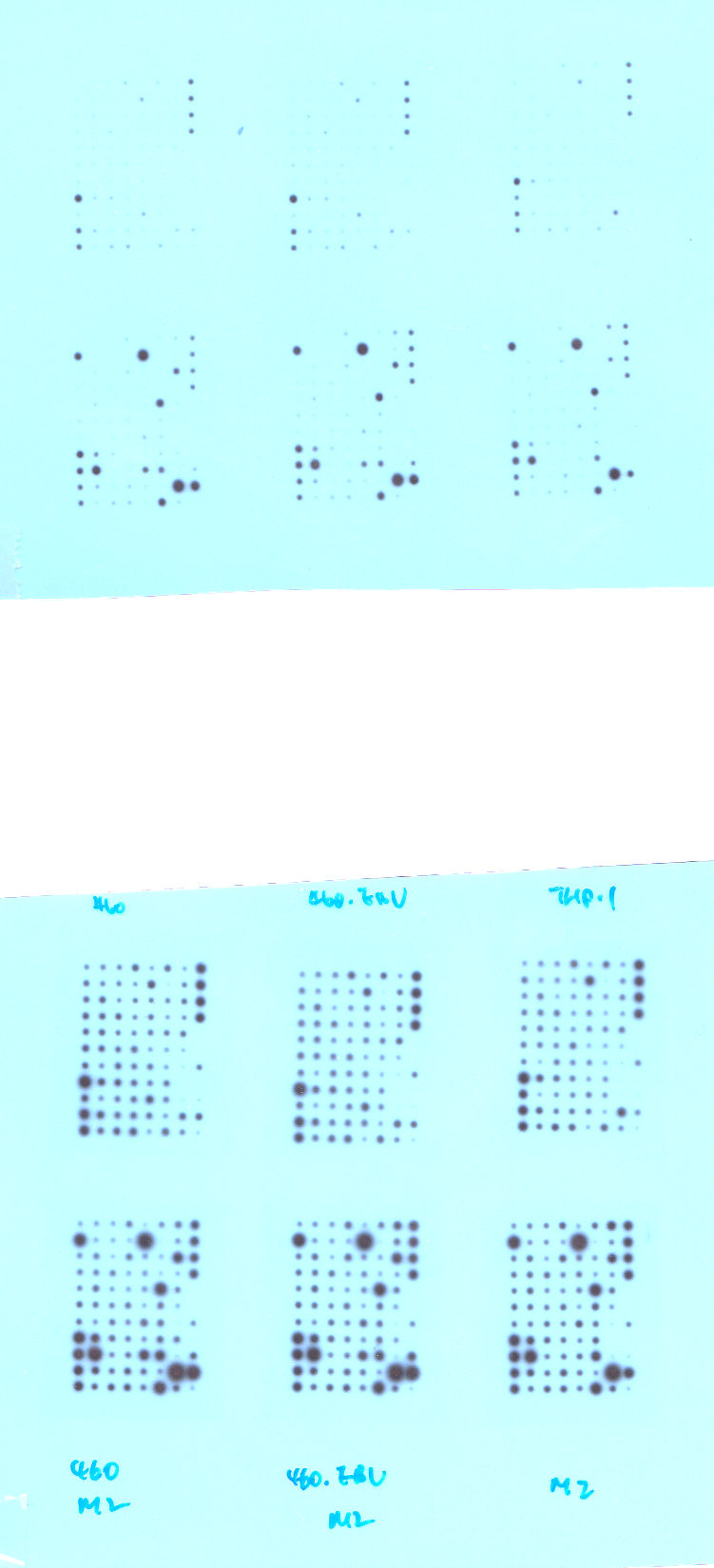


Supernatant from M1-THP1 co-culturing with NPC cells in a transwell

Supernatant from M1-THP1

Supernatant from unactivated THP-1

Supernatant from NP cells


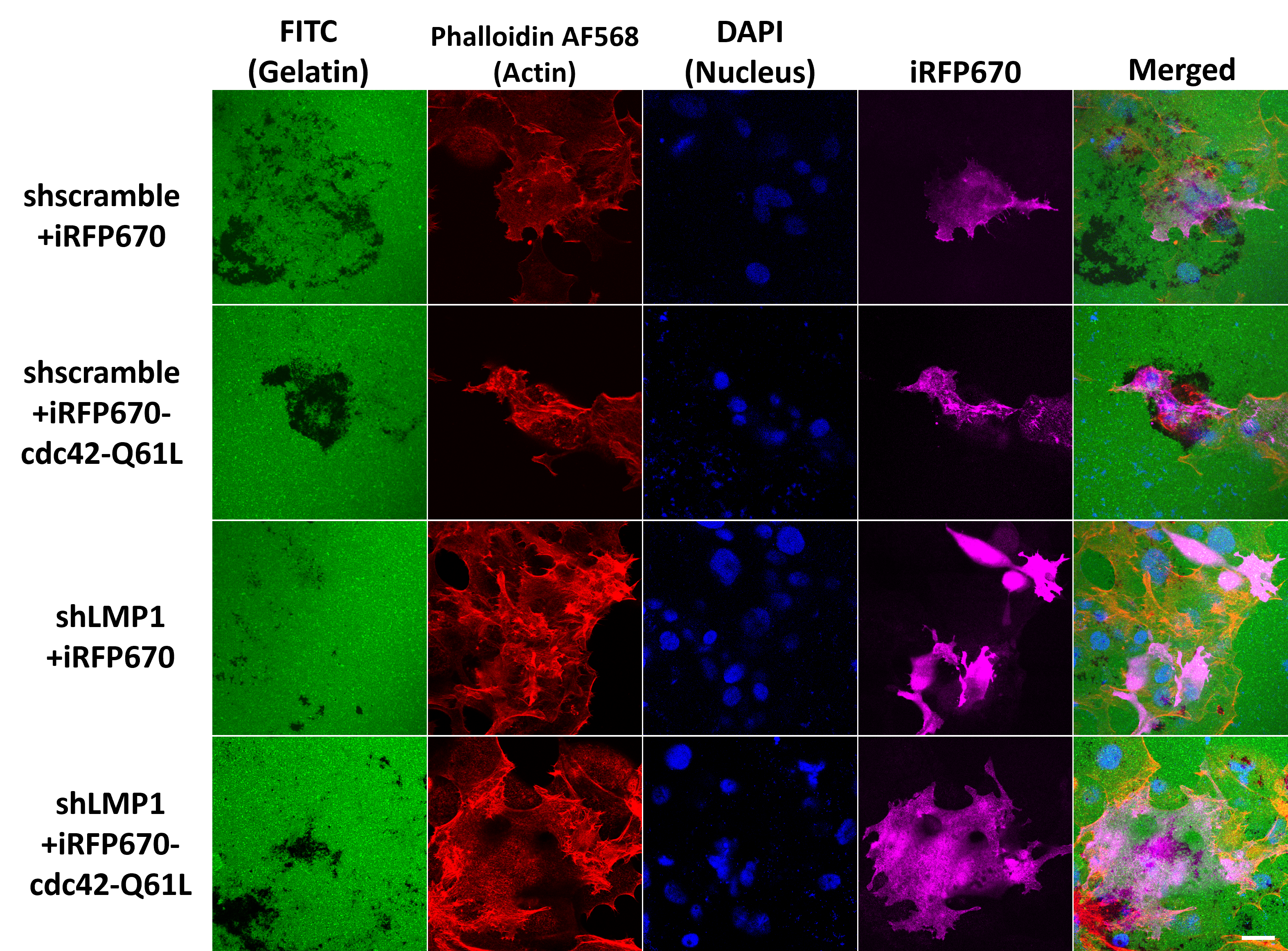
**I J**

**
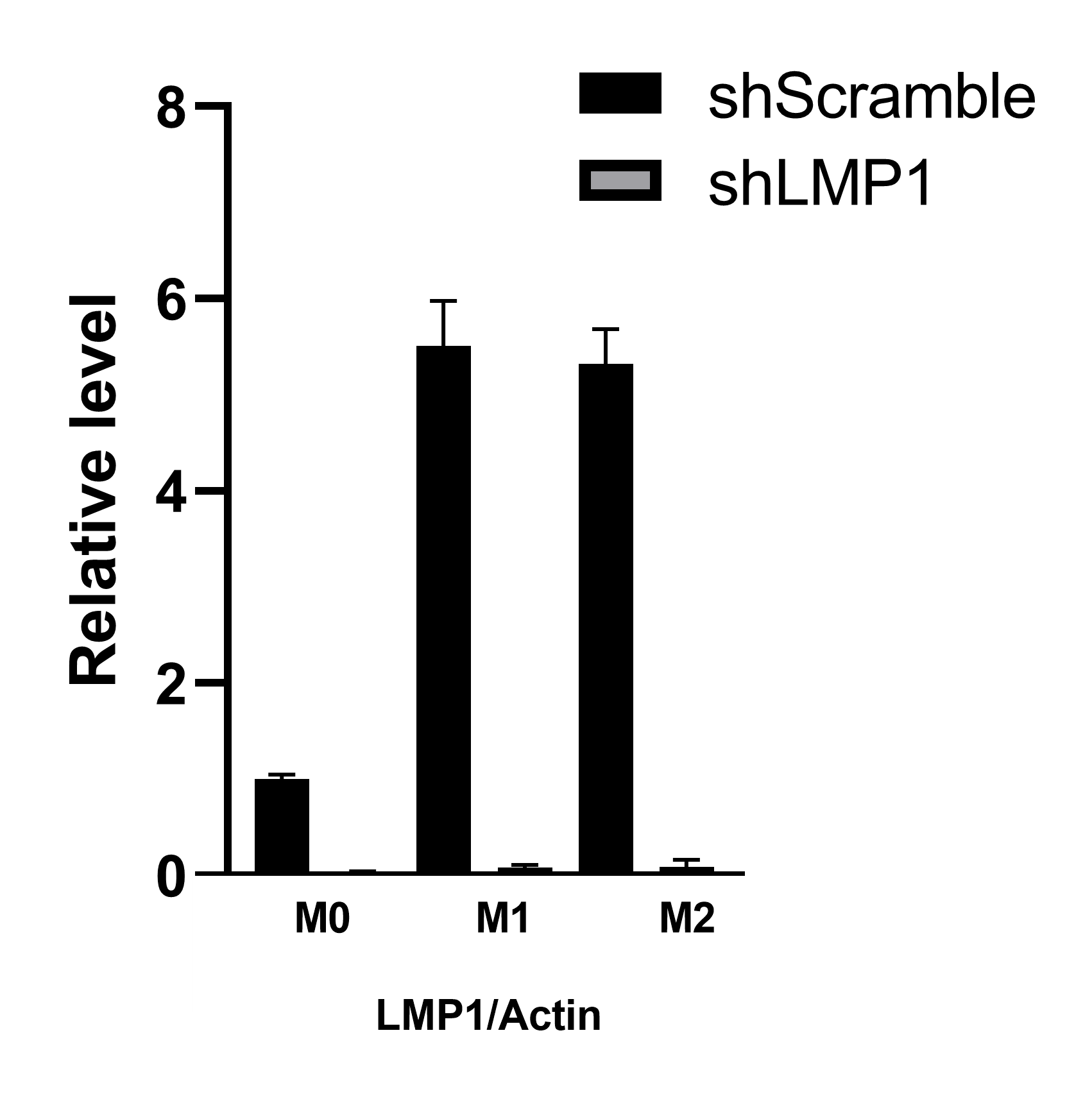
**

**
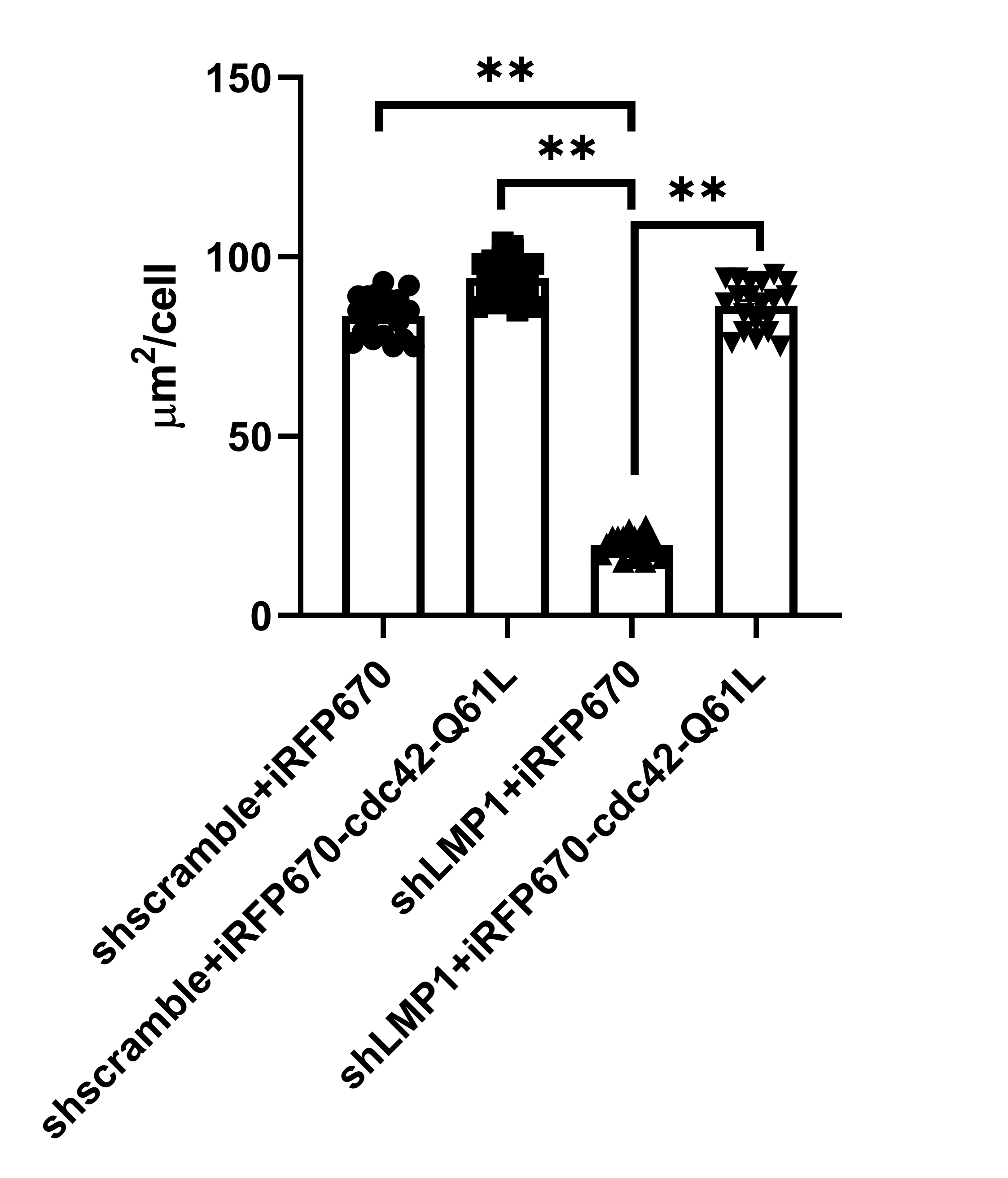
**

**Figure S5.** **LMP1 and TNFα synergistically induce invadopodia in NP460 cells. Related to Figure 5.** (A) Top: NP460hTert cells stably expressing the control vector or LMP1 were treated with vehicle or TNFα for 24 h. Bottom: statistical analysis of the area of FITC-gelatin digested per cell, the percentage of cells forming invadopodia, and the number of invadopodia per cell. Means ± SEM. Double asterisks denote values significantly different from control cells (*p* < 0.01). (B) Percentage of invadopodia with digestive ability (related to Figure 5B–E). (C) The duration of invadopodia persistence in cells from different treatment groups (related to Figure 5B–E). Means ± SEM. Double asterisks denote values significantly different from control cells (*p* < 0.01). (D) Quantification of western blot signals in Figure 5G. The values reported under each blot are the mean fold of protein expression relative to vehicle control taken as 1 after normalization by actin (ImageJ quantification). (E) M2-like macrophage supernatant induced the expression of LMP1 in EBV-infected NP460hTert cells. Western blot analysis showing that LMP1 expression was upregulated in EBV-positive NP460hTert cells following addition of macrophage-conditioned medium. (F) Top: western blot analysis showing that TNFα (10 ng/ml) did not modulate the expression of LMP1 in NPC43 cells. Bottom: quantification of the signals in the western blots. (G) Western blot analysis of NPC43 cells treated with M2 supernatant or cytokines. Individual addition of these cytokines could not upregulate the LMP1 expression in the NPC43 cells. (H) Cytokine array analysis showed that the co-culturing of NPC43 cells (in the Transwell) did not increase the secretion of TNFα from the M1-THP1 cells. The red rectangles enclose the blots of TNFα. (I) Quantification of western blot signals in Figure 5H. The values reported under each blot are the mean fold of LMP1 expression relative to that of the M0-treated cells transfected with shScramble after normalization by actin (ImageJ quantification). (J) Gelatin degradation assay of NPC43 shscramble/shLMP1 transiently transfected with iRFP670/iRFP670-cdc42-Q61L under the stimulation of M1 supernatant. Gelatin degradation assay showed that NPC43 cells with overexpression of iRFP670-cdc42-Q61L (activated form) could rescue the loss of degradative power in the LMP1-knockdown cells. This indicates that LMP1 exerts its effect through Cdc42 activation. Statistical analysis of the area of FITC-gelatin digested per cell is shown in the bar chart. Means ± SEM. Student’s *t*-test *P* value indicated the significant difference among the compared groups (***p* < 0.01). Each of the above experiments was repeated three times (*N* = 3).


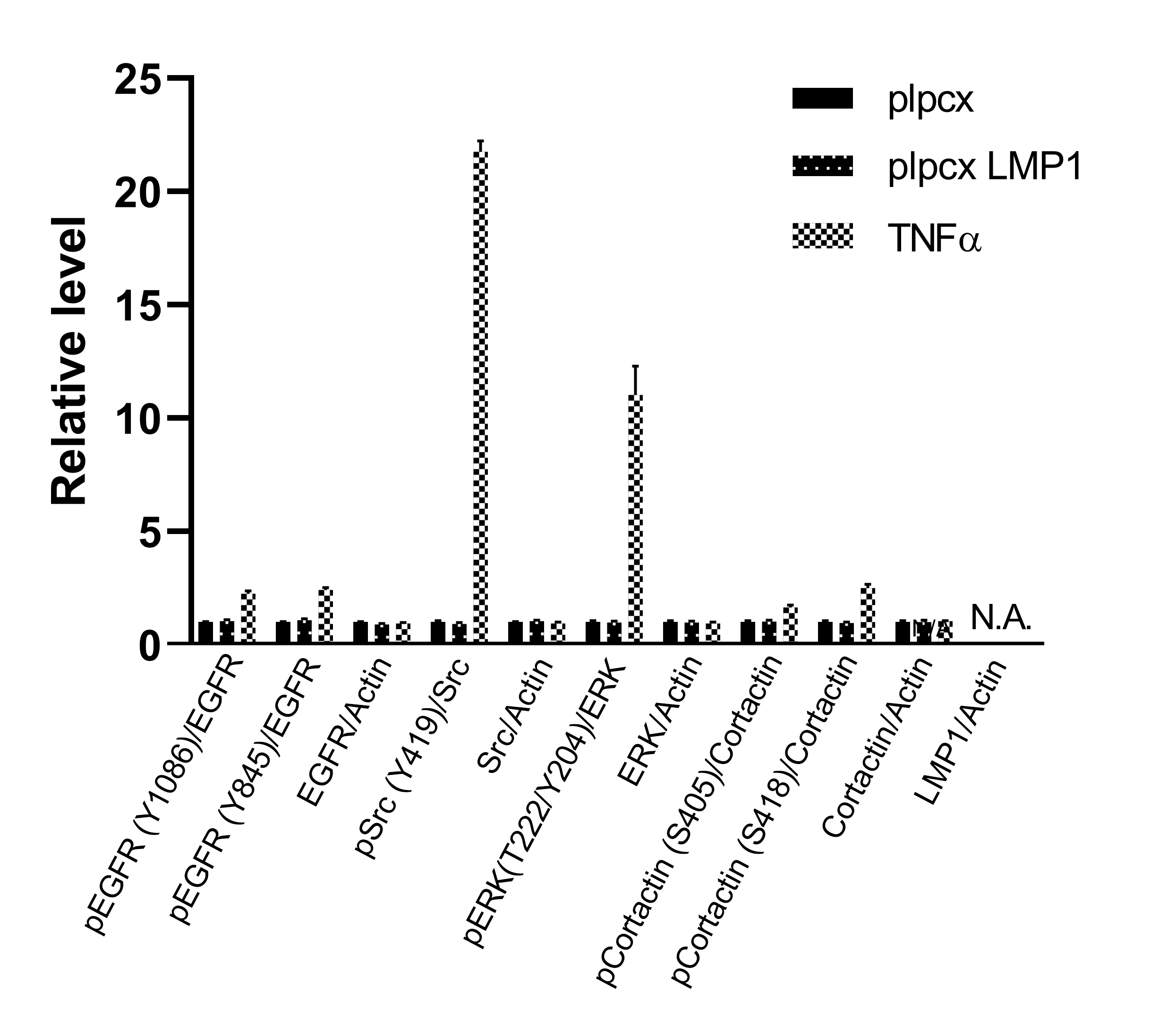
**A**


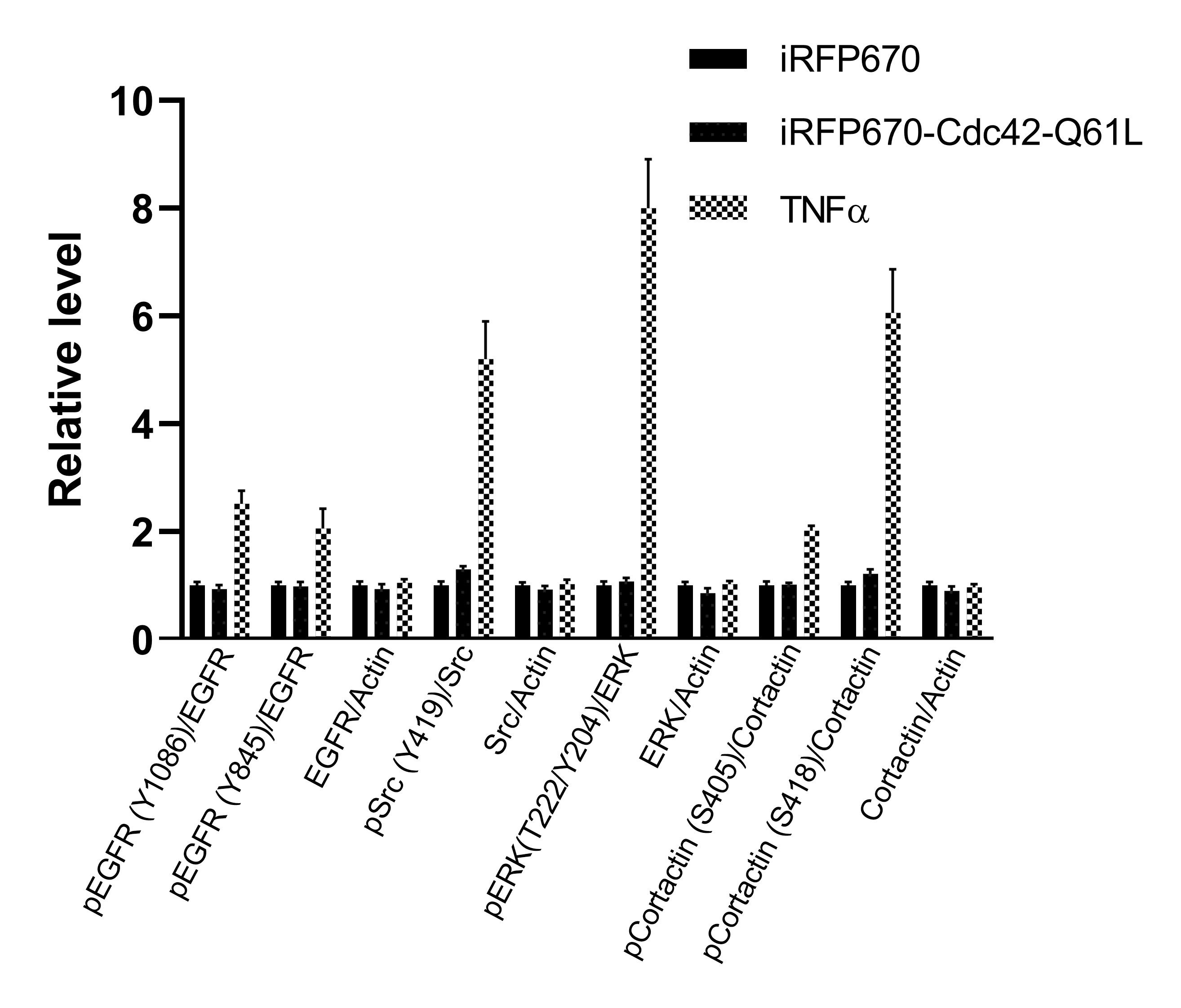
**B**

**Figure S6. Quantification of the western blots of Figure 6.** (A, B) Quantification of the signals in the western blots of Figure 6C and 6D. The values reported under each blot are the mean fold of protein expression relative to vector controls taken as 1 after normalization by the non-phosphorylated form of the corresponding protein or actin (ImageJ quantification). Means ± SEM. Student’s *t*-test *P* value indicated the significant difference among the compared groups (***p* < 0.01). Each of the above experiments was repeated three times (*N* = 3).

**Table S1. Key resources.**

| **Antibodies** | | **Source** | | | **Identifier** | | | | | **Dilution** | |
| --- | --- | --- | --- | --- | --- | --- | --- | --- | --- | --- | --- |
| Actin (I-19) | | Santa Cruz Biotechnology, Dallas, TX, USA | | | SC-1616  RRID:AB_630836 | | | | | 1:1,000 | |
| Phospho-EGF receptor (Tyr845) | | Cell Signaling Technology, Danvers, MA, USA | | | 2231S; RRID:AB_1264155 | | | | | 1:1,000 | |
| Rabbit anti-phospho-EGFR (Tyr1086) | | Thermo Fisher Scientific, Inc., Waltham, MA, USA | | | 36-9700; RRID:AB_2533287 | | | | | 1:1,000 | |
| EGFR (1005) | | Santa Cruz Biotechnology | | | SC-03; RRID:AB_631420 | | | | | 1:1,000 | |
| Phospho-Src family (Tyr416) (D49G4) rabbit mAb | | Cell Signaling Technology | | | 6943S; RRID:AB_10013641 | | | | | 1:1,000 | |
| Src (32G6) rabbit mAb | | Cell Signaling Technology | | | 2123S; RRID:AB_2106047 | | | | | 1:1,000 | |
| p44/42 MAPK (ERK1/2) | | Cell Signaling Technology | | | 9102S; RRID:AB_330744 | | | | | 1:1,000 | |
| Phospho-p44/42 MAP kinase (Thr202/Tyr204) | | Cell Signaling Technology | | | 9101S; RRID:AB_331646 | | | | | 1:1,000 | |
| Phospho-cortactin (Ser 405) | | A kind gift from Dr SA Weed | | | N/A | | | | | 1:1,000 | |
| Phospho-cortactin (Ser 418) | | A kind gift from Dr SA Weed | | | N/A | | | | | 1:1,000 | |
| Anti-cortactin (p80/85) clone 4F11 | | Millipore, Billerica, MA, USA | | | 05-180; RRID:AB_309647 | | | | | 1:1,000 (WB)  1:100 (IF) | |
| Prot-A purified mouse mAb OT17A2 LMP1 | | A kind gift from Prof. JM Middeldorp | | | N/A | | | | | 1:1,000 | |
| Anti-Cdc42 (mouse monoclonal IgG1) | | Millipore | | | 17-299; RRID:AB_390250 | | | | | 1:1,000 | |
| Anti-mouse IgG, HRP-linked | | Cell Signaling Technology | | | 7076S; RRID:AB_330924 | | | | | 1:3000 | |
| Anti-rabbit IgG, HRP-linked | | Cell Signaling Technology | | | 7074; RRID:AB_2099233 | | | | | 1:3,000 | |
| Anti-mouse IgG, Alexa Fluor 488 | | Millipore | | | A11059; RRID:AB_142495 | | | | | 1:1,000 | |
| Anti-rabbit IgG, Alexa Fluor 488 | | Millipore | | | A21206; RRID:AB_2535792 | | | | | 1:1,000 | |
| Brilliant Violet 605™ anti-human CD80 antibody | | BioLegend, San Diego, CA, USA | | | 305225; RRID:AB_11123909 | | | | | 1:200 | |
| Alexa Fluor® 647 anti-human CD163 antibody | | BioLegend | | | 326508; RRID:AB_893264 | | | | | 1:200 | |
| Anti-TNFα antibody (52B83) | | Santa Cruz Biotechnology | | | SC-52746;  RRID: AB_630341 | | | | | 1:100 | |
| Monoclonal anti-FLAG M2 antibody | | Sigma-Aldrich, Carlsbad, CA, USA | | | F1804;  RRID:AB_262044 | | | | | 1:1,000 | |
| Alexa Fluor 647 goat anti-mouse IgG | | Invitrogen, Inc., Carlsbad, CA, USA | | | A21235;  RRID:AB_2535804 | | | | | 1:1,000 | |
| Alexa Fluor 647 goat anti-rabbit IgG | | Invitrogen, Inc. | | | A21245  RRID:AB_141775 | | | | | 1:1,000 | |
| SH3PXD2A antibody | | Proteintech, Rosemont, IL, USA | | | 18976-1-AP | | | | | 1:100 | |
| N-WASP antibody | | Novus Biologicals, Littleton, CO, USA | | | H00008976-M04 | | | | | 1:100 | |
| **Peptides and Recombinant Proteins** | | | | | | | | | | | |
| **Name** | **Company** | | | | | | **Cat. No.** | | | | **Concentration** **(ng/ml)** |
| Recombinant murine BCA-1/BLC (CXCL13) | PeproTech, Rocky Hill, NJ, USA | | | | | | 250-24 | | | | 50 |
| Recombinant human ENA-78 (CXCL5) (5-78 a.a.) | PeproTech | | | | | | 300-22 | | | | 50 |
| Recombinant human eotaxin (CCL11) | PeproTech | | | | | | 300-21 | | | | 50 |
| Recombinant human FGF-6 | PeproTech | | | | | | 100-30 | | | | 50 |
| Recombinant human GRO-α/MGSA (CXCL1) | PeproTech | | | | | | 300-11 | | | | 50 |
| Recombinant human IL-6 | PeproTech | | | | | | 200-06 | | | | 50 |
| Recombinant human IL-10 | PeproTech | | | | | | 200-10 | | | | 50 |
| Recombinant human IP-10 (CXCL10) | PeproTech | | | | | | 300-12 | | | | 50 |
| Recombinant human LIF | PeproTech | | | | | | 300-05 | | | | 50 |
| Recombinant human MCP-3 (CCL7) | PeproTech | | | | | | 300-17 | | | | 20 |
| Recombinant human MIG (CXCL9) | PeproTech | | | | | | 300-26 | | | | 50 |
| Recombinant human MIP-4 (CCL18) | PeproTech | | | | | | 300-34 | | | | 20 |
| Recombinant human TIMP-1 | PeproTech | | | | | | 410-01 | | | | 20 |
| Recombinant human I-309 (CCL1) | PeproTech | | | | | | 300-37 | | | | 20 |
| Recombinant human IL-1β | PeproTech | | | | | | 200-01B | | | | 20 |
| Recombinant human GRO-β (CXCL2) | PeproTech | | | | | | 300-39 | | | | 20 |
| Recombinant human GRO-γ (CXCL3) | PeproTech | | | | | | 300-40 | | | | 20 |
| Recombinant human IL-8 (CXCL8) (72 a.a.) | PeproTech | | | | | | 200-08M | | | | 50 |
| Recombinant human MCP-1 (CCL2) | PeproTech | | | | | | 300-04 | | | | 50 |
| Recombinant human MIP-1β (CCL4) | PeproTech | | | | | | 300-09 | | | | 20 |
| Recombinant human RANTES (CCL5) | PeproTech | | | | | | 300-06 | | | | 50 |
| Recombinant human MIF | PeproTech | | | | | | 300-69 | | | | 50 |
| Recombinant human MIP-3α (CCL20) | PeproTech | | | | | | 300-29A | | | | 50 |
| Recombinant human osteopontin | PeproTech | | | | | | 120-35 | | | | 20 |
| Recombinant human GM-CSF | PeproTech | | | | | | 300-03 | | | | 50 |
| Recombinant human TNF-α | PeproTech | | | | | | 300-01A | | | | 10 |
| Recombinant M-CSF | PeproTech | | | | | | 300-25 | | | | 50 |
| Recombinant IL-4 | PeproTech | | | | | | 200-04 | | | | 20 |
| Recombinant IL-13 | PeproTech | | | | | | 200-13 | | | | 20 |
| **Chemicals** | | | | | | | | | | | |
| **Name** | **Company** | | | | | | **Cat. No.** | | | | |
| Lipopolysaccharides from *Escherichia coli* O111:B4 | Sigma-Aldrich, Carlsbad, CA, USA | | | | | | L3024 | | | | |
| Interferon γ | Genescript, Piscataway, NJ, USA | | | | | | Z02915 | | | | |
| RNAscope 2.5 HD Duplex Reagent Kit | ACDBio, Newark, CA, USA | | | | | | 322435 | | | | |
| CD68 RNA probe | ACDBio | | | | | | 560591-c2 | | | | |
| TNFα RNA probe | ACDBio | | | | | | 310421 | | | | |
| Phorbol 12-myristate 13-acetate | Sigma-Aldrich | | | | | | P8139 | | | | |
| Gelatin From Pig Skin, Fluorescein Conjugate | Thermo Fisher Scientific | | | | | | G13187 | | | | |
| Gelatin | Sigma-Aldrich | | | | | | G8150 | | | | |
| Alexa Fluor™ 568 Phalloidin | Thermo Fisher Scientific | | | | | | A12380 | | | | |
| DAPI | Sigma-Aldrich | | | | | | D1306 | | | | |
| U0126 | Millipore | | | | | | 19-147 | | | | |
| Src inhibitor-1 | Sigma-Aldrich | | | | | | S2075 | | | | |
| Erlotinib | Millipore | | | | | | SML2156 | | | | |
| 187-1 | Millipore | | | | | | 681660 | | | | |
| GST-CBD N-WASP binding beads | A kind gift from Prof. Gareth Jones | | | | | | N/A | | | | |
| **Experimental models: cell lines** | | | | | | | | | | | |
| NPC43 | | | | [23] | | | | | | | |
| NP460 | | | | [24] | | | | | | | |
| **Reagents for cloning** | | | | | | | | | | | |
| **Name** | | | **Company** | | | | | | **Cat. No.** | | |
| Q5® High-Fidelity DNA Polymerase | | | NEB, Ipswich, MA, USA | | | | | | M0491L | | |
| Q5® Site-Directed Mutagenesis Kit | | | NEB | | | | | | E0554S | | |
| T4 DNA Ligase | | | NEB | | | | | | M0202M | | |
| PCR Cloning System with Gateway™ Technology with pDONR™221 & OmniMAX™2 Competent Cells | | | Thermo Fisher Scientific | | | | | | 12535029 | | |
| EcoRI | | | NEB | | | | | | R0101M | | |
| NheI | | | NEB | | | | | | R3131M | | |
| AgeI | | | NEB | | | | | | R3552L | | |
| BamHI | | | NEB, Ipswich | | | | | | R0136M | | |
| **Oligonucleotides** | | | | | | | | | | | |
| **Sequence** | | | | | | **Source** | | | | | |
| EBFP2.N1 Src forward primer (5′ to 3′): | | | | | | This paper (Addgene) | | | | | |
| TGCTTAGAATTCATGGGTAGCAACAA | | | | | |
| EBFP2.N1 Src reverse primer (5′ to 3′): | | | | | | This paper (Addgene) | | | | | |
| TAAGCAACCGGTGGGAGGTTCTCCCCGGGCTG | | | | | |
| pEF1α-IRES-ZsGreen1 Src forward primer (5′ to 3′): | | | | | | This paper (Addgene) | | | | | |
| TAAGCAGCTAGCATGGGTAGCAACAAGAGCAAG | | | | | |
| pEF1α-IRES-ZsGreen1 Src reverse primer (5′ to 3′): | | | | | | This paper (Addgene) | | | | | |
| TGCTTAGAATTCCTAGAGGTTCTCCCCGGGCTG | | | | | |
| EBFP2.C1 LMP1 forward primer (5′ to 3′): | | | | | | This paper (Addgene) | | | | | |
| TAAGCAGAATTCCATGGAACGCGAC | | | | | |
| EBFP2.C1 LMP1 reverse primer (5′ to 3′): | | | | | | This paper (Addgene) | | | | | |
| TGCTTAGGATCCTTAGTCATAGTAG | | | | | |
| iRFP670.C1 Cdc42 forward primer (5′ to 3′): | | | | | | This paper (Addgene) | | | | | |
| GGGGACAAGTTTGTACAAAAAAGCAGGCTTCGCCACCATGGCGCGTAAGGTCGATCT | | | | | |
| iRFP670.C1 Cdc42 reverse primer (5′ to 3′): | | | | | | This paper (Addgene) | | | | | |
| GGGGACCACTTTGTACAAGAAAGCTGGGTCTCATAGCAGCACACACCTGCGGCTCT | | | | | |
| Site-directed mutagenesis of Src Y419F forward primer: | | | | | | This paper (Addgene) | | | | | |
| AGACAATGAGTTTACGGCGCGGC | | | | | |
| Site-directed mutagenesis of Src Y419F reverse primer: | | | | | | This paper (Addgene) | | | | | |
| TCAATGAGCCGAGCCAGC | | | | | |
| Site-directed mutagenesis of Src Y530F forward primer: | | | | | | This paper (Addgene) | | | | | |
| CGAGCCCCAGTTTCAGCCCGGGG | | | | | |
| Site-directed mutagenesis of Src Y530F reverse primer: | | | | | | This paper (Addgene) | | | | | |
| GTGGACGTGAAGTAGTCCTCCAGGAAGG | | | | | |
| **Recombinant DNA** | | | | | | | | | | | |
| **Name** | | | | **Source** | | | | **Number** | | | |
| EBFP2.N1 | | | | Addgene | | | | 54595 | | | |
| EBFP2.C1 | | | | Addgene | | | | 54665 | | | |
| EGFP-Cdc42-wt | | | | Addgene | | | | 12599 | | | |
| EGFP-Cdc42-Q61L | | | | Addgene | | | | 12600 | | | |
| EGFP-Cdc42-T17N | | | | Addgene | | | | 12601 | | | |
| piRFP670-N1 | | | | Addgene | | | | 45457 | | | |
| pEF1α-IRES-ZsGreen1 | | | | Clontech, Mountain View, CA, USA | | | | 631976 | | | |
| PLPCX 2117 LMP1 | | | | [30] | | | |  | | | |
| **Software and algorithms** | | | | | | | | | | | |
| **Name** | | | | **Source** | **Homepage** | | | | | | |
| GraphPad Prism 8 | | | | GraphPad Software, Inc., San Diego, CA, USA | https://www.graphpad.com/scientific-software/prism/ | | | | | | |
| FlowJo v10 | | | | FlowJo, LLC, Ashland, OR, USA | https://www.flowjo.com/solutions/flowjo | | | | | | |
| ZEN 2.3 (Blue edition) | | | | Carl Zeiss, Thornwood, NY, USA | https://www.zeiss.com/microscopy/int/products/microscope-software/zen-lite.html | | | | | | |
| IN Cell Analyzer v7.3 | | | | GE Healthcare, Waukesha, WI, USA |  | | | | | | |
| Imaris 9.5.1 software | | | | Bitplane, South Windsor, CT, USA | https://imaris.oxinst.com/versions/9-5 | | | | | | |
| ImageJ (Fiji v. 2.0.0-rc-65/1.51w) | | | | [37] | https://imagej.net/Fiji | | | | | | |
| ClustVis | | | | [60] | https://biit.cs.ut.ee/clustvis/ | | | | | | |

**Contact for Reagent and Resource Sharing:** All requests for reagents and resources should be directed to the lead contact, Dr Anna Tsang (annatsang@cuhk.edu.hk).
